# Phenalenyl-fused porphyrins with different ground states†
†Electronic supplementary information (ESI) available: Synthetic procedures and characterization data of all new compounds; general methods for all physical characterizations; DFT calculation details; additional spectroscopic and crystallographic data. CCDC 1019251, 1019261 and 1019262. For ESI and crystallographic data in CIF or other electronic format see DOI: 10.1039/c4sc03866e


**DOI:** 10.1039/c4sc03866e

**Published:** 2015-02-04

**Authors:** Wangdong Zeng, Sangsu Lee, Minjung Son, Masatoshi Ishida, Ko Furukawa, Pan Hu, Zhe Sun, Dongho Kim, Jishan Wu

**Affiliations:** a Department of Chemistry , National University of Singapore , 3 Science Drive 3 , 117543 , Singapore . Email: chmwuj@nus.edu.sg ; Fax: +65 6779 1691; b Spectroscopy Laboratory for Functional π-Electronic Systems and Department of Chemistry , Yonsei University , Seoul 120-749 , Korea . Email: dongho@yonsei.ac.kr; c Education Center for Global Leaders in Molecular Systems for Devices , Kyushu University , Fukuoka 819-0395 , Japan; d Center for Instrumental Analysis , Institute for Research Promotion , Niigata University , Nishi-ku , Niigata 950-2181 , Japan; e Institute of Materials Research and Engineering , A*STAR , 3 Research Link , Singapore 117602

## Abstract

Materials based on biradicals/biradicaloids have potential applications for organic electronics, photonics and spintronics. In this work, we demonstrated that hybridization of porphyrin and polycyclic aromatic hydrocarbon could lead to a new type of stable biradicals/biradicaloids with tunable ground state and physical property. Mono- and bis-phenalenyl fused porphyrins **1** and **2** were synthesized *via* an intramolecular Friedel–Crafts alkylation-followed-by oxidative dehydrogenation strategy. Our detailed experimental and theoretical studies revealed that **1** has a closed-shell structure with a small biradical character (*y* = 0.06 by DFT calculation) in the ground state, while **2** exists as a persistent triplet biradical at room temperature under inert atmosphere. Compound **1** underwent hydrogen abstraction from solvent during the crystal growing process while compound **2** was easily oxidized in air to give two dioxo-porphyrin isomers **11a**/**11b**, which can be correlated to their unique biradical character and spin distribution. The physical properties of **1** and **2**, their dihydro/tetrahydro-precursors **7**/**10**, and the dioxo-compounds **11a**/**11b** were investigated and compared.

## Abstract 2

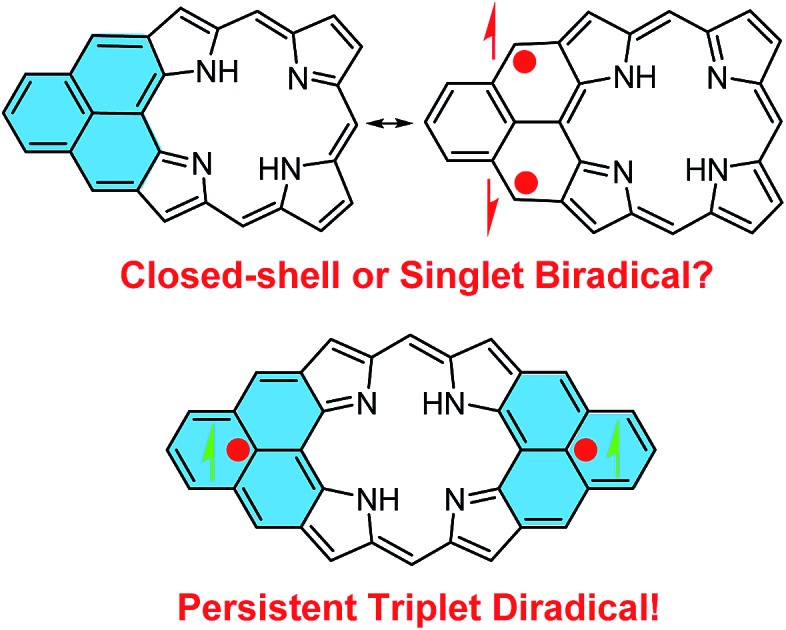
Fusion of one or two phenalenyl units onto the porphyrin core led to biradicaloids with different ground state, physical property and chemical reactivity.

## Introduction

**Fig. 1:**
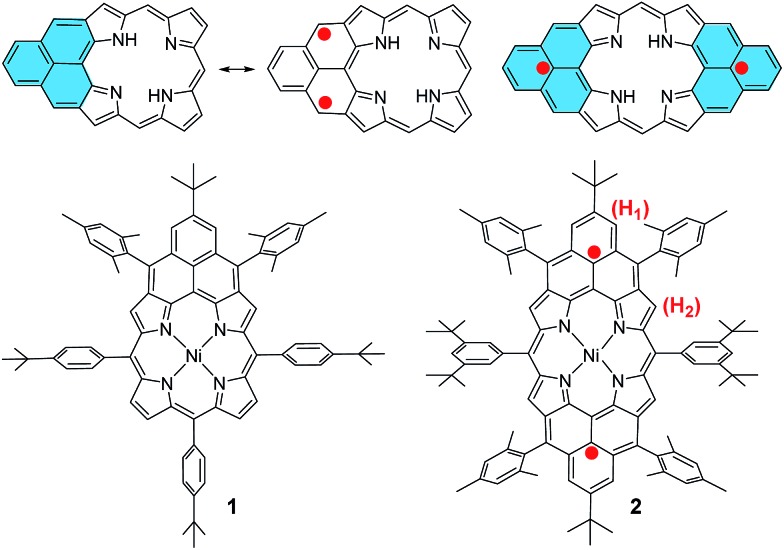
Structures of mono- and bis-phenalenyl fused porphyrin biradicaloids and their derivatives **1** and **2**.

Stable π-conjugated biradicaloids have recently attracted intensive research interest due to their unique electronic, optical and magnetic properties and potential applications in organic electronics, non-linear optics, spintronics, and energy storage devices.^1^ Typical examples include bis(phenalenyls),^2^ zethrenes,^3^ indenofluorenes,^4^ extended *p*-quinodimethanes and vilogen,^5^ quinoidal oligothiophenes and thienoacenes,^6^ and zig-zag edged graphene molecules.^7^ In addition, a doubly linked corrole dimer^8^ and a *meso*-diketo-hexaphyrin^9^ were also reported by Osuka *et al.* to be singlet biradicaloids in the ground state. Among the various designs, phenalenyl monoradical^10^ as the smallest open-shell polycyclic aromatic hydrocarbon (PAH) showing remarkable thermodynamic stability has been used for the design of stable biradicaloids such as bis(phenalenyls)^2^ and zethrenes.^3^ Our particular interest here is to develop a new type of *hybrid* biradicals/biradicaloids by fusion of one or two phenalenyl units onto an aromatic porphyrin skeleton (Fig. 1). Although various PAH-fused porphyrins have been reported,^11^ none of them showed open-shell biradical character. The *mono*-phenalenyl fused porphyrin molecule can be drawn at least in two resonance structures, one closed-shell form containing a non-aromatic porphyrin, and an open-shell biradical form possessing an aromatic porphyrin unit (Fig. 1). Thus, it may be a singlet biradicaloid in the ground state. The *bis*-phenalenyl fused porphyrin however can only be drawn in an open-shell biradical form (Fig. 1). This difference raises the curiosity about their ground state, chemical reactivity and physical properties. Like all other biradicaloids, kinetic blocking of the high spin density sites is necessary for obtaining stable/persistent materials, thus the bulky mesityl-blocked and mono- and bis-phenalenyl fused Ni-porphyrins **1** and **2** (Fig. 1) were synthesized and investigated in this work.

## Results and discussion

### Synthesis

**Scheme 1:**
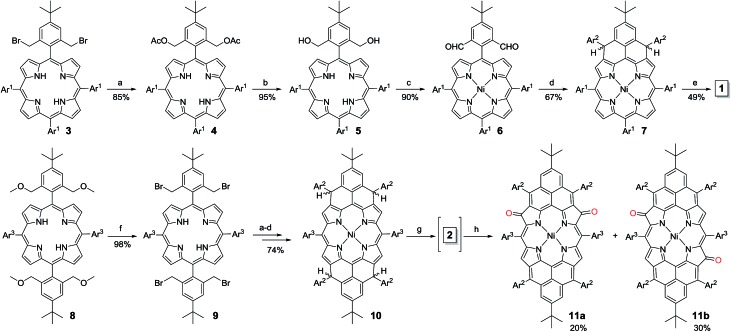
Reagents and conditions: (a) KOAc, CH_3_CN, THF, reflux, 1 day; (b) LiOH·2H_2_O, dioxane, H_2_O, reflux, 2 days; (c) (i) oxalyl chloride, DMSO, DCM, Et_3_N; (ii) Ni(acac)_2_, toluene, reflux, 24 h; (d) (i) mesitylmagnesium bromide, THF, rt, 24 h; (ii) excess BF_3_·Et_2_O, DCM, 10 min; (e) NIS, DCM, 49%; (f) (i) HBr, AcOH, DCM, rt, (ii) NaHCO_3_ (aq.); (g) *p*-chloranil, DCM; (h) air. Ar^1^: 4-*tert*-butylphenyl, Ar^2^: mesityl, Ar^3^: 3,5-di-*tert*-butylphenyl.

The fusion of one or two phenalenyl units onto the porphyrin core was successfully achieved by an intramolecular Friedel–Crafts alkylation-followed-by-oxidative dehydrogenation strategy (Scheme 1). For the synthesis of **1**, the 2,6-bis(bromomethyl)-4-*tert*-butylphenyl substituted porphyrin **3** (ref. 12) was transformed into the Ni-porphyrin dialdehyde **6** in high yield *via* an esterification–hydrolysis–Swern oxidation–metallation sequence. Compound **6** was treated with mesitylmagnesium bromide to give the intermediate diol, which was subjected to a Friedel–Crafts alkylation reaction promoted by BF_3_·OEt_2_ to afford the dihydro-precursor **7** in 67% yield. Compound **1** was then obtained as a red solid in 49% yield by oxidative dehydrogenation of **7** with *N*-iodosuccinimide (NIS). It is worthy to note that the obtained Ni-porphyrin **1** showed largely enhanced stability compared to its zinc- and free base porphyrin analogues, which are very sensitive in air and difficult to separate. A similar strategy was used for the synthesis of **2**. Treatment of the tetra(methylether)-substituted porphyrin **8** (ref. 13) with HBr in AcOH gave the tetra(bromomethyl)-substituted porphyrin **9**, which underwent a similar esterification–hydrolysis–Swern oxidation–metallation–nucleophilic addition–Friedel–Crafts alkylation sequence to afford the tetrahydro-porphyrin precursor **10** in an overall 72% yield. Oxidative dehydrogenation of **10** with *p*-chloranil in dry dichloromethane (DCM) gave an air sensitive species (compound **2**, *vide infra*) which could not be isolated in pure form. Upon exposure to air for 3 h, the reactive species was transformed into two dioxo-porphyrin isomers **11a** (purple solid) and **11b** (red solid), which can be separated by routine column chromatography in 20% and 30% yield, respectively.

### Ground states of **1** and **2**

**Fig. 2:**
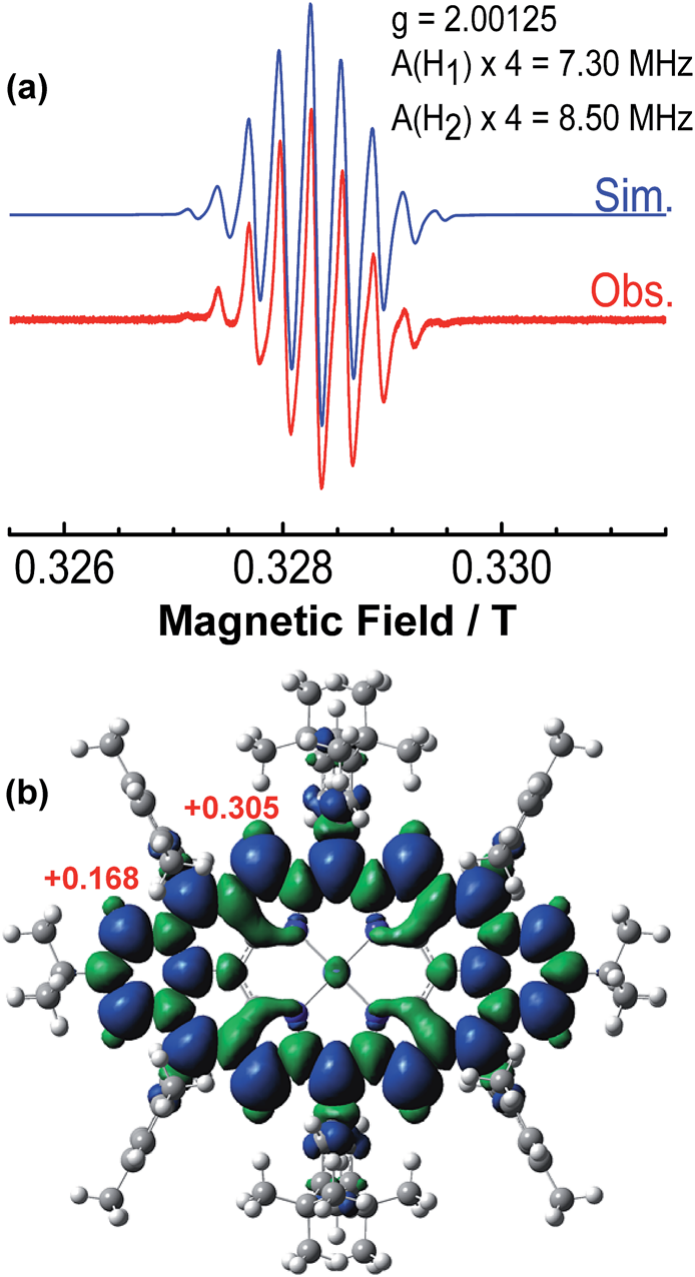
(a) ESR spectrum of the *in situ* generated **2** recorded at 298 K and the simulated spectrum. (b) Calculated spin density distribution of the triplet biradical **2** (UB3LYP/6-31G*). Blue and green surfaces represent positive and negative spin densities, respectively.

The intermediate species was identified to be the desired compound **2** as a triplet biradical. After addition of *p*-chloranil under argon, the colour of the solution changed from light-green to dark-brown in 10 minutes at room temperature (RT). High-resolution APCI mass spectroscopic measurement of the solution gave a peak at *m*/*z* = 1522.8235 ([M]^+^; calcd for **2** C_108_H_112_N_4_Ni: 1522.8240), indicating the successful removal of four hydrogens. The absorption spectrum of the solution showed a broad red-shifted absorption extending beyond 1200 nm, which is consistent with the calculated electronic transitions by UB3LYP method (Fig. S1 in ESI†). ESR measurement of the solution clearly exhibited a well-resolved spectrum (*g*_e_ = 2.00125) (Fig. 2a), with a relative ESR intensity of 94% to the DPPH standard under the same concentration (**10** and DPPH) and the same measurement conditions. Broken symmetry density functional theory (DFT) (UB3LYP/6-31G*) calculations suggested that compound **2** has an open-shell triplet ground state ( has an open-shell triplet ground state (〈*s*^2^〉 = 2.1194) with singlet-triplet energy gap (Δ = 2.1194) with singlet-triplet energy gap (Δ*E*_S–T_) of +6.98 kcal mol^–1^ based on Yamaguchi equation. This is reasonable since the structure of **2** cannot be drawn as a closed-shell resonance form, like many other reported triplet biradicals.^14^ On the basis of the molecular orbital (MO) characteristics, the triplet biradical **2** shows *non-disjoint* nature of the non-bonding molecular orbitals to avoid the Coulomb repulsion by filing the MO with two electrons, which prefer to the triplet electronic structure. The spin density map suggests that it is a π-electron based biradical species since the spin density on Ni atom is negligible (Fig. 2b). Simulation of the ESR spectrum was conducted by taking consideration of the spin-nucleus coupling with the four protons on the two fused benzene rings (H_1_ in Fig. 1) and the four β-protons on the pyrrole rings (H_2_ in Fig. 1), both possessing high spin densities (*ρ*(H_1_) = +0.168, *ρ*(H_2_) = +0.305, Fig. 2b). The simulated ESR spectrum (*A*(H_1_) = 7.30 MHz, *A*(H_2_) = 8.50 MHz) of the triplet biradical **2** was in good agreement with the experimentally observed spectrum (Fig. 2a). The VT ESR measurements on the frozen solution (173–113 K) revealed broadened ESR spectra with a hyperfine structure (Fig. S2 in ESI†), which can be well reproduced by similar spin Hamiltonian parameters (*A*(H_1_) = 8 MHz, *A*(H_2_) = 9 MHz, at 153 K). At the same time, the ESR intensity (*I*) increased with decreasing temperature (*T*), with *I* being approximately proportional to 1/*T*. The solvent was removed under nitrogen and the solid sample was also submitted to the temperature dependent ESR measurements (298–113 K) (Fig. S2 in ESI†). Similar to the frozen solution, broadened ESR spectra were observed and the ESR intensity showed a very good linear relationship to the 1/*T*. All these observations together with the DFT calculations supported a triplet biradical character of **2**. However, The forbidden half-field Δ*m*_s_ = ±2 transition ESR spectrum was not observed due to the large delocalization of the spin, which was also observed in other delocalized biradical systems.^3^,^8^,^9^ It is worth noting that there was no obvious change in spectral shape upon standing at RT in argon for 7 h except for a slight decrease in intensity, indicating good persistence of the triplet biradical under inert atmosphere, which is remarkable for a triplet biradical and can be explained by the efficient spin delocalization along the whole π-conjugated system. However, the pure biradical of **2** could not be isolated even after we tried different ways. The calculated large spin density at the β-pyrrolic carbon atoms also suggested the high reactivity of these sites. In fact, the biradical **2** can be easily oxidized by oxygen when stirring in air and mainly gave two stable dioxo-porphyrins **11a** and **11b** (Scheme 1).

**Fig. 3:**
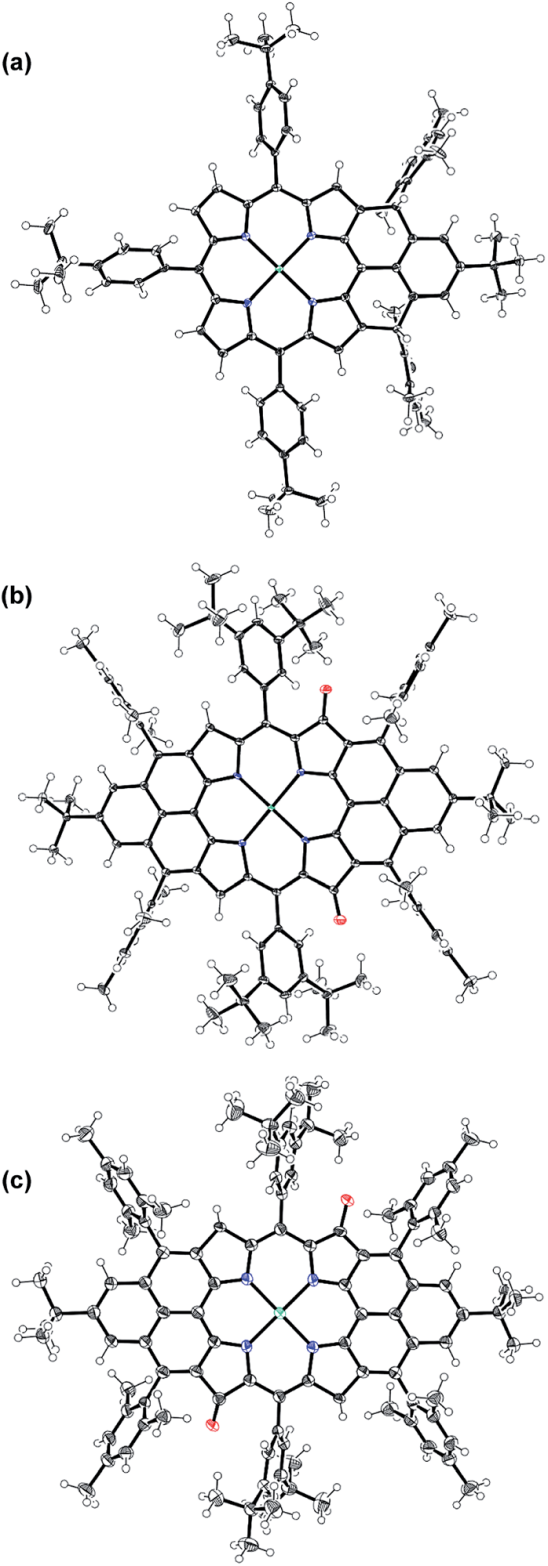
X-ray crystallographic structures of **1-H2** (a), **11a** (b) and **11b** (c). Solvent molecules are omitted for clarity; ellipsoids are set to 50% probability.

Compound **1** was identified as a closed-shell structure in the ground state based on the observation of sharp NMR spectrum even at elevated temperatures (Fig. S3 in ESI†) and it is also supported by DFT calculations (**1**_(CS)_ ∼ **1**_(SB)_ > **1**_(TB)_; biradical character *y* = 0.06; = 0.06; 〈*s*^2^〉 = 0.0003; Δ = 0.0003; Δ*E*_S–T_ = –4.28 kcal mol^–1^ based on UB3LYP/6-31G* calculation). Attempted single crystal growth by slow diffusion of CH_3_CN into a solution of **1** in toluene however resulted in the dihydrogenated product **1-H_2_** which was confirmed by the crystallographic analysis (Fig. 3a) and high resolution APCI mass (*m*/*z* = 1155.5776 ([M^+^]; calcd for C_80_H_81_N_4_Ni: 1155.5809)).‡
‡Crystallographic data for **1-H_2_**: C_85.50_H_87_N_5_Ni, *M*_w_ = 1243.31; triclinic; space group *P*1[combining macron]; *a* = 15.0498(12) Å, *b* = 15.6708(13) Å, *c* = 17.4668(15) Å, *α* = 111.901(3)°, *β* = 107.076(3)°, *γ* = 102.416(3)°; *V* = 3400.2(5) Å^3^; *Z* = 2; *ρ*_calcd_ = 1.214 Mg m^–3^; *R*_1_ = 0.0441 (*I* > 2*σ*(*I*)), w*R*_2_ = 0.1134 (all data). CCDC no. 1019262. Crystallographic data for **11a**: C_136_H_142_N_4_NiO_2_, *M*_w_ = 1923.24; triclinic; space group *P*1[combining macron]; *a* = 12.8361(14) Å, *b* = 15.5717(18) Å, *c* = 15.9281(17) Å, *α* = 100.921(4)°, *β* = 109.976(4)°, *γ* = 108.180(4)°; *V* = 2682.0(5) Å^3^; *Z* = 1; *ρ*_calcd_ = 1.191 Mg m^–3^; *R*_1_ = 0.0506 (*I* > 2*σ*(*I*)), w*R*_2_ = 0.1184 (all data). CCDC no. 1019251. Crystallographic data for **11b**: C_136_H_142_N_4_NiO_2_, *M*_w_ = 1923.24; triclinic; space group *P*1[combining macron]; *a* = 12.8500(12) Å, *b* = 15.5881(15) Å, *c* = 15.9283(15) Å, *α* = 100.951(5)°, *β* = 110.033(5)°, *γ* = 108.118(5)°; *V* = 2687.2(5) Å^3^; *Z* = 1; *ρ*_calcd_ = 1.188 Mg m^–3^; *R*_1_ = 0.0771 (*I* > 2*σ*(*I*)), w*R*_2_ = 0.2360 (all data). CCDC no. 1019261. Oxygen atom disorder was observed in **11a** but the structure can be identified by ^1^H NMR and TD DFT calculations, and it is the only possible closed-shell dioxo-isomer besides **11b**. The hydrogenation selectively took place at the two reactive sites with high spin densities in the phenalenyl unit (Fig. 1). Crystal growth in anhydrous toluene/CH_3_CN, DCM/CH_3_CN, and benzene/CH_3_CN all gave the dihydro-compound **1-H_2_**, indicating that CH_3_CN likely is the hydrogen source. Compound **1** decomposed in protonic solvents such as methanol and ethanol. The structures of **11a** and **11b** were also identified by X-ray crystallographic analysis (Fig. 3b and c), implying that they are two isomers which differ only in the positions of the two oxygen atoms.‡ Both complexes have a flat central π-conjugated framework. **11b** is *centro*-symmetric with Ni sitting on the inversion center but **11a** is not. They are also the only two possible closed-shell structures that can be drawn for the oxidized products of **2** when the oxidation takes places at two of the four β-pyrrolic positions. Compounds **11a** and **11b** also showed different NMR spectra, which can be well assigned to their isomeric structures (ESI†).

### Optical and electrochemical properties

**Fig. 4:**
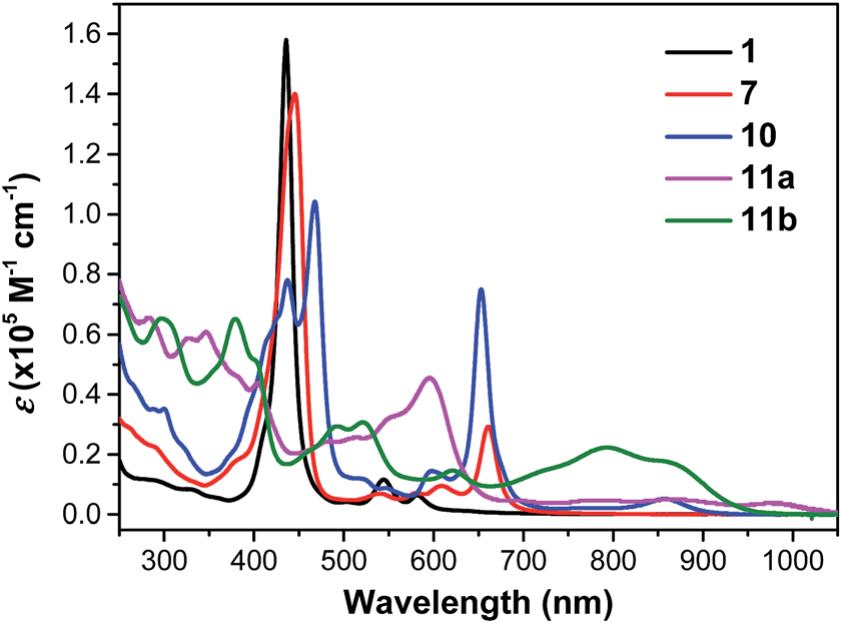
UV-vis-NIR absorption spectra of **1**, **7**, **10**, **11a** and **11b** in DCM.

Compound **1** shows one intense Soret band at 436 nm and two weak Q bands at 545 and 582 nm in DCM, which are blue-shifted compared with the dihydro-precursor **7** (Fig. 4 and Table 1). Such a difference can be explained by partial destruction of aromaticity of the porphyrin ring after fusion of a phenalenyl unit. The ^1^H NMR spectrum of compound **1** however mainly showed an aromatic character possibly due to the contribution of the diradical form and a zwitterionic resonance form to the ground state (Fig. S4 in ESI†).^15^ The two dioxo-porphyrin isomers **11a** and **11b** exhibit very different absorption spectra in the UV-vis-NIR region (Fig. 4). The *cis*-isomer **11a** displays a red-shifted absorption spectrum compared with the *trans*-isomer **11b** presumably due to its asymmetric push–pull character. The observed band shape and intensity are well in agreement with the time-dependent DFT calculations for these two isomers (Fig. S5 and S6 in ESI†). Owing to their extended π-conjugation and intramolecular donor–acceptor interactions, both **11a** and **11b** have a smaller optical energy gap (1.17 eV for **11a** and 1.27 eV for **11b**) compared with compound **10** (1.37 eV).

**Table 1:** Photophysical and electrochemical data of the porphyrin derivatives **1**, **7**, **10**, **11a** and **11b**^*a*^

	*λ*_max_ (nm)	*ε*_max_ (M^–1^ cm^–1^)	*E* 1/2 ox (V)	*E* 1/2 red (V)	HOMO (eV)	LUMO (eV)	*E* EC g (eV)	*E* Opt g (eV)	*τ* (ps)	*σ* ^(2)^ (GM)
**1**	436	158 100	0.50		–5.27	–3.18	2.09	1.96	300	—
	544	11 600	0.93	–1.71						
	582	600	1.11	–2.16						
			1.30							

**7**			0.26		–4.98	–3.64	1.34	1.66		370 (1300 nm)
	446	139 800	0.47	–0.95					1.1 (*τ*_1_)	
	662	29 000	0.65	–2.18					4.2 (*τ*_2_)	
			1.08							

**10**	436	77 500	0.02		–4.73	–3.62	1.11	1.37		
	467	103 800	0.17	–0.90					4 (*τ*_1_)	780 (1300 nm)
	652	74 400	0.57	–1.29					32 (*τ*_2_)	250 (1700 nm)
	858	5200	0.79	–1.72						
			0.93							

**11a**	346	61 000	0.31	–1.13	–5.00	–3.74	1.26	1.17	2.4 (*τ*_1_),	1000 (1200 nm)
	595	45 700	0.95	–1.54					11.3 (*τ*_2_)	
	997	3500								

**11b**	380	46 000			–5.02	–3.66	1.36	1.27	8.9	980 (1600 nm)
	490	29 200	0.31	–1.19						
	523	30 600	0.99	–1.52						
	793	22 400	1.21							
	862	17 400								

^*a*^ *ε*_max_: molar extinction coefficient at the absorption maximum. *E*1/2ox and *E*1/2red are half-wave potentials of the oxidative and reductive waves, respectively, with potentials *versus* Fc/Fc^+^ couple. HOMO and LUMO energy levels were calculated according to equations: HOMO = –(4.8 + *E*onsetox) eV and LUMO = –(4.8 + *E*onsetred) eV, where *E*onsetox and *E*onsetred are the onset potentials of the first oxidative and reductive redox wave, respectively. *E*ECg: electrochemical energy gap derived from LUMO–HOMO. *E*Optg: optical energy gap derived from lowest energy absorption onset in the absorption spectra. *τ*: excited lifetime based on the TA measurements. *σ*^(2)^: TPA cross section.

**Fig. 5:**
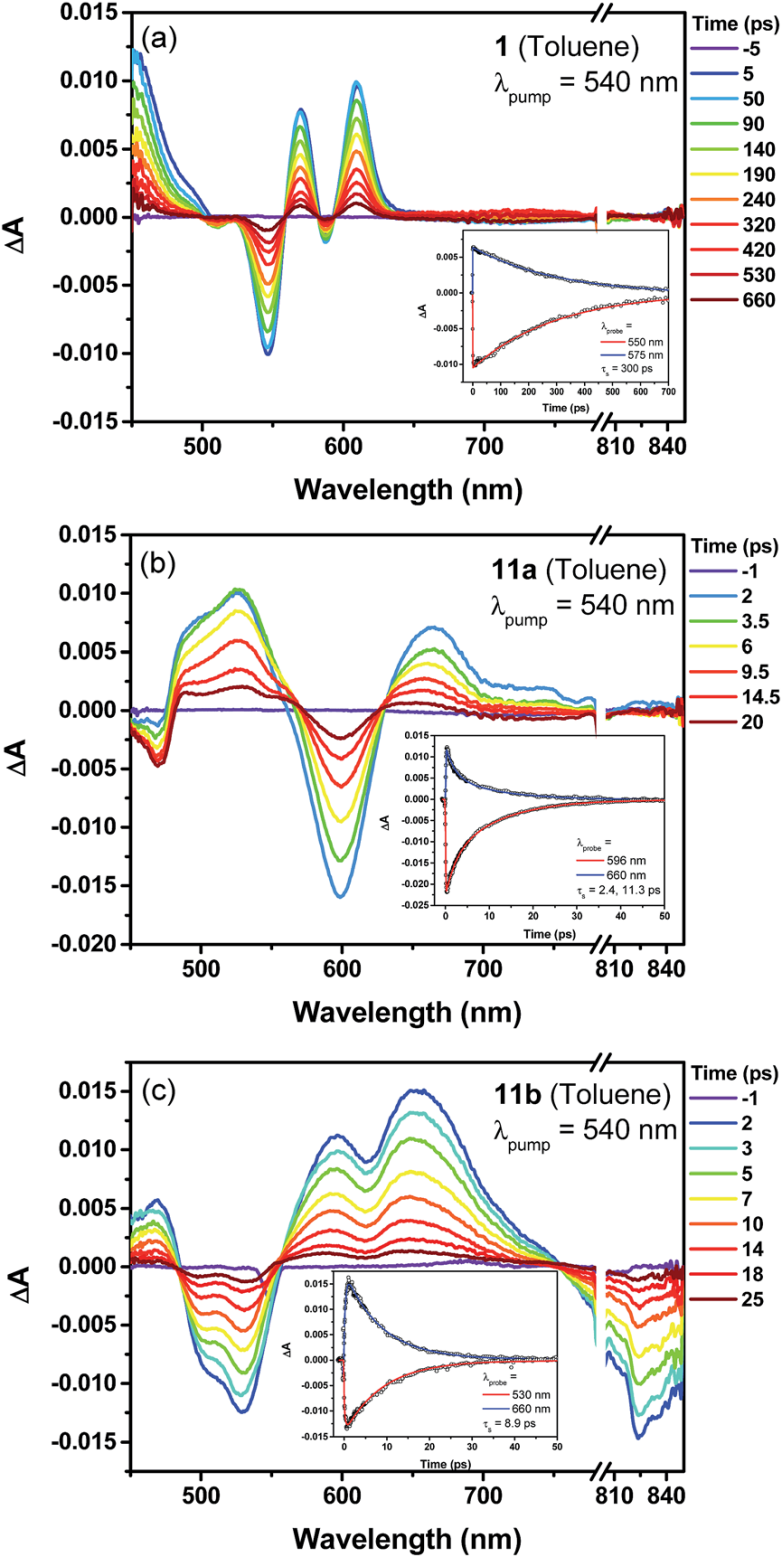
Femtosecond transient absorption spectra and decay profiles (inset) of **1** (a), **11a** (b) and **11b** (c) in toluene measured at room temperature (296 K). For all, the excitation wavelength is 540 nm.

The excited-state dynamics of compound **1**, the dihydro- (**7**) and tetrahydro- (**10**) precursors, and the dioxo-porphyrins **11a**/**11b** were probed by femto-second transient absorption (TA) measurements (Fig. 5 and Fig. S10 in ESI†). The TA spectrum of **1** exhibited a ground-state bleaching signal around 545 nm together with two excited-state absorption bands at 570 and 610 nm, which is distinct from the dihydro-compound **7** (Fig. S10 in ESI†). At the same time, much longer singlet excited state lifetimes was determined for **1** (*τ* = 300 ps) than **7** (*τ* = 14.2 ps). This is out of our expectation since chromophore with small and moderate biradical character was theoretically predicted to show shorter singlet excited lifetime and larger two-photon absorption (TPA) cross sections.^16^ Such an unusual trend could be ascribed to the relatively larger energy gap of **1** compared with **7**. The two dioxo-porphyrin isomers **11a** and **11b** exhibit very different TA spectra (Fig. 5) and both show shorter singlet excited state lifetime (*τ* = 11.3 ps for **11a** and 8.9 ps for **11b**) compared with the tetrahydro-porphyrin **10** (*τ* = 32 ps).

Due to the extended π-conjugation, compounds **11a** and **11b** also showed good third order non-linear susceptibility with large TPA cross sections in the near infrared region, with *σ*^(2)^ = 1000 GM when excited at 1200 nm for **11a** and *σ*^(2)^ = 980 GM when excited at 1600 nm for **11b**, both are larger than the tetrahydro-precursor **10** (*σ*^(2)^ = 780 GM at 1300 nm and *σ*^(2)^ = 250 GM at 1700 nm) (Fig. S11 and S12 in ESI†).§
§Our TPA set-up is 1200–2400 nm and we cannot measure at OPA wavelengths shorter than 600 nm. Therefore, the TPA data of compound **1** was not obtained..

All the porphyrin compounds (**1**, **7**, **10**, **11a**, **11b**) showed multiple oxidation and reduction waves in the cyclic voltammetry and differential pulse voltammetry (Table 1 and Fig. S13 and S14 in ESI†) and the measured HOMO/LUMO energy levels and energy gaps are consistent with the DFT calculations and optical data (Fig. S7–S9, and Table S5 in ESI†).

## Conclusion

In summary, phenalenyl-fused porphyrins **1** and **2** were synthesized by an intramolecular Friedel–Crafts alkylation-followed-by-oxidative dehydrogenation protocol. The *mono*-phenalenyl fused porphyrinoid **1** turned out to have a closed-shell ground state but the contribution of the biradical form makes it easy to be hydrogenated during the crystal growing process. The *bis*-phenalenyl fused porphyrinoid **2** cannot be drawn in a closed-shell structure and thus is a triplet biradical. It is persistent in inert atmosphere but can be easily oxidized into two dioxo-porphyrins in air. The observed physical properties and chemical reactivity can be well correlated to their biradical character and spin density distribution. Our research provided a good example of how to develop stable/persistent hybrid biradicaloids.

## Supplementary Material

Supplementary information

Crystal structure data
