# Supplementary material for: Phenalenyl-fused porphyrins with different ground states
Source: Chem Sci. 2015 Feb 4;6(4):2427–33. doi: 10.1039/c4sc03866e (PMC6085729; doi:10.1039/c4sc03866e)
Supplement: Supplementary file 1 [file SC-006-C4SC03866E-s001.pdf]

Electronic Supplementary Material (ESI) for Chemical Science.
This journal is © The Royal Society of Chemistry 2015

Electronic Supplementary Information for

Phenalenyl-fused Porphyrins with Different Ground States

Wangdong Zeng, Sangsu Lee, Minjung Son, Masatoshi Ishida, Ko Furukawa, Pan Hu, Zhe Sun, Dongho Kim, Jishan Wu**

Table of Contents

1. Experimental Section

   1.1 General....................S2

   1.2 Synthetic procedures and characterization data S4

2. Absorption and ESR spectra of triplet biradical **2**....................S14
3. VT NMR spectrum of compound **1**....................S16
4. DFT calculations....................S16
5. Transient absorption spectra and Z-scan curves....................S21
6. Electrochemical data....................S23
7. NMR spectra and HR mass spectra…....................S24
8. Crystallographic data of **1-H$_2$**, **11a** and **11b**....................S57

1. Experimental Section

1.1 General

All reagents and starting materials were obtained from commercial suppliers and used without further purification. Anhydrous toluene and dichloromethane (DCM) were distilled under a nitrogen atmosphere over sodium and calcium hydride, respectively. 2,6-Bis(methoxymethyl)-4-*tert*-butyl benzaldehyde **12** (Scheme S1) and 2,6-bis(bromomethyl)-4-*tert*-butylphenyl substituted porphyrin **3** were prepared according to the literature (N. Jux, *Org. Lett.* **2000**, *2*, 2129). Column chromatography was performed on silica gel 60 (Merck 40-60 nm, 230-400 mesh). All NMR spectra were recorded on the Bruker AMX500 spectrometer. All chemical shifts are quoted in ppm, relative to tetramethylsilane, using the residual solvent peak as a reference standard. Atmospheric Pressure Chemical Ionization (APCI MS) was performed on a Finnigan TSQ 7000 triple stage quadrupole mass spectrometer. MALDI-TOF mass spectra were measured on a Bruker Autoflex MALDI-TOF instrument using tetracyanoquinodimethane (TCNQ) as a matrix. High resolution mass spectra (HR MS) were recorded on a Finnigan MAT95XL-T with FAB ionization source or recorded on Finnigan MAT 95×P spectrometer. UV-vis-NIR absorption and fluorescence spectra were recorded on a Shimadzu UV-1700/UV-3600 spectrophotometer and a RF-5301 fluorometer, respectively. Cyclic voltammetry (CV) measurements were performed in dry DCM on a CHI 620C electrochemical analyzer with a three-electrode cell, using 0.1 M Bu_4NPF_6 as supporting electrolyte, AgCl/Ag as reference electrode, gold disk as working electrode, Pt wire as counter electrode, and scan rate at 50 mV/s. The potential was externally calibrated against the ferrocene/ferrocenium couple. Continuous wave X-band ESR spectra were obtained with a Bruker ELEXSYS E500 spectrometer using a variable temperature Bruker liquid nitrogen cryostat.

The femtosecond time-resolved transient absorption (*fs*-TA) spectrometer consists of an optical parametric amplifier (OPA; Palitra, Quantronix) pumped by a Ti:sapphire regenerative amplifier system (Integra-C, Quantronix) operating at 1 kHz repetition rate and an optical detection system. The generated OPA pulses have a pulse width of ~ 100 fs and an average power of 1 mW in the range of 280-2700 nm, which are used as pump pulses. White light continuum (WLC) probe pulses were generated using a sapphire window (3 mm thick) by focusing a small portion of the fundamental 800 nm pulses which was picked off by a quartz plate before entering the OPA. The time delay between

pump and probe beams was carefully controlled by making the pump beam travel along a variable optical delay (ILS250, Newport). Intensities of the spectrally dispersed WLC probe pulses are monitored by a High Speed Spectrometer (Ultrafast Systems) for both visible and near-infrared measurements. To obtain the time-resolved transient absorption difference signal (ΔA) at a specific time, the pump pulses were chopped at 500 Hz and absorption spectra intensities were saved alternately with or without pump pulse. Typically, 4000 pulses excite the samples to obtain the fs-TA spectra at each delay time. The polarization angle between pump and probe beam was set at the magic angle (54.7°) using a Glan-laser polarizer with a half-wave retarder in order to prevent polarization-dependent signals. Cross-correlation *fwhm* in pump-probe experiments was less than 200 fs and chirp of WLC probe pulses was measured to be 800 fs in the 400-800 nm region. To minimize chirp, all reflection optics in the probe beam path and a quartz cell of 2 mm path length were used. After fs-TA experiments, the absorption spectra of all compounds were carefully examined to detect if there were artifacts due to degradation and photo-oxidation of samples. The three-dimensional data sets of ΔA versus time and wavelength were subjected to singular value decomposition and global fitting to obtain the kinetic time constants and their associated spectra using Surface Xplorer software (Ultrafast Systems).

The two-photon absorption spectrum was measured in the NIR region using the open-aperture Z-scan method with 130 fs pulses from an optical parametric amplifier (Light Conversion, TOPAS) operating at a repetition rate of 1 kHz generated from a Ti:sapphire regenerative amplifier system (Spectra-Physics, Hurricane). After passing through a 10 cm focal length lens, the laser beam was focused and passed through a 1 mm quartz cell. Since the position of the sample cell could be controlled along the laser beam direction (*z* axis) using the motor controlled delay stage, the local power density within the sample cell could be simply controlled under constant laser intensity. The transmitted laser beam from the sample cell was then detected by the same photodiode as used for reference monitoring. The on-axis peak intensity of the incident pulses at the focal point, I_0, ranged from 40 to 60 GW cm^{-2}. For a Gaussian beam profile, the nonlinear absorption coefficient can be obtained by curve fitting of the observed open-aperture traces $T(z)$ with the following equation:

$$T(z) = 1 - \frac{\beta I_0 (1 - e^{-\alpha_0 l})}{2\alpha_0 [1 + (z/z_0)^2]}$$

where α_0 is the linear absorption coefficient, l is the sample length, and z_0 is the diffraction length of the

incident beam. After the nonlinear absorption coefficient has been obtained, the TPA cross section $\sigma^{(2)}$ of one solute molecule (in units of GM, where 1 GM = 10^{-50} cm^4 s photon^{-1} molecule^{-1}) can be determined by using the following relationship:

$$\beta = \frac{10^{-3}\sigma^{(2)}N_A d}{h\nu}$$

where N_A is the Avogadro constant, d is the concentration of the compound in solution, h is the Planck constant, and ν is the frequency of the incident laser beam.

1.2 Synthetic procedures and characterization data

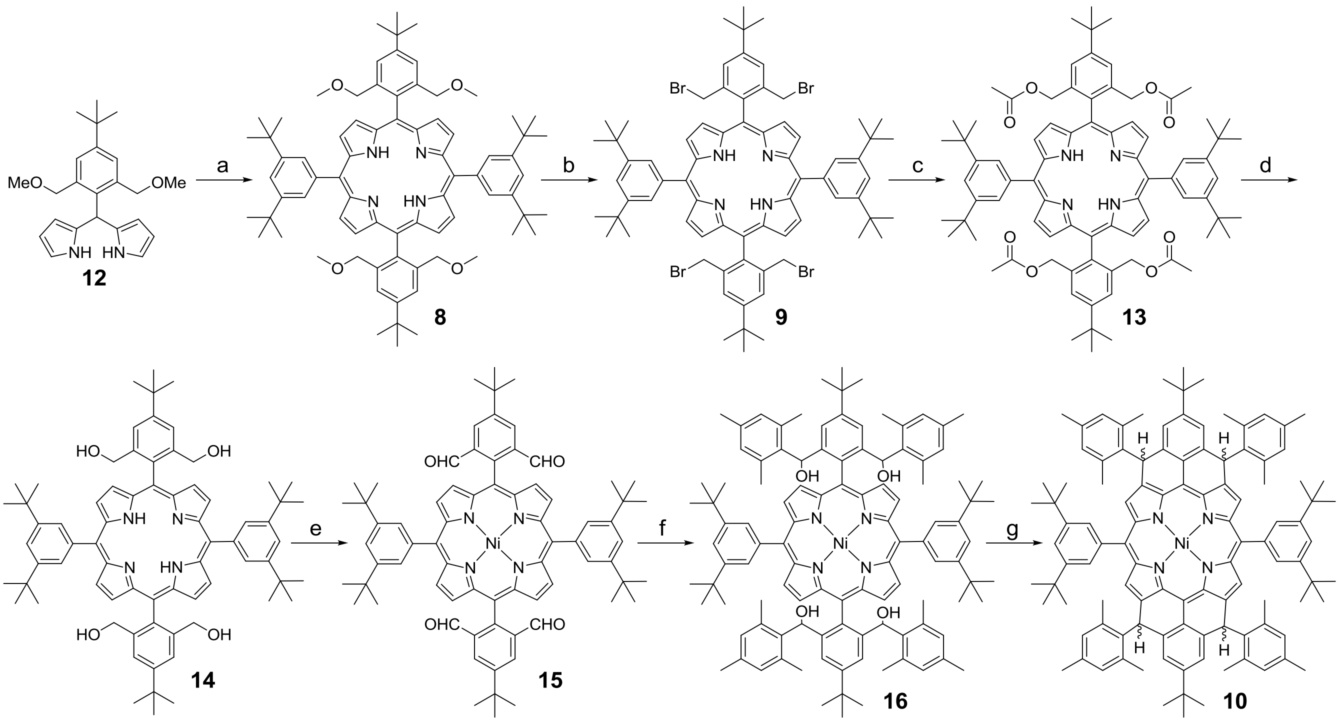

Scheme S1. Synthesis of precursor **10**. *Reagents and conditions*: (a) i) 3,5-di-*tert*-butylbenzaldehyde, BF_3•OEt_2, CH_2Cl_2 (+1% EtOH), rt; ii) DDQ, 22%; (b) i). HBr, AcOH, CH_2Cl_2, rt, ii. $NaHCO_3$, H_2O, 98%; (c) KOAc, CH_3CN, THF, reflux, 2d, 85% ; (d) LiOH, H_2O, dioxane, H_2O, reflux, 2d, 96%; (e) i) oxalyl chloride, DMSO, DCM, Et_3N, 96%; ii) Ni(acac)$_2$, toluene, reflux, 24h, 97%; (f) mesitylmagnesium bromide, THF, rt, 24h, 65%; g) excess BF_3•OEt_2, CH_2Cl_2, 10 min, 90%.

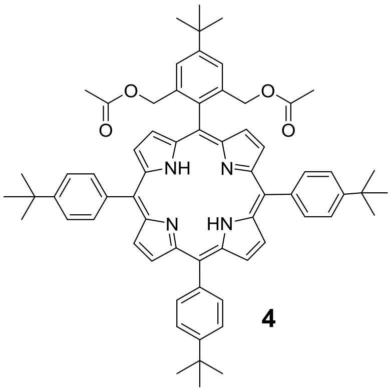

4

Compound **3** (1g, 0.98 mmol), KOAc (1.44 g, 14.7 mmol) and tetra-*n*-butylammonium bromide (3.31 g, 1.47 mmol) were dissolved in a mixture of 200 mL of THF and 200 mL of CH_3CN under argon atmosphere. The mixture was reflux for 1 day and poured into 200 mL of ice water. The layers were separated, and the organic layer was washed with water for several times and dried over $MgSO_4$. The solvent was removed under vacuum and the residue was purified by column chromatography (silica gel, DCM) to afford the desired product **4** (0.83 g) in 85% yield. ^1H NMR ($CDCl_3$, 500 MHz): δ 8.88 (d, 4H, *J* = 4.5 Hz), 8.85 (d, 2H, *J* = 4.5 Hz), 8.61 (d, 2H, *J* = 4.5 Hz), 8.17 (m, 6H), 7.80 (s, 2H), 7.78 (m, 6H), 1.65 (s, 9H), 1.63 (s, 27 H), 1.42 (s, 6H), -2.67 (s, 2H, 2 NH); ^{13}C NMR ($CDCl_3$, 125 MHz): δ 170.11, 152.07, 150.60, 139.36, 139.13, 138.47, 137.64, 134.54, 125.16, 123.70, 121.04, 120.33, 112.79, 65.40, 34.98, 21.78, 31.70, 20.27. HR-MS (APCI): *m*/*z* = 983.5491, calcd. for $C_{66}H_{71}N_4 O_4$ (M+1): *m*/*z* = 982.5470, error = -0.8 ppm.

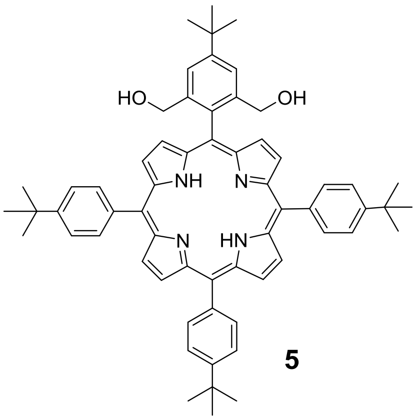

5

To a solution of ester **4** (820 mg, 0.828 mmol) in dioxane (200 mL) and THF (40 mL) were added $LiOH{\cdot}H_2O$ (15.4 g, 41.4 mmol) and water (90 mL). The mixture was refluxed for 24 h. After cool down to room temperature, ethyl acetate (200 mL) and 200 mL of ice water was added. The layers were separated and the organic layer was dried over $MgSO_4$. The solvent was removed under vacuum and the

residue was purified by column chromatography (silica gel, ethyl acetate) to afford the desired product **5** (700 mg) in 95% yield. ^{1}H NMR ($CDCl_3$, 500 MHz): δ 8.87 (m, 6H), 8.62 (d, 2H, *J* = 6.0 Hz), 8.15 (br, 6H), 7.89 (s, 2H), 7.77 (br, 6H), 4.69 (s, 2H, 2 OH), 4.18 (m, 4H), 1.64 (s, 9H), 1.61 (s, 27H), -2.69 (s, 2H, 2NH); ^{13}C NMR ($CDCl_3$, 125 MHz): 152.32, 152.11, 150.56, 150.51, 142.27, 141.83, 139.13, 138.85, 138.75, 136.97, 134.38, 124.32, 124.09, 123.55, 123.41, 120.82, 120.35, 120.25, 113.06, 65.33, 64.03, 63.96, 34.82, 31.60. HR-MS (APCI): *m/z* = 899.5259, calcd. for $C_{62}H_{67}N_4O_2$(M+1) : *m/z* = 899.5186, error = 0.1 ppm.

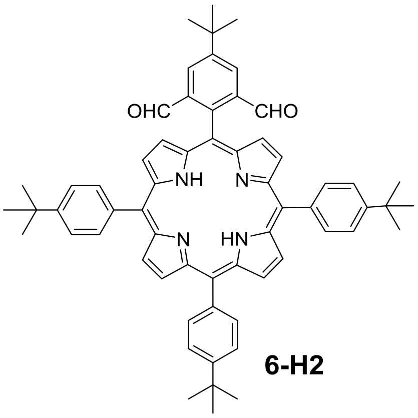

6-H2

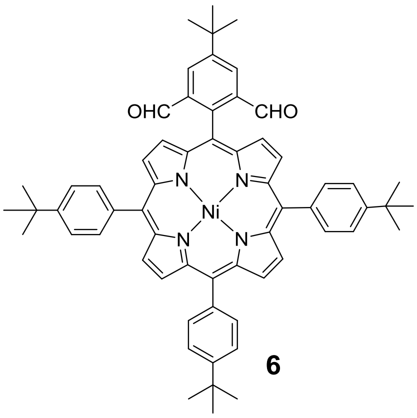

6

A solution of oxalyl chloride (1.67 mL, 19.5 mmol) in 90 mL of freshly distilled CH_2Cl_2 was cooled to -78 °C, and DMSO (2.72 mL, 38.4 mmol) was carefully added under nitrogen atmosphere. After stirring for 15 min, compound **5** (700 mg, 0.78 mmol) in 100 mL of CH_2Cl_2 was added and the mixture was stirred at -78 °C for 2h. Et_3N (5.44 mL) was added successively and the solution was stirred for 1h at -78 ^{o}C. The cooling bath was then removed, and the reaction mixture was allowed to warm to room temperature and stirred for 30 min. The solvent was removed under reduced pressure, and the residue was extracted with ethyl acetate. The extract was washed with saturated aqueous Na_2CO_3 solution, brine, and dried over anhydrous Na_2SO_4. After removal of the solvent under reduced pressure the residue was purified by column chromatography (silica gel, hexane/DCM = 2/1) to afford the metal-free porphyrin **6-H2** (675 mg) in 97% yield. A mixture of **6-H2** (400 mg, 0.45 mmol) and nickel(II) acetylacetonate (0.46 g, 1.93 mmol) in toluene (80 mL) was heated at reflux for 24 h. The solution was cooled down to room temperature and washed with water, dried over anhydrous sodium sulfate. The solvent was removed under vacuum and the residue was purified by column chromatography (silica gel, DCM) to afford the nickel complex **6** in 90% yield (380 mg).

Compound **6-H2**: ^{1}H NMR ($CDCl_3$, 500 MHz): δ 9.34 (s, 2H), 8.91 (d, 2H, *J* = 5.0 Hz), 8.90 (d, 4H, *J* =

5.0 Hz), 8.63 (s, 2H), 8.52 (d, 2H, *J* = 4.5 Hz), 8.15 (m, 6H), 7.79 (m, 6H), 1.69 (s, 9H), 1.62 (s, 27H), -2.59 (s, 2NH); ^{13}C NMR ($CDCl_3$, 125 MHz): 190.12, 153.11, 150.84, 145.29, 138.94, 138.62, 138.52, 134.54, 127.48, 123.77, 123.69, 121.80, 121.32, 107.38, 35.62, 34.95, 31.71, 31.42. HR-MS (APCI): *m/z* = 895.4972, calcd. for $C_{62}H_{67}N_4O_2$ (M+1): *m/z* = 895.4946, error = -3.0 ppm.

Compound **6**: ^{1}H NMR ($CDCl_3$, 500 MHz): δ 9.26 (s, 2H), 8.81-8.80 (m, 6H), 8.55 (s, 2H), 8.41 (d, 2H, *J* = 8.0 Hz), 7.56 (d, 2H, *J* = 4.0 Hz), 7.93 (d, 4H, *J* = 8.0 Hz), 7.12 (d, 2H, *J* = 4.5 Hz), 7.69 (d, 4H, *J* = 8 Hz), 1.63 (s, 9H), 1.56-1.55 (m, 27H); ^{13}C NMR ($CDCl_3$, 125 MHz): 190.07, 152.99, 150.76, 150.72, 143.86, 143.40, 143.30, 142.74, 137.76, 137.46, 137.33, 137.70, 133.46, 132.85, 132.76, 130.83, 127.91, 123.83, 120.08, 119.93, 106.82, 35.46, 34.81, 31.55, 31.26. HR-MS (APCI): *m/z* = 951.4176, calcd. for $C_{62}H_{61}N_4NiO_2$(M+1) : *m/z* = 951.4143, error = -3.5 ppm.

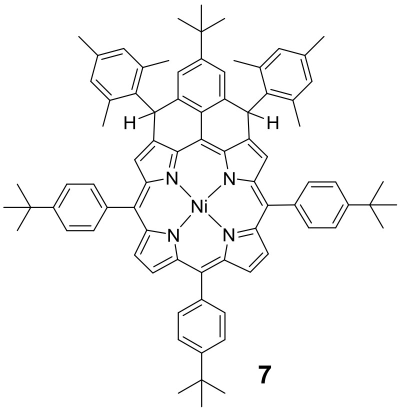

7

Compound **6** (350 mg, 0.37 mmol) was dissolved in 30 mL of THF under argon, 2-mesitylmagnesium bromide solution (5.55 mL, 5.55 mmol, 1.0 M in diethyl ether) was added and the solution was stirred for 36 h at room temperature. The mixture was then poured into ice water with vigorous stirring, extracted by DCM (100 mL). The organic layer was washed by water, then dried over Na_2SO_4. The solvent was removed under vacuum, and the crude product (diol) was washed by DCM/MeOH (100/1) to afford a red solid which was used for the next step directly (330 mg). Boron trifluoride diethyl etherate (1.5 mL) was added to a solution of the as-prepared red solid (330 mg, 0.23 mol) in DCM (20 mL) at room temperature under argon and the mixture turned green immediately. After stirring for 10 min, methanol (10 mL) and water (20 mL) were added to quench the reaction. The organic layer was separated and washed by water, dried over Na_2SO_4. The solvent was removed under vacuum and the residue was purified by column chromatography (silica gel, hexane/DCM = 3/1) to afford the desired product **7** in 67% yield (288 mg) for two steps. ^{1}H NMR ($CDCl_3$, 500 MHz): δ 8.33 (d, 1H, *J* = 4.5 Hz), 8.17 (d, 1H, *J* = 5.0 Hz), 8.15 (d, 1H, *J* = 5 Hz), 7.90 (d, 1H, *J* = 5 Hz), 7.82 (br, 4H), 7.64 (t, 6H, *J* =

8Hz), 7.50 (d, 2H, *J* = 8 Hz), 7.19 (s, 1H), 7.16 (s, 2H), 6.80 (s, 2H), 5.01-4.82 (m, 2H), 2.85 (s, 3H), 2.51 (s, 3H), 2.40 (s, 3H), 2.12 (s, 3H), 1.99 (s, 3H), 1.55 (s, 9H), 1.55-1.54 (m, 27H), 1.42 (s, 3H); ^{13}C NMR ($CDCl_3$, 125 MHz): δ 155.45, 150.44, 150.25, 148.45, 148.37, 144.38, 142.84, 142.39, 140.57, 139.96, 138.31, 137.99, 137.95, 137.83, 137.67, 136.85, 139.79, 136.69, 135.92, 134.59, 133.13, 132.82, 132.67, 132.55, 132.14, 131.68, 131.15, 129.61, 128.67, 128.64, 128.57, 128.43, 128.02, 127.19, 127.09, 126.31, 125.32, 124.06, 123.89, 123.05, 121.99, 118.65, 109.62, 105.43, 42.15, 41.06, 34.83, 31.65, 21.51, 21.34, 21.05, 20.02, 19.88. HR-MS (APCI): *m/z* = 1155.5776, calcd. for $C_{80}H_{81}N_4Ni$ (M-1): *m/z* = 1155.5809, error = 2.9 ppm.

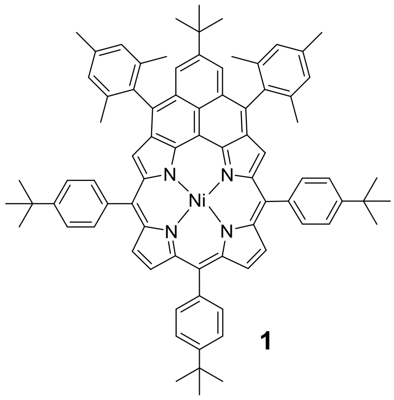

1

Compound **7** (100 mg, 0.086 mmol) was dissolved in 20 mL of CH_2Cl_2 under argon, *N*-iodosuccinimide (38.9 mg, 0.17 mmol) was added and the mixture was stirred for 5 min at room temperature. The color of the solution tuned to brown. The solvent was removed by vacuum and the residue was purified by column chromatography (silica gel, hexane/DCM = 5/1) to give the red coloured compound **1** in 49% yield (49 mg). ^{1}H NMR ($CDCl_3$, 500 MHz): δ 8.71 (d, 2H, *J* = 5.0 Hz), 8.64 (d, 2H, *J* = 5.0 Hz), 8.51 (s, 2H), 7.97 (d, 2H, *J* = 8.0 Hz), 7.94 (d, 2H, *J* = 7.5 Hz), 7.68 (d, 6H, *J* = 8.0 Hz), 7.28 (s, 2H), 7.17 (s, 2H), 6.78 (s, 2H), 6.73 (s, 2H), 2.91 (S, 6H), 2.39 (s, 6H), 1.58 (m, 27H), 1.42 (s, 6H), 1.21 (s, 9H); ^{13}C NMR ($CDCl_3$, 125 MHz): δ 150.93, 150.33, 150.23, 143.32, 143.29, 143.19, 142.01, 139.17, 138.73, 138.34, 138.31, 138.22, 137.95, 137.03, 136.42, 136.03, 133.64, 132.27, 131.37, 131.08, 128.49, 128.21, 125.85, 123.86, 123.68, 123.27, 119.34, 119.05, 110.10, 42.42, 34.84, 34.56, 31.65, 29.72, 21.61, 21.00, 20.03. HR-MS (APCI): *m/z* = 1153.5662, calcd. for $C_{80}H_{79}N_4Ni$ (M+1): *m/z* = 1153.5653, error = -0.8 ppm.

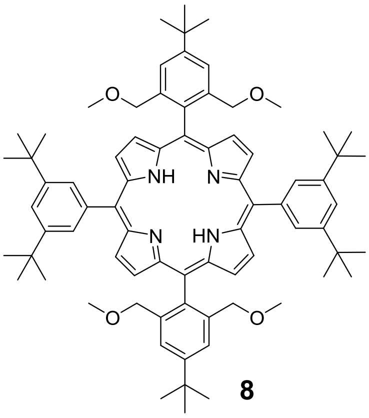

8

4-*tert*-Butyl-2,6-bis(methoxymethyl)phenyl-dipyrromethane **12** (3.66 g, 10 mmol) and 3,5-di-*tert*-butylbenzaldehyde (2.18 g, 10 mmol) were dissolved in 2 L of dry CH_2Cl_2 containing 20 mL of dry EtOH and the solution was purged with argon for 30 min. BF_3•OEt_2 (0.84 mL, 6.6 mmol) was added and the mixture was stirred at room temperature for 2 h in the dark. DDQ (3.40 g, 15 mmol) was then added and the reaction mixture stirred for another 2 h. The solvent was removed under vacuum and the residue was purified by column chromatography (silica gel, hexane/DCM = 1/2) to give the desired product **8** in 22% yield (1.24 g). ^{1}H NMR ($CDCl_3$, 500 MHz): δ 8.88 (d, 4H, *J* = 8.0 Hz), 8.69 (d, 4H, *J* = 8.0 Hz), 8.11 (d, 4H, *J* = 3.0 Hz), 7.88 (s, 4H), 7.86 (s, 2H), 3.96 (s, 8H), 2.78 (s, 12H), 1.63 (s, 18H), 1.53 (s, 36H), -2.58 (s, 2NH); ^{13}C NMR ($CDCl_3$, 125 MHz): δ 151.80, 148.85, 140.59, 139.46, 135.33, 130.18, 122.20, 121.14, 120.98, 114.63, 72.98, 58.06, 34.99, 31.65. HR-MS (APCI): *m*/*z* = 1127.7368, calcd. for $C_{76}H_{75}N_4O_4$ (M+1): *m*/*z* = 1127.7348, error = -1.8 ppm.

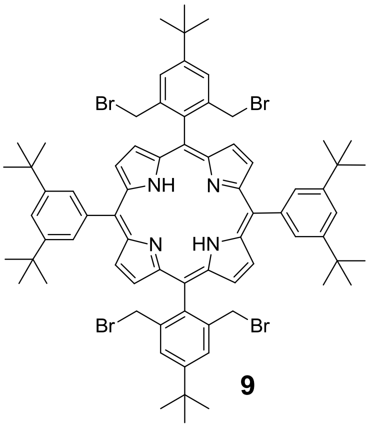

9

Porphyrin **8** (1.2 g, 1.06 mmol) was dissolved in 200 mL of CH_2Cl_2. 300 mL of a 33% solution of HBr in glacial acetic acid was added and the mixture was stirred at room temperature overnight. The reaction was quenched with water, and the organic layer was washed with a saturated Na_2CO_3 solution and dried over $MgSO_4$. The solvent was removed under vacuum and the residue was purified by column

chromatography (silica gel, hexane/DCM = 2/1) to afford the desired product **9** in 98% yield (1.37 g). ^{1}H NMR ($CDCl_3$, 500 MHz): δ 8.92 (d, 2H, *J* = 8.0 Hz), 8.90 (d, 2H, *J* = 8.0 Hz), 8.67 (d, 4H, *J* = 8.0 Hz), 8.12 (m, 3H), 7.90 (m, 3H), 7.79 (s, 2H), 7.08 (s, 2H), 4.11 (s, 8H), 1.64 (s, 18H), 1.54 (s, 36H), -2.55 (s, 2NH); ^{13}C NMR ($CDCl_3$, 125 MHz): δ 152.90, 148.93, 140.57, 139.36, 138.06, 130.39, 129.11, 127.20, 125.38, 121.97, 121.10, 112.80, 35.15, 35.07, 32.41, 31.74, 31.52, 29.37, 23.20. HR-MS (APCI): *m*/*z* = 1319.3364, calcd. for $C_{72}H_{83}Br_4N_4$ (M+1): *m*/*z* = 1319.3346, error = -1.4 ppm.

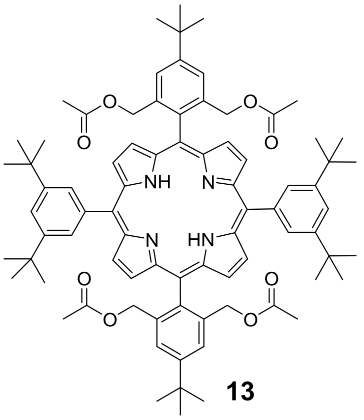

13

Compound **9** (1g, 0.76 mmol), KOAc (2.96 g, 30.2 mmol) and tetra-*n*-butylammonium bromide (7.2 g, 3.02 mmol) were dissolved in a mixture of THF (400 mL) and CH_3CN (400 mL) under argon atmosphere. The mixture was reflux for 2 days and after cooling down to room temperature, it was poured into 500 mL of ice water. The organic layer was washed with water for several times and dried over $MgSO_4$. The solvent was removed under vacuum and the residue was purified by column chromatography (silica gel, DCM) to afford the desired product **13** in 85% yield (0.81 g). ^{1}H NMR ($CDCl_3$, 500 MHz): δ 8.84 (d, 4H, *J* = 8.0 Hz), 8.62 (d, 4H, *J* = 8.0 Hz), 8.08 (d, 4H, *J* = 8.0 Hz), 7.79 (m, 6H), 4.67 (s, 8H), 1.62 (s, 18H), 1.56 (s, 12H), 1.52 (s, 36H), -2.64 (s, 2NH); ^{13}C NMR ($CDCl_3$, 125 MHz): δ 170.06, 151.90, 148.75, 140.77, 138.47, 137.39, 130.05, 125.11, 124.60, 124.06, 121.48, 120.88, 113.35, 65.27, 34.98, 31.64, 31.60, 31.53, 20.18. HR-MS (APCI): *m*/*z* = 1239.7142, calcd. for $C_{80}H_{95}N_4O_8$ (M+1): *m*/*z* = 1239.7144, error = 0.2 ppm.

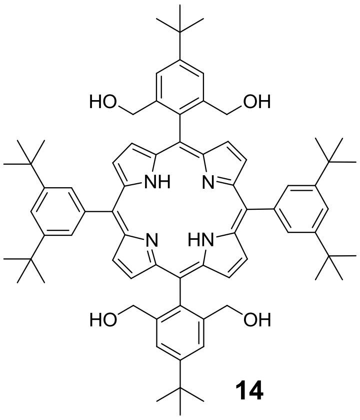

14

To a solution of ester **13** (800 mg, 0.64 mmol) in dioxane (250 mL) and THF (50 mL) were added LiOH•H_2O (23.8 g, 64 mmol) and water (100 mL). The mixture was refluxed for 36 h under nitrogen. After cooling down to room temperature, ethyl acetate (200 mL) and ice water (200 mL) was added. The organic layer was dried over $MgSO_4$ and the solvent was removed under vacuum. The residue was purified by column chromatography (silica gel, ethyl acetate) to afford the desired product **14** in 96% yield (658 mg). ^{1}H NMR ($CDCl_3$, 500 MHz): δ 8.87 (d, 4H, *J* = 4.5 Hz), 8.67 (d, 4H, *J* = 4.5 Hz), 8.07 (s, 4H), 7.90 (s, 4H), 7.80 (s, 2H), 4.24 (s, 8H), 1.64 (s, 18H), 1.52 (s, 36H), -2.55 (s, 2NH). HR-MS (APCI): *m*/*z* = 1071.6722, calcd. for $C_{72}H_{87}O_4N_4$ (M+1): *m*/*z* = 1071.6722, error = 0.0 ppm.

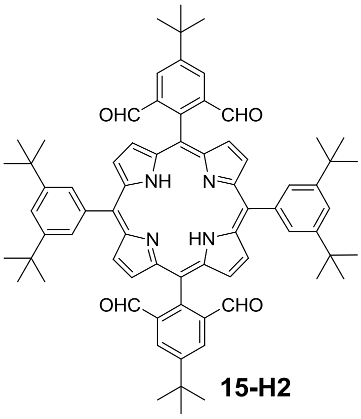

15-H2

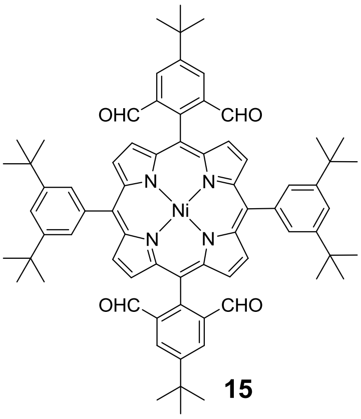

15

A solution of oxalyl chloride (2.62 mL, 30.5 mmol) in 100 mL of freshly distilled CH_2Cl_2 was cooled to -78 °C, and DMSO (4.25 mL, 60 mmol) was carefully added under nitrogen atmosphere. After stirring for 15 min, a solution of compound **14** (650 mg, 0.61 mmol) in CH_2Cl_2 (100 mL) was added and the mixture was stirred at -78 °C for 2h. Et_3N (8.5 mL) was added successively and the solution was stirred at -78 ^{o}C for 1h. Then the cooling bath was removed, and the reaction mixture was allowed to warm to room temperature and stirred for 30 min. The solvent was removed under vacuum and the residue was extracted with ethyl acetate. The extract was washed with saturated aqueous Na_2CO_3 solution, brine,

and dried over anhydrous Na_2SO_4. After removal of the solvent under reduced pressure, the crude product was purified by column chromatography (silica gel, hexane/DCM = 1/1) to afford the metal-free porphyrin **15-H2** in 96% yield (622 mg). A mixture of **15-H2** (500 mg, 0.47 mmol) and nickel(II) acetylacetonate (1.21 g, 4.7 mmol) in toluene (150 mL) was heated at reflux for 24 h. The solution was cooled down to room temperature and washed with water, dried over anhydrous sodium sulfate. The solvent was removed and the residue was purified by column chromatography (silica gel, DCM) to afford nickel complex **15** in 97% yield (510 mg). Compound **15-H2**: ^1H NMR ($CDCl_3$, 500 MHz): δ 9.37 (s, 4H), 8.93 (d, 4H, J = 4.5 Hz), 8.64 (s, 4H), 8.57 (d, 4H, J = 4.5 Hz), 8.07 (s, 4H), 7.82 (s, 2H), 1.68 (s, 18H), 1.53-1.52 (m, 36H), -2.41 (s, 2NH); ^{13}C NMR ($CDCl_3$, 125 MHz): δ 189.96, 153.39, 149.22, 144.95, 140.10, 138.51, 130.14, 128.17, 123.62, 121.54, 109.16, 46.17, 35.65, 35.14, 31.75, 31.41. HR-MS (APCI): m/z = 1063. 6117, calcd. for $C_{72}H_{79}N_4O_4$ (M+1): m/z = 1063.6096, error = -2.0 ppm. Compound **16**: ^1H NMR ($CDCl_3$, 500 MHz): δ 9.28 (s, 4H), 8.44 (d, 4H, J = 5.0 Hz), 8.57 (s, 4H), 8.48 (d, 4H, J = 5.0 Hz), 7.88 (s, 4H), 7.75 (s, 2H), 1.63 (s, 18H), 1.47 (s, 36H); ^{13}C NMR ($CDCl_3$, 125 MHz): δ 189.92, 153.27, 149.20, 143.80, 143.63, 139.09, 137.79, 134.45, 131.52, 128.95, 128.14, 122.38, 121.52, 108.35, 35.51, 35.01, 31.62, 31.27. HR-MS (APCI): m/z = 1119.5305, calcd. for $C_{72}H_{77}N_4NiO_4$ (M+1): m/z = 1119.5293, error = -1.1ppm.

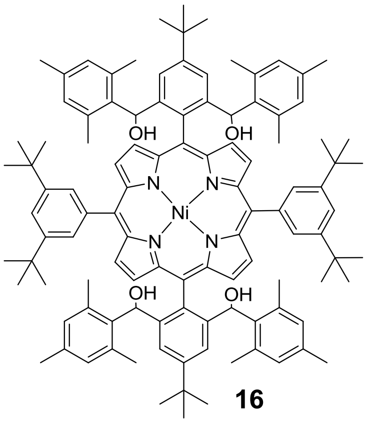

16

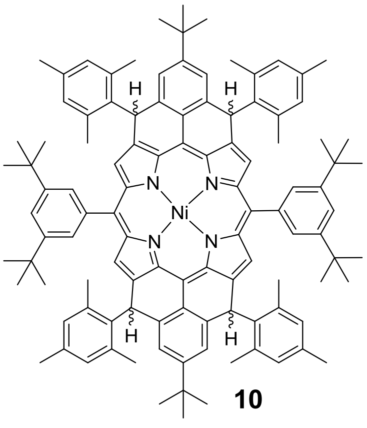

10

Compound **15** (400 mg, 0.36 mmol) was dissolved in THF (30 mL) under argon, and 2-mesitylmagnesium bromide solution (10 mL, 10 mmol, 1.0 M in diethyl ether) was added. The solution was stirred at room temperature for 36 h and then poured into ice water with vigorous stirring. The mixture was extracted by DCM (100 mL) and the organic layer was washed by water, dried over Na_2SO_4. The solvent was removed under vacuum and the crude product was washed by DCM/MeOH (100/1) to afforded compound **16** (tetraol) as a red solid (370 mg, 65% yield), which was used for the

next step directly. HR-MS (APCI): *m/z* =1598.9018, calcd. for $C_{108}H_{124}N_4NiO_4$: *m/z* = 1598.8971, error = -2.9 ppm. Boron trifluoride diethyl etherate (3 mL) was added to a solution of compound **16** (370 mg, 0.23 mol) in DCM (20 mL) and the red solution turned green immediately. After 10 min, methanol (10 mL) and water (20 mL) were added to quench the reaction. The organic layer was separated and dried over Na_2SO_4. The solvent was removed under vacuum and the residue was purified by column chromatography (silica gel, hexane/DCM = 3/1) to afford the desired product **10** in 59% yield (320 mg) for two steps. ^{1}H NMR ($CDCl_3$, 500 MHz): δ 8.50 (s, 4H), 8.11 (br, 2H), 7.73 (br, 2H), 7.60 (s, 2H), 7.30 (s, 4H), 7.12 (s, 4H), 6.78 (s, 4H), 6.75 (s, 4H), 2.89 (s,12H), 2.36 (s, 12H), 1.55 (s, 12H), 1.30-1.27 (m, 36H), 1.23 (s, 18H); ^{13}C NMR ($CDCl_3$, 125 MHz): δ 150.53, 148.78, 142.65, 141.72, 140.22, 139.53, 138.46, 137.91, 137.22, 136.76, 135.88, 131.07, 128.47, 128.10, 126.48, 123.22, 121.15, 120.6, 109.18, 42.34, 34.54, 31.21, 29.70, 22.69, 21.64, 20.97, 20.11. HR-MS (APCI): *m/z* = 1527.8586, calcd. for $C_{108}H_{117}N_4Ni$ (M+1): *m/z* = 1527.8626, error = -2.6 ppm.

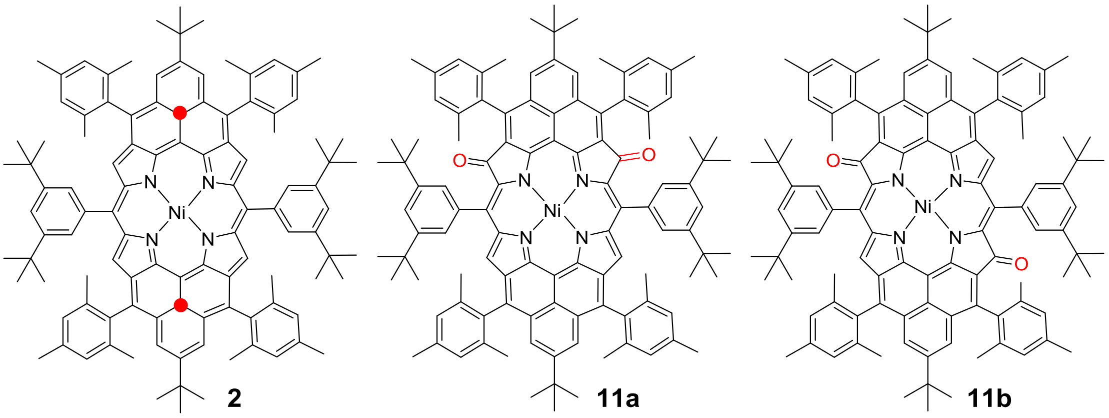

2 **11a** **11b**

Compound **10** (100 mg, 0.065 mmol) was dissolved in 20 mL of CH_2Cl_2 under argon, *p*-chloranil (37.22 mg, 0.13 mmol) was added and the mixture was stirred at room temperature for 10 min. A small amount of this sample can be taken out by syringe for ESR measurement under argon. The mixture was allowed to stir in the air for 3.5 h, and the color of the solution tuned to purple slowly. The solvent was removed under vacuum and the the residue was purified by column chromatography (silica gel, hexane/DCM = 5/1) to afford compounds **11a** (20.3 mg, 20% yield) and **11b** (30.5 mg, 30% yield). Compound **11a**: ^{1}H NMR ($CDCl_3$, 500 MHz): δ 8.20 (s, 2H), 7.65 (s, 2H), 7.29 (s, 2H), 7.15 (s, 4H), 6.97 (s, 4H), 6.93 (s, 4H), 6.28 (s, 2H), 2.38 (s, 6H), 2.35 (s, 6H), 1.92 (s, 12H), 1.88 (s, 12H), 1.55 (s, 18H), 1.26 (s, 36H); ^{13}C NMR ($CDCl_3$, 125 MHz): δ 186.37, 153.92, 150.58, 150.22, 148.80, 148.31, 148.23, 146.35, 143.50, 138.74, 138.50, 137.85, 137.34, 136.90, 135.09, 134.33, 133.65, 132.54, 131.20, 129.86, 129.21, 129.11,

128.70, 127.59, 126.55, 126.45, 121.63, 115.63, 114.69, 107.67, 107.58, 35.81, 35.66, 35.42, 32.01, 31.92, 31.55, 25.84. HR-MS (APCI): *m/z* =1553.8089, calcd. for $C_{108}H_{111}N_4NiO_2$ (M+1): *m/z* = 1553.8055, error = -2.2 ppm. Compound **11b**: H NMR ($CDCl_3$, 500 MHz): δ 7.93 (s, 2H), 7.81 (s, 2H), 7.29 (s, 2H), 7.15 (s, 4H), 6.96 (s, 4H), 6.94 (s, 4H), 6.25 (s, 2H), 2.37 (s, 6H), 2.36 (s, 6H), 2.00 (s, 12H), 1.86 (s, 12H), 1.56 (s, 36H), 1.11 (s, 18H); ^{13}C NMR ($CDCl_3$, 125 MHz): δ 186.49, 156.78, 150.57, 149.86, 148.45, 147.33, 144.45, 142.66, 138.74, 138.51, 137.55, 137.20, 136.54, 134.63, 134.25, 132.60, 131.69, 131.39, 129.34, 129.05, 128.19, 127.65, 127.40, 126.42, 123.41, 121.67, 115.01, 107.29, 35.67, 35.61, 32.00, 31.37, 21.54, 21.47, 20.77, 20.62, 20.33, 20.17, 20.14. HR-MS (APCI): *m/z* =1553.8108, calcd. for $C_{108}H_{111}N_4NiO_2$ (M+1): *m/z* = 1553.8055, error = -3.4 ppm. Compound **2**, HR-MS (APCI): m/z =1522.8208, calcd. for $C_{108}H_{112}N_4Ni$ (M+1): *m/z* = 1522.8235, error = 1.8 ppm.

2. Absorption and ESR spectra of triplet diradical 2

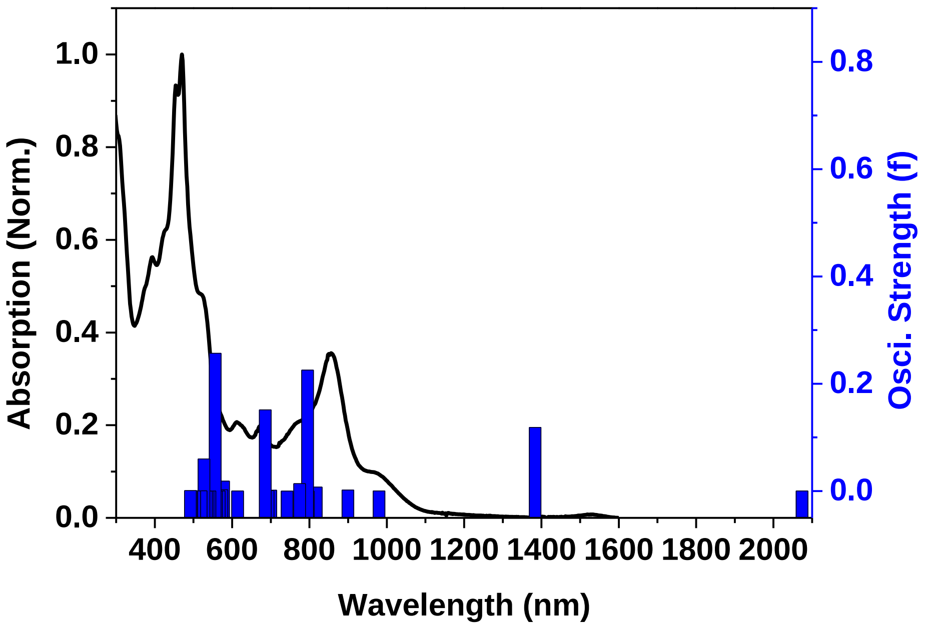

Fig. S1. UV-Vis-NIR absorption spectrum of the triplet diradical **2** generated *in situ* in CH_2Cl_2 along with the simulated excitation transitions obtained by UB3LYP/6-31G* level calculations (*vide infra*).

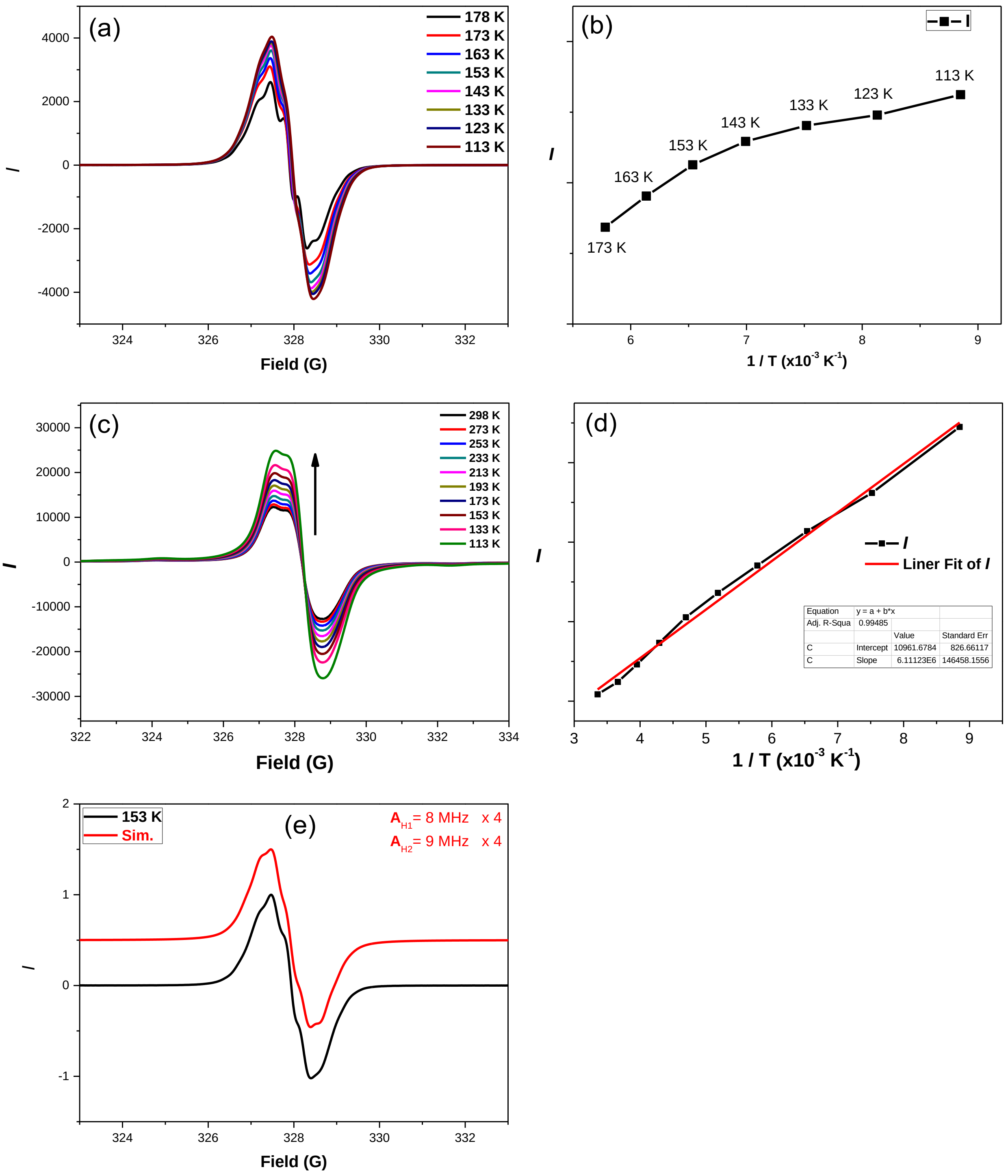

Fig. S2. ESR spectra (a) of *the in situ* generated triplet briradical **2** in the frozen CH_2Cl_2 solution and the integrated ESR intensity *I* -1/*T* curve (b); VT ESR spectra (c) of **2** in solid powder form and the *I* -1/*T* curve (d); ESR spectrum of the frozen solution recorded at 153 K and simulated ESR spectrum (e).

3. VT NMR spectrum of compound 1

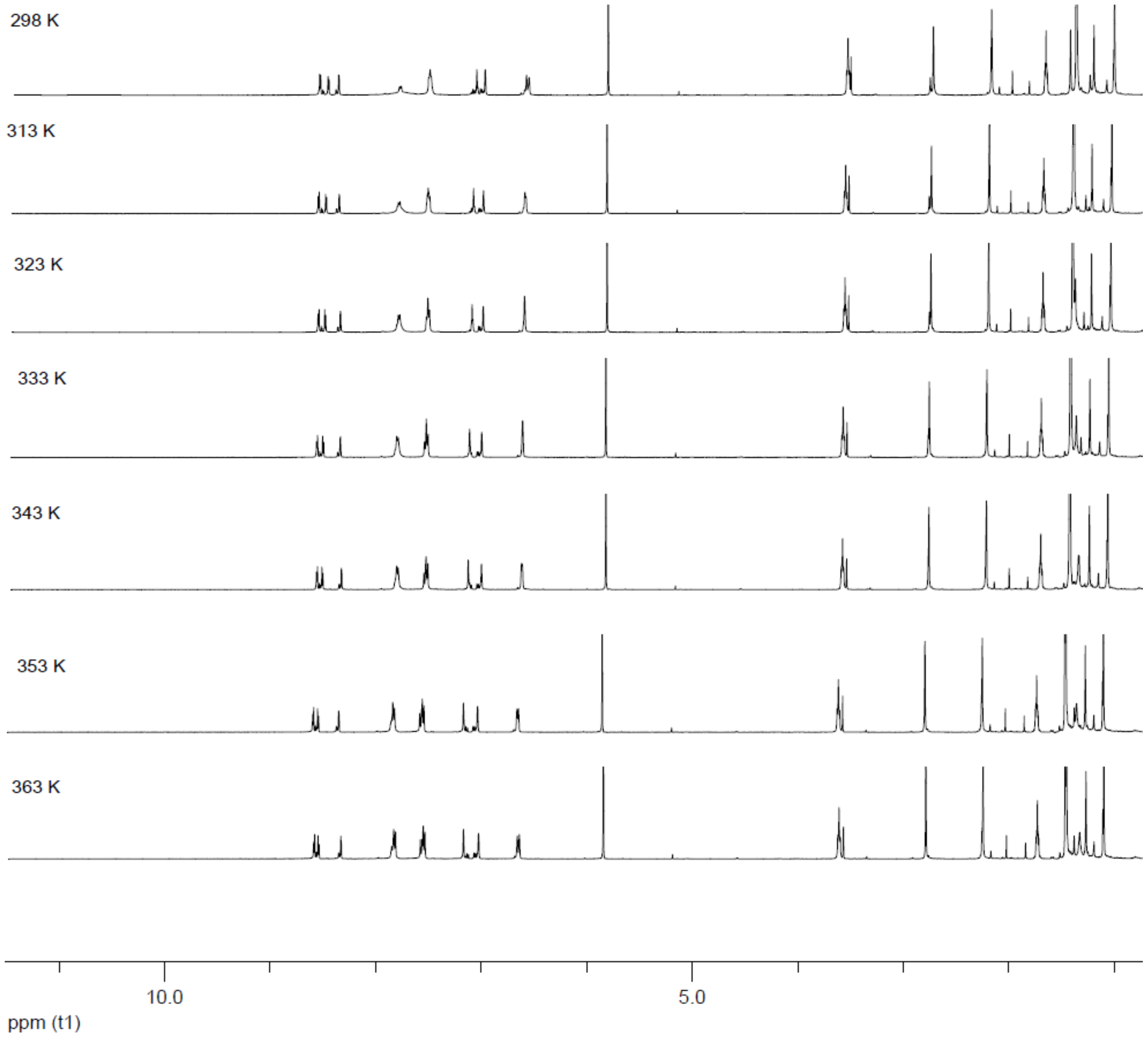

Fig. S3. VT ^{1}H NMR spectrum of compound **1** (500 MHz, $C_2D_2Cl_4$, from 298K to 363K).

4. DFT calculations

Theoretical calculations were performed with the *Gaussian09* program suite using a supercomputer.[1] All calculations were carried out using the density functional theory (DFT) method with Becke's three-parameter hybrid exchange functionals and the Lee-Yang-Parr correlation functional (B3LYP) employing the 6-31G(d,p) basis set for all atoms.[2] The ground-state geometry and electronic structure of **2** was calculated using a UB3LYP/6-31G* method and **2** turned out to be a triplet biradical. The geometries of **1**, **11a** and **11b** were fully optimized in gas phase using the default convergence criteria without any constraints and confirmed by frequency calculations. Their ground-state absorption spectra were simulated by the time-dependent (TD) DFT calculations at UB3LYP/6-31G* level.

Table S1. Calculated excitation transitions for **1**.

Calcd. (nm)	*f*	Composition (H=HOMO, L= LUMO, L+1 = LUMO+1, etc.)
1369.07395	0.0483	HOMO->LUMO (99%)
608.149	0.1216	HOMO->L+1 (83%)
462.10711	0.4473	H-2->LUMO (10%), H-2->L+1 (14%), HOMO->L+2 (63%)
415.9119	0.2229	H-10->LUMO (25%), H-6->LUMO (14%), H-1->L+1 (44%)
388.83315	0.3257	H-13->LUMO (87%)
382.96011	0.1799	H-2->L+1 (53%), H-1->L+2 (21%)

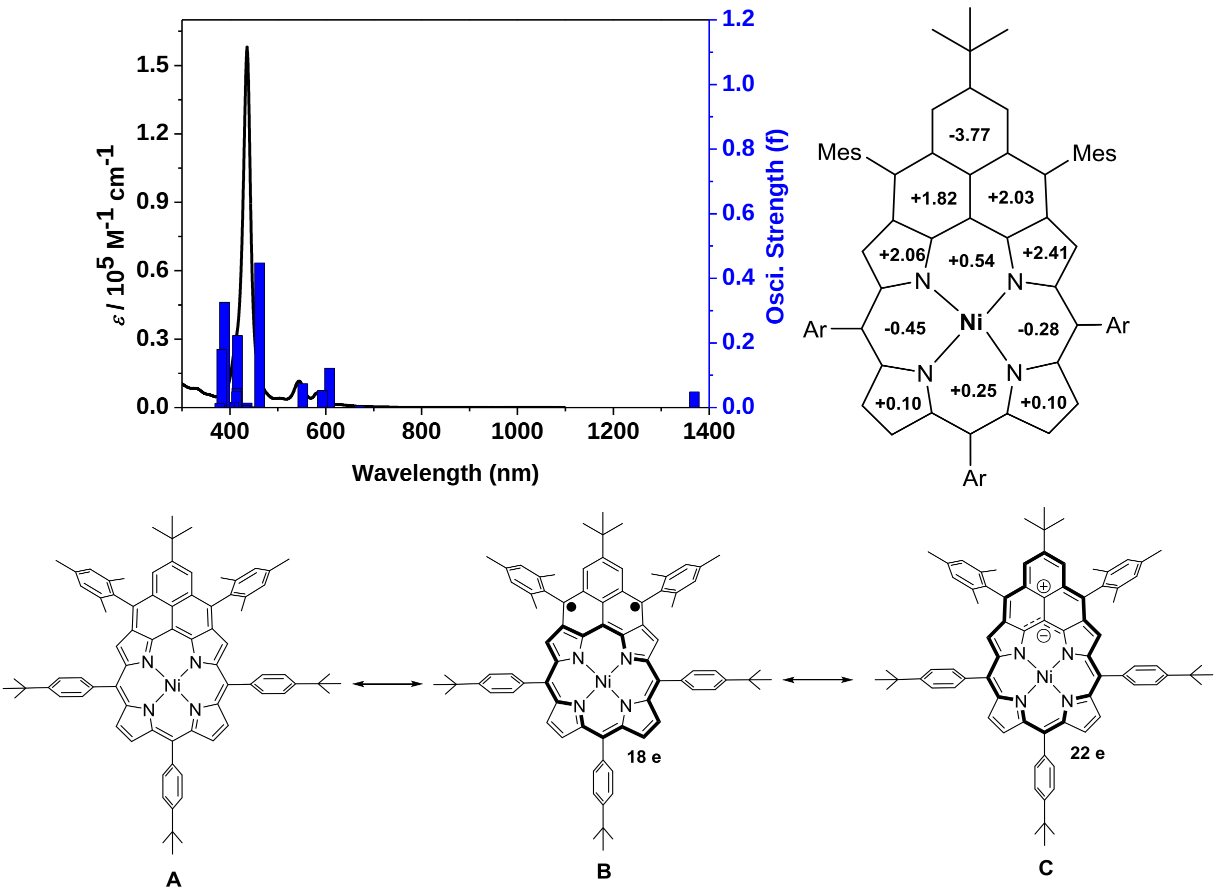

Fig. S4. Calculated absorption spectrum (left), NICS (0) values (right) and three possible resonance forms (bottom) for compound **1**.

Table S2. Calculated excitation transitions for the triplet biradical **2**.

Calcd. (nm)	*f*.	Composition (H=HOMO, L= LUMO, A = α, and

		B = β)
2073.99	2E-4	HOMO(A)->LUMO(A) (96%)
1383.59	0.1187	H-1(A)->LUMO(A) (82%), HOMO(B)->L+1(B) (13%)
794.92	0.2253	H-1(A)->LUMO(A) (11%), HOMO(B)->L+1(B) (81%)
774.60	0.0137	HOMO(A)->L+1(A) (76%), HOMO(B)->L+2(B) (20%)
685.56	0.1515	H-2(A)->LUMO(A) (33%), HOMO(A)->L+1(A) (12%), HOMO(B)->L+2(B) (48%)
577.88	0.0186	H-1(B)->LUMO(B) (80%)
556.15	0.2567	H-7(A)->LUMO(A) (11%), H-2(B)->LUMO(B) (80%)

Table S3. Calculated excitation transitions for **11a**

Calcd. (nm)	*f.*	Composition (H=HOMO, L= LUMO, L+1 = LUMO+1, etc.)
925.1107	0.0364	HOMO->LUMO (98%)
754.89124	0.0326	HOMO->L+1 (84%)
561.77316	0.5339	H-1->LUMO (46%), HOMO->L+2 (44%)
551.65	0.102	H-1->L+1 (93%)
472.85788	0.0406	H-5->LUMO (52%), H-3->LUMO (12%), HOMO->L+3 (10%)
465.85758	0.0657	H-6->LUMO (17%), H-2->L+1 (22%), H-1->L+2 (13%), HOMO->L+3 (30%)

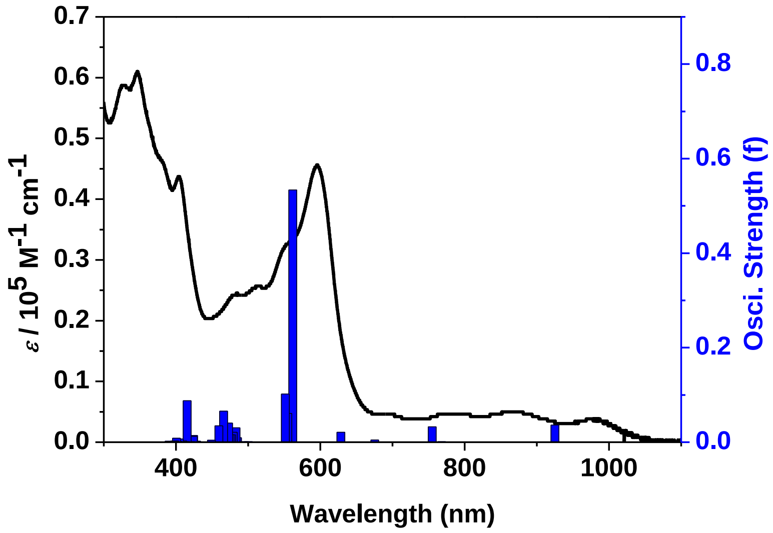

Fig. S5. Calculated excitation transitions for **11a** together with its absorption spectrum in CH_2Cl_2.

Table S4. Calculated excitation transitions for **11b**

Calcd. (nm)	*f.*	Composition (H=HOMO, L= LUMO, L+1 = LUMO+1, etc.)
778.05671	0.2301	H-20->L+4 (11%), HOMO->LUMO (66%), HOMO->L+4 (14%)
757.56652	0.1125	H-20->L+4 (24%), HOMO->LUMO (29%), HOMO->L+4 (31%)
485.8662	0.2922	H-4->LUMO (22%), H-3->LUMO (67%)
480.31355	0.1108	H-4->LUMO (57%), H-3->LUMO (19%)
463.10823	0.0467	H-7->L+1 (18%), H-5->L+1 (21%), H-2->L+1 (48%)

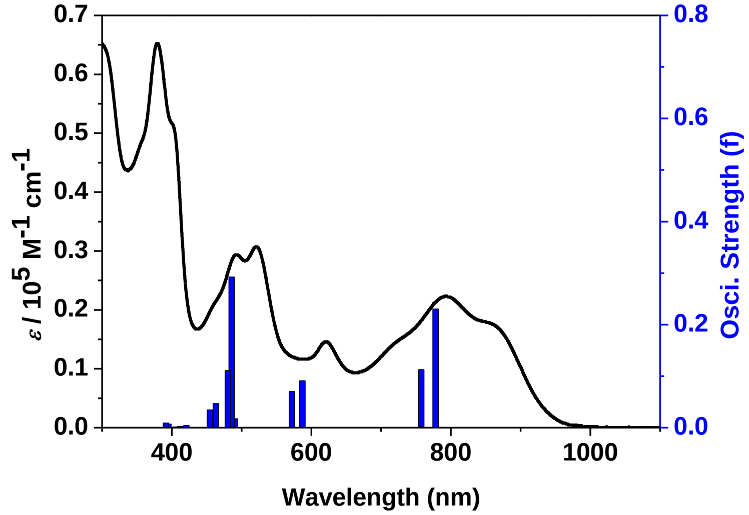

Fig. S6. Calculated excitation transitions for **11b** together with its absorption spectrum in CH_2Cl_2.

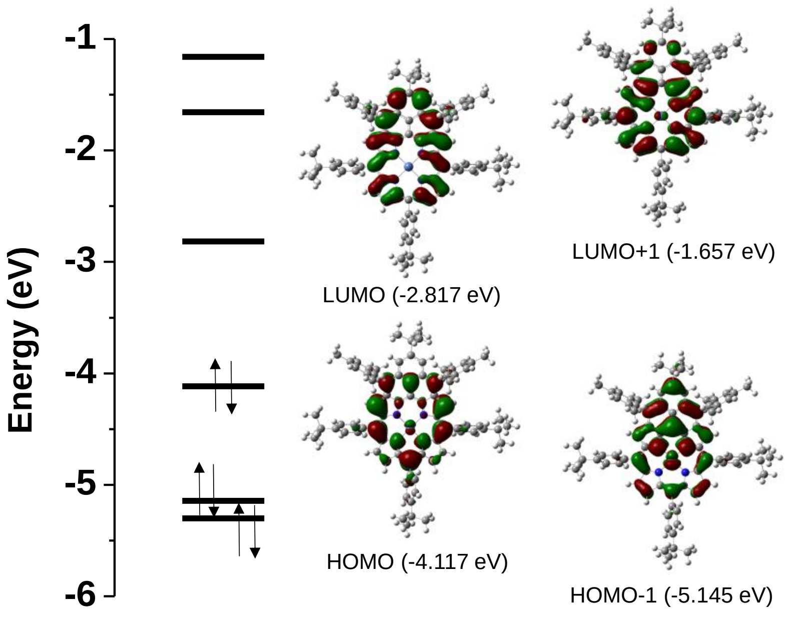

Fig. S7. Calculated frontier molecular orbital profiles and energy diagram of **1** (hydrogen atoms are omitted for clarity).

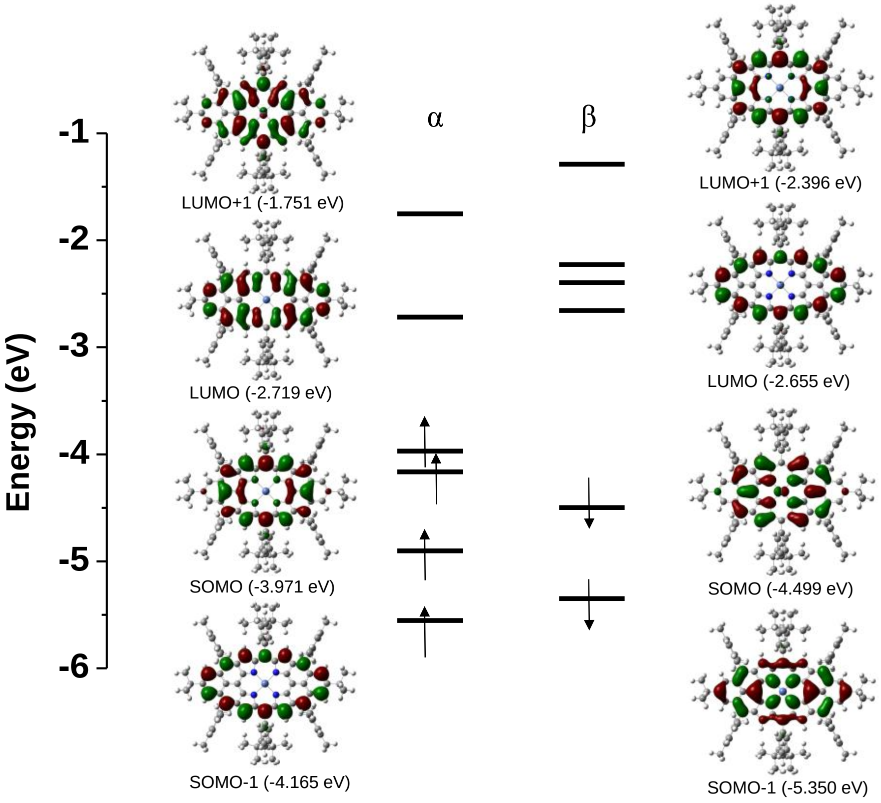

Fig. S8. Calculated frontier molecular orbital profiles and energy diagram of the triplet diradical **2** (hydrogen atoms are omitted for clarity).

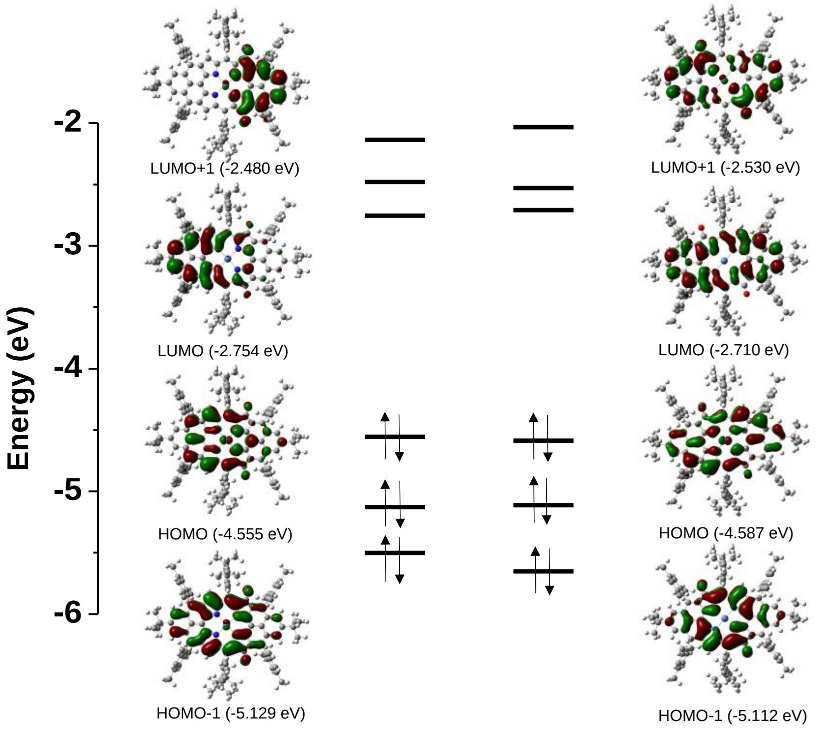

Fig. S9. Calculated frontier molecular orbital profiles and energy diagram of **11a** (left) and **11b** (right) (hydrogen atoms are omitted for clarity).

References:

(1) Gaussian 09, Revision A.1, Frisch, M. J.; Trucks, G. W.; Schlegel, H. B.; Scuseria, G. E.; Robb, M. A.; Cheeseman, J. R.; Scalmani, G.; Barone, V.; Mennucci, B.; Petersson, G. A.; Nakatsuji, H.; Caricato, M.; Li, X.; Hratchian, H. P.; Izmaylov, A. F.; Bloino, J.; Zheng, G.; Sonnenberg, J. L.; Hada, M.; Ehara, M.; Toyota, K.; Fukuda, R.; Hasegawa, J.; Ishida, M.; Nakajima, T.; Honda, Y.; Kitao, O.; Nakai, H.; Vreven, T.; Montgomery, Jr., J. A.; Peralta, J. E.; Ogliaro, F.; Bearpark, M.; Heyd, J. J.; Brothers, E.; Kudin, K. N.; Staroverov, V. N.; Kobayashi, R.; Normand, J.; Raghavachari, K.; Rendell, A.; Burant, J. C.; Iyengar, S. S.; Tomasi, J.; Cossi, M.; Rega, N.; Millam, N. J.; Klene, M.; Knox, J. E.; Cross, J. B.; Bakken, V.; Adamo, C.; Jaramillo, J.; Gomperts, R.; Stratmann, R. E.; Yazyev, O.; Austin, A. J.; Cammi, R.; Pomelli, C.; Ochterski, J. W.; Martin, R. L.; Morokuma, K.; Zakrzewski, V. G.; Voth, G. A.; Salvador, P.; Dannenberg, J. J.; Dapprich, S.; Daniels, A. D.; Farkas, Ö.; Foresman, J. B.; Ortiz, J. V.; Cioslowski, J.; Fox, D. J. Gaussian, Inc., Wallingford CT, **2009**.

(2) (a) Becke, A. D. *Phys. Rev. A* **1988**, *38*, 3098. (b) Lee, C.; Yang, W.; Parr, R. G. *Phys. Rev. B* **1988**, *37*, 785.

5. Transient absorption spectra and Z-scan curves

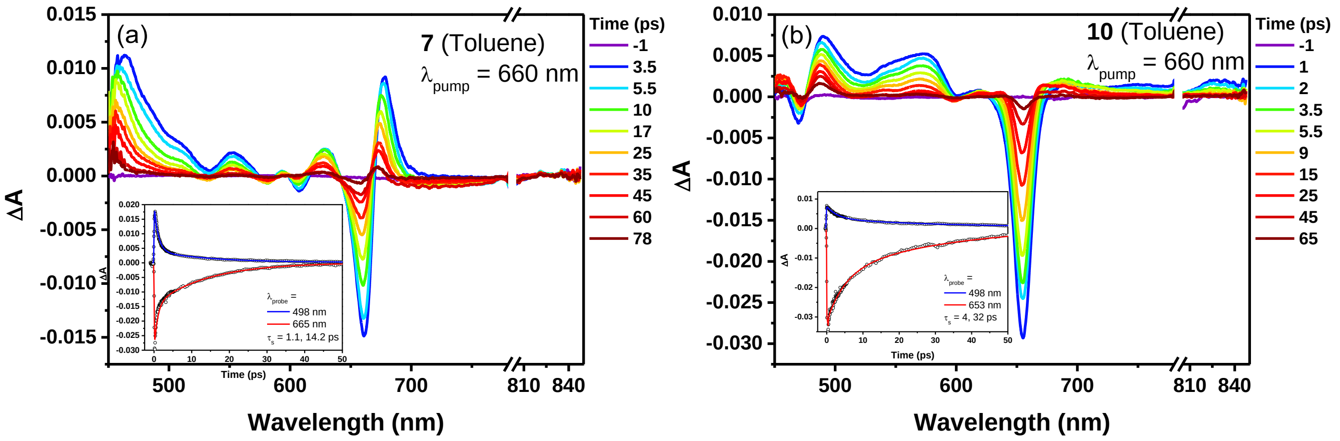

Fig. S10. Femtosecond transient absorption spectra and decay profiles (inset) of **7** (a) and **10** (b) in toluene measured at room temperature (296 K). The excitation wavelength is 660 nm.

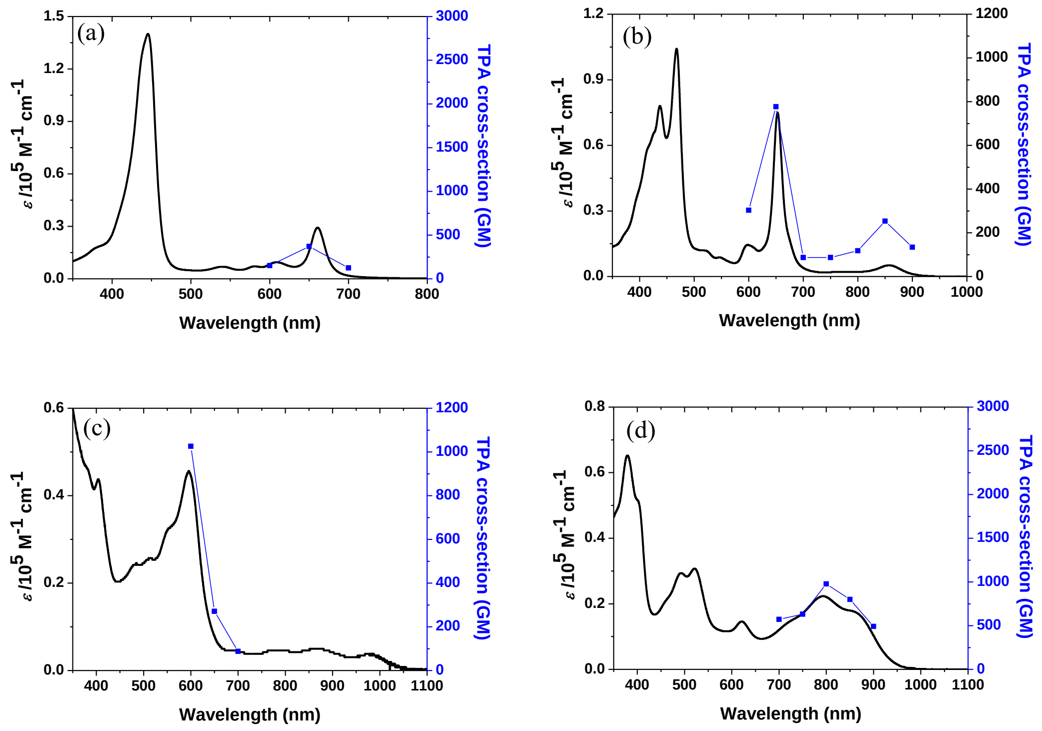

Fig. S11. OPA (black solid line and left vertical axis) and TPA spectra (blue symbols and right vertical axis) of **7** (a), **10** (b), **11a** (c), and **11b** (d). TPA spectra are plotted at $\lambda_{ex}/2$.

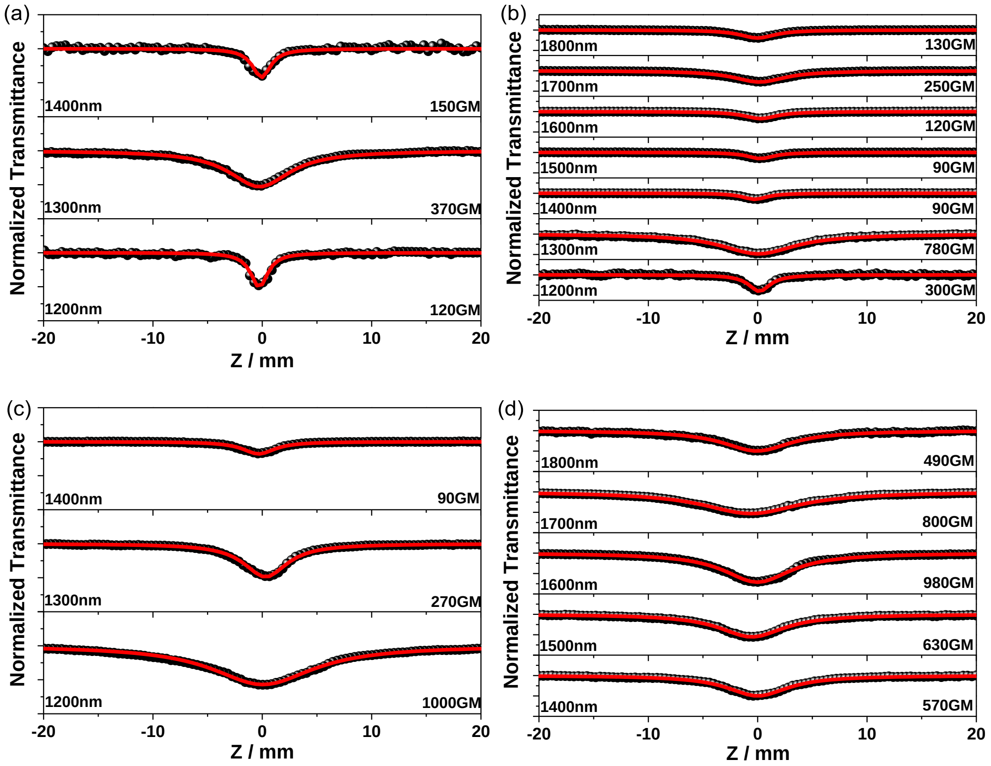

Fig. S12. Z-scan curves of **7** (a), **10** (b), **11a** (c) and **11b** (d) in toluene by photoexcitation in the range from 1200 to 1800 nm.

6. Electrochemical data

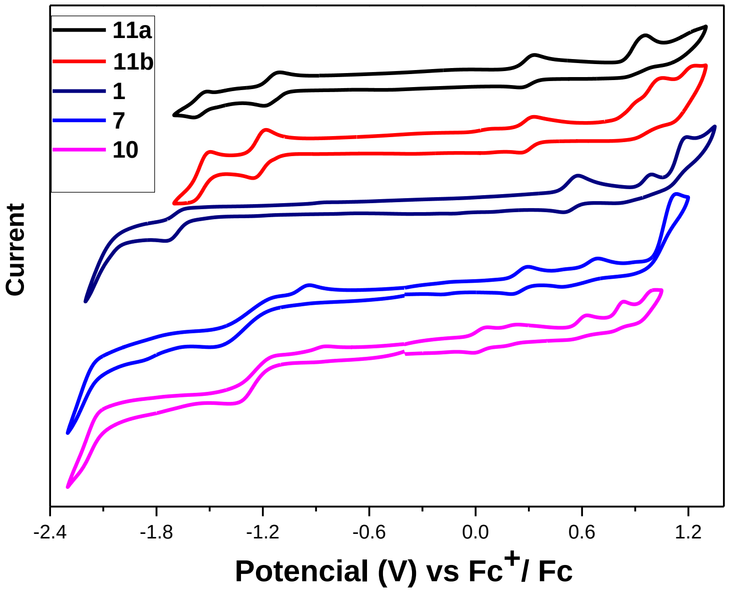

Fig. S13. Cyclic voltammograms of **1**, **7**, **10**, **11a** and **11b** in CH_2Cl_2 with 0.1 M Bu_4NPF_6 as a supporting electrolyte, AgCl/Ag as a reference electrode, a Au disk as a working electrode, a Pt. wire as a counter electrode, and a scan rate of 50 mv s^{-1}.

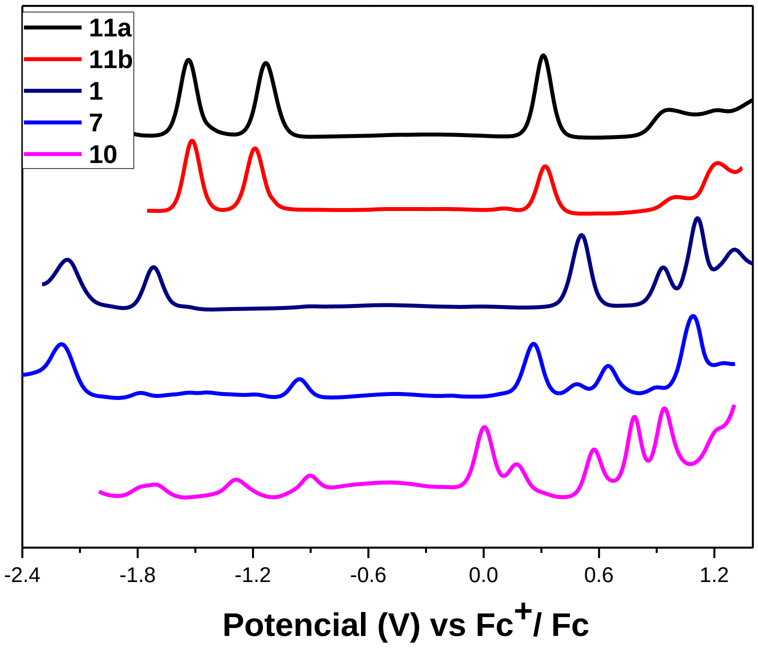

Fig. S14. Differential pulse voltammograms of **1**, **7**, **10**, **11a** and **11b** in CH_2Cl_2 with 0.1M Bu_4NPF_6 as supporting electrolyte, AgCl/Ag as reference electrode, Au disk as working electrode and Pt wire as counter electrode. Fc+/Fc was used as external reference.

7. NMR spectra and HR mass spectra

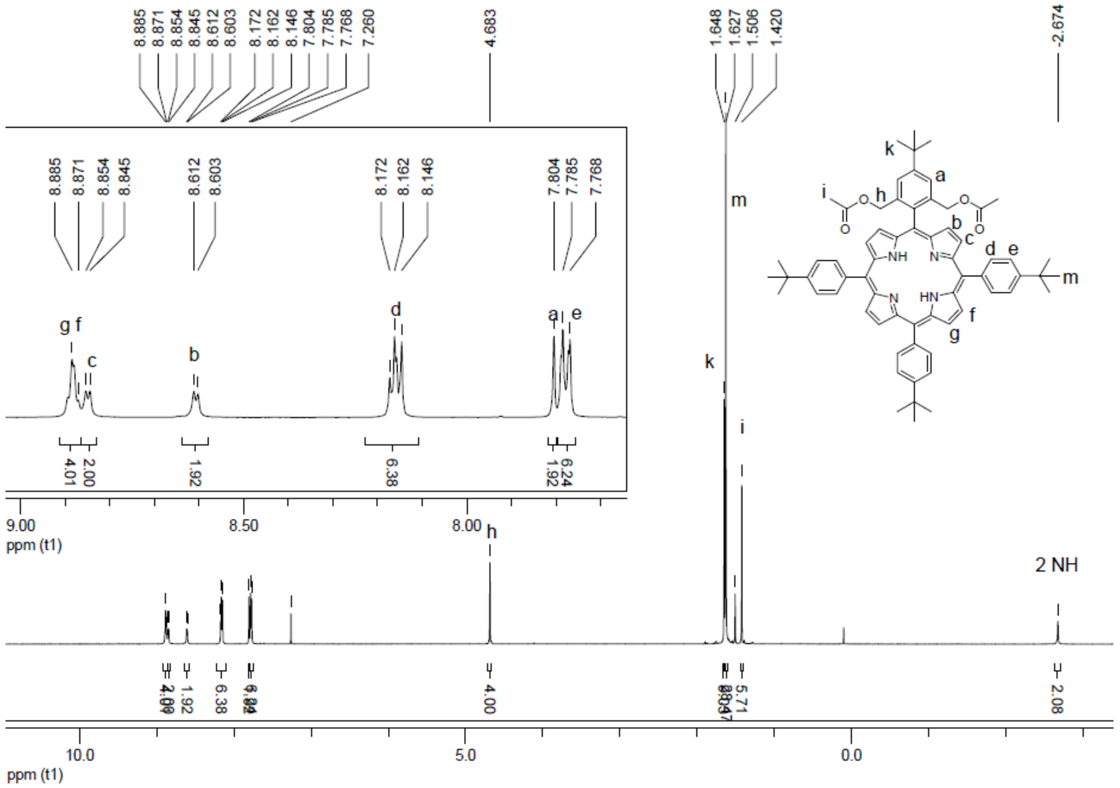

Fig. S15. ^{1}H NMR spectrum of compound **4** (500 MHz, $CDCl_3$, rt).

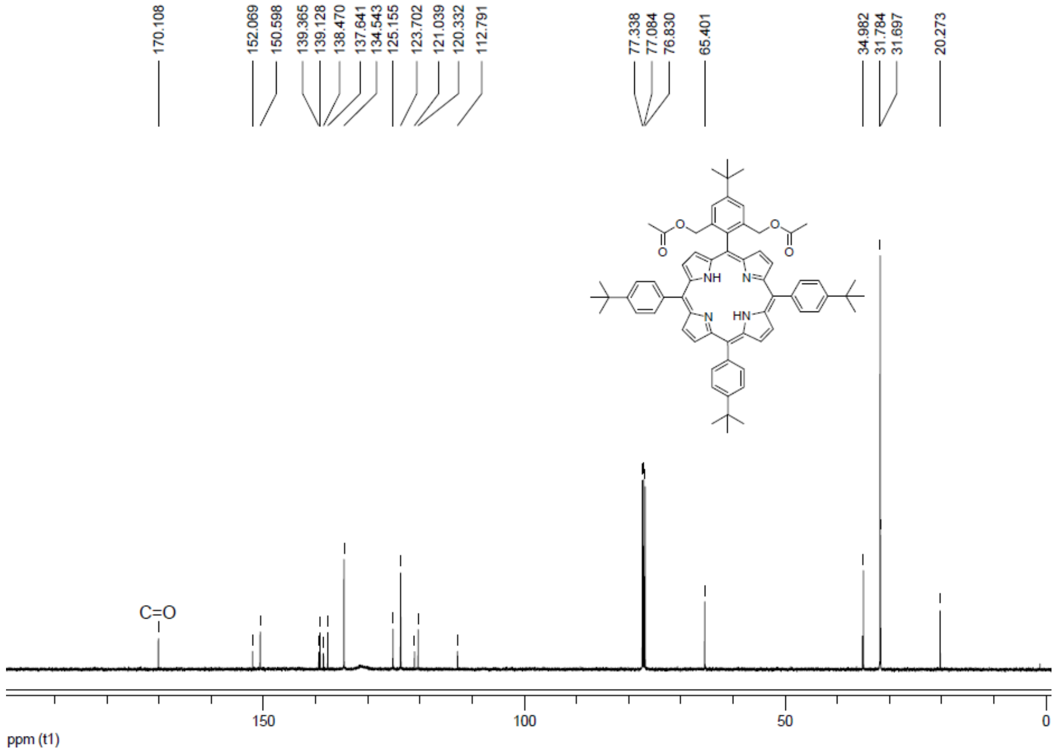

Fig. S16. ^{13}C NMR spectrum of compound **4** (500 MHz, $CDCl_3$, rt).

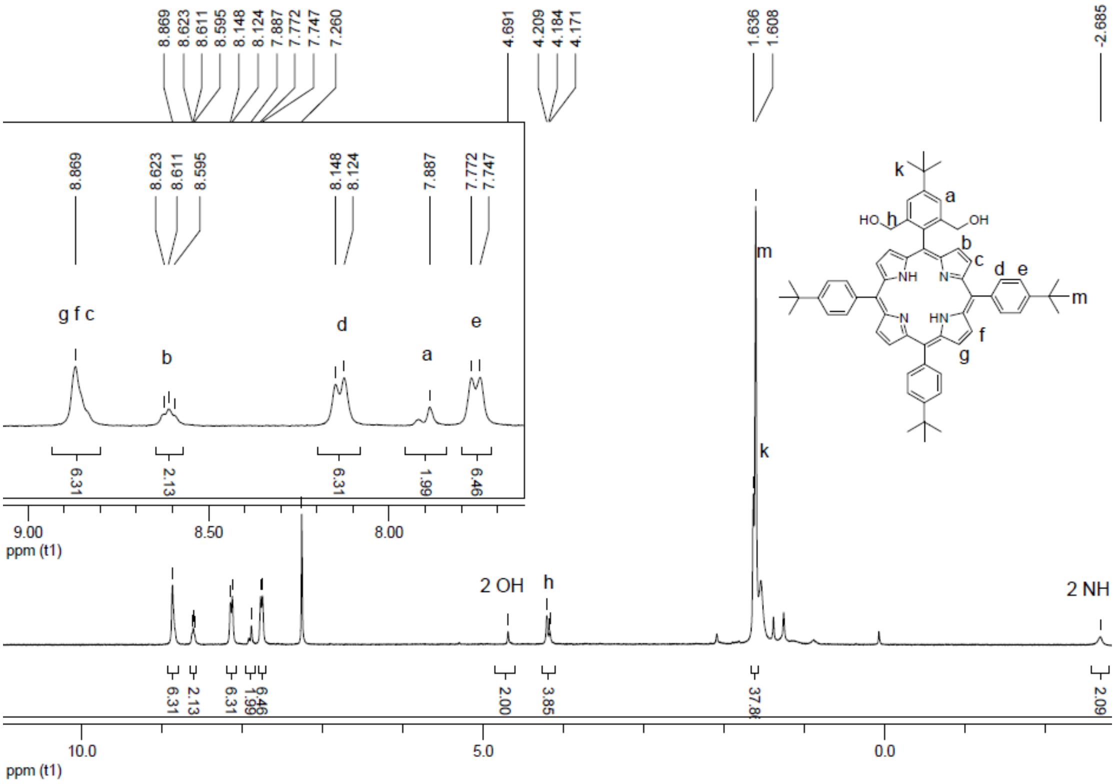

Fig. S17. ^{1}H NMR spectrum of compound **5** (500 MHz, $CDCl_3$, rt).

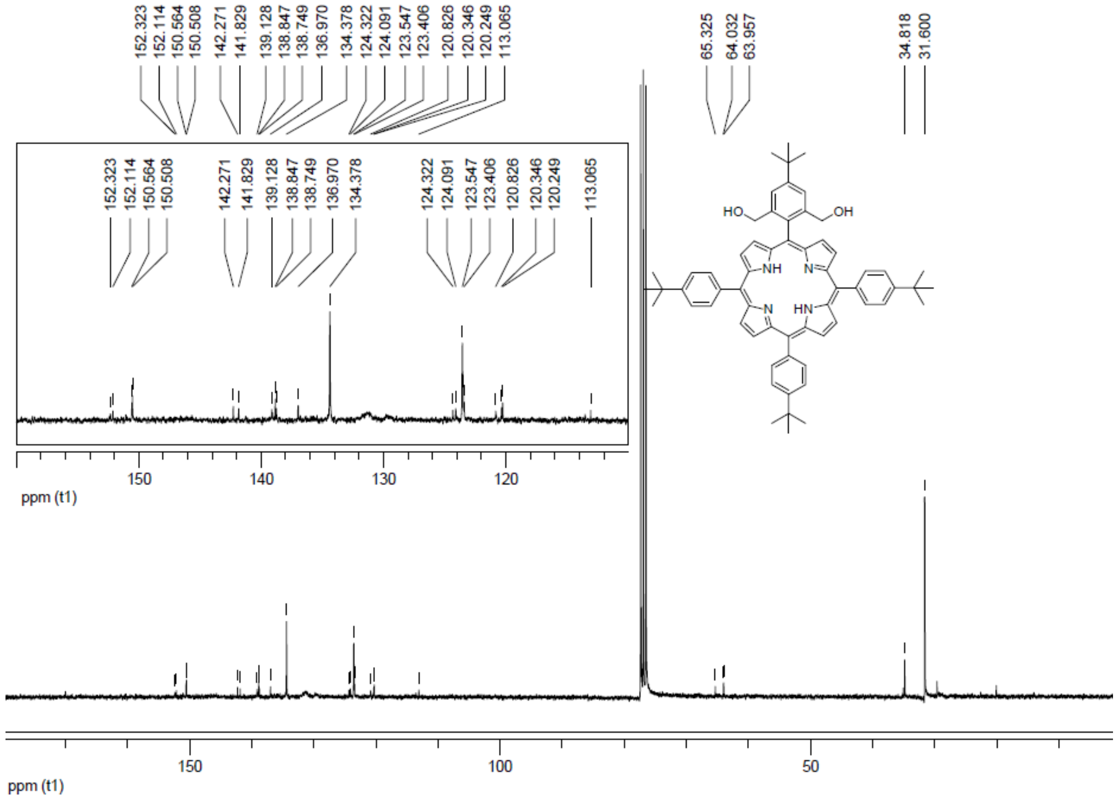

Fig. S18. ^{13}C NMR spectrum of compound **5** (500 MHz, $CDCl_3$, rt).

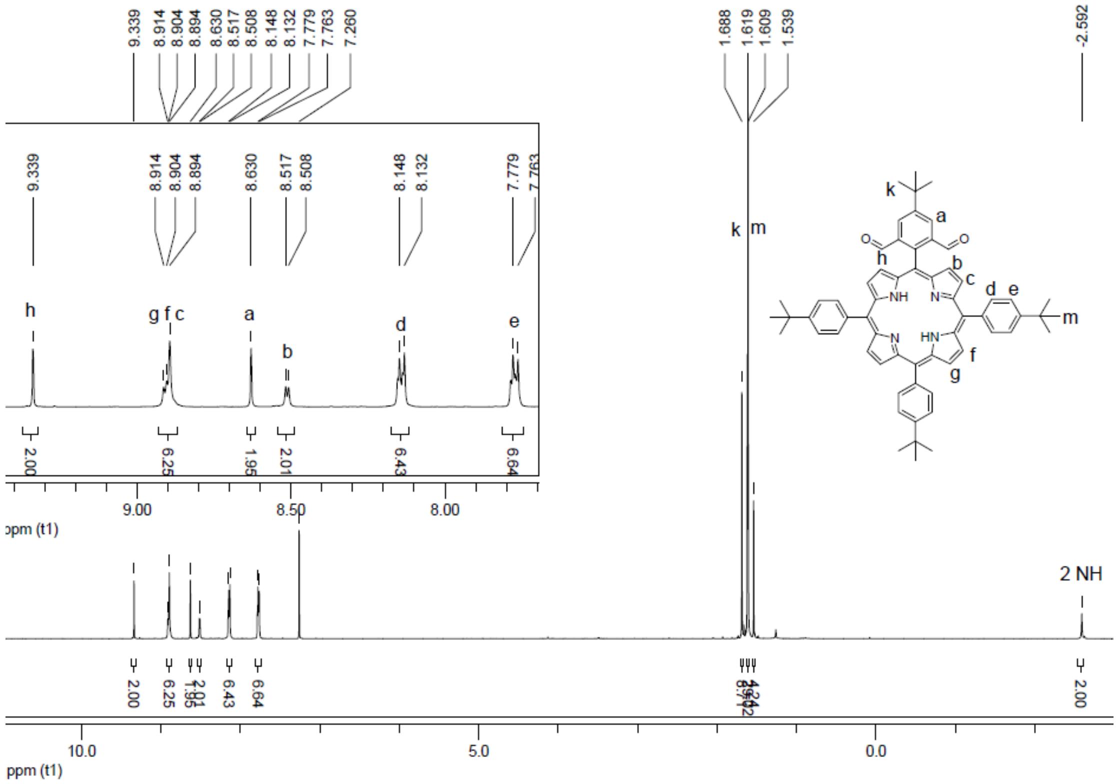

Fig. S19. ^{1}H NMR spectrum of compound **6-H2** (500 MHz, $CDCl_3$, rt).

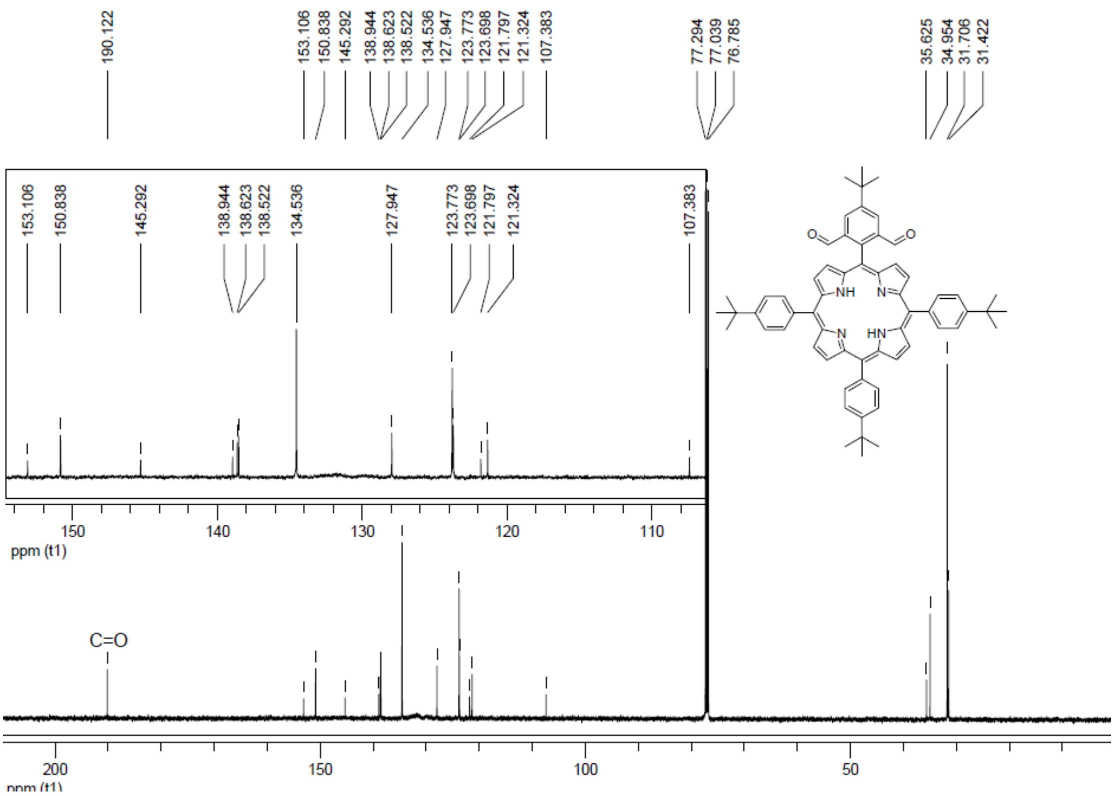

Fig. S20. ^{13}C NMR spectrum of compound **6-H2** (500 MHz, $CDCl_3$, rt).

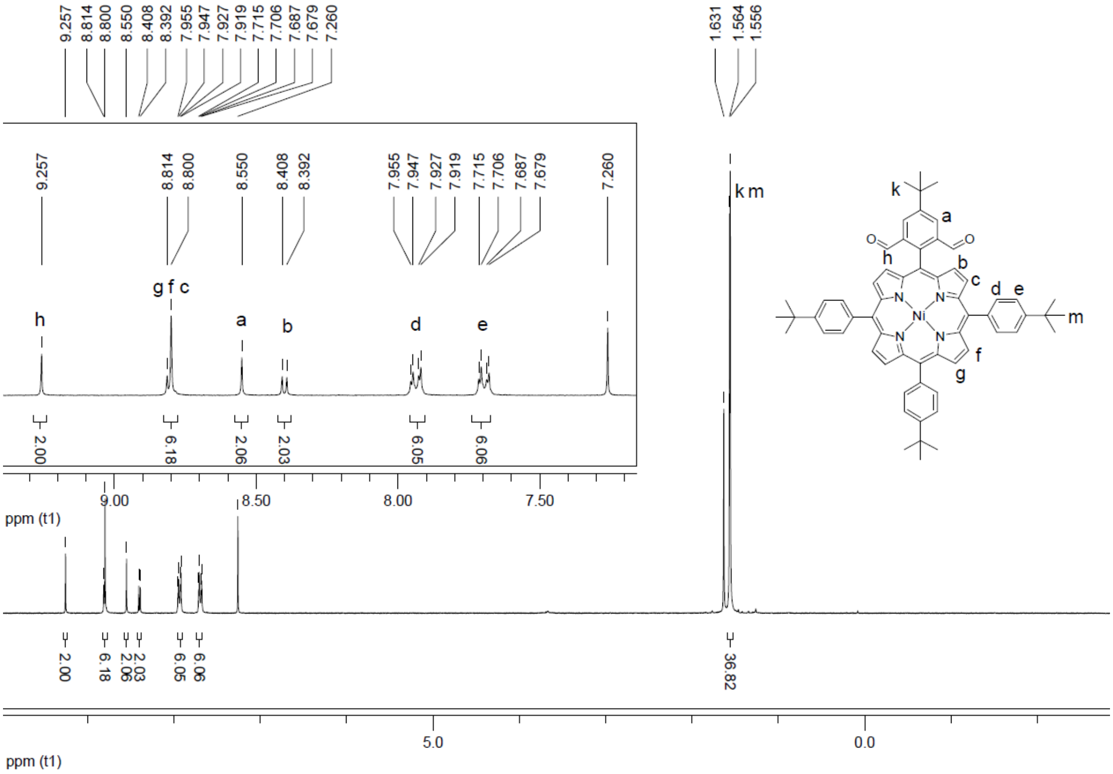

Fig. S21. ^{1}H NMR spectrum of compound **6** (500 MHz, $CDCl_3$, rt).

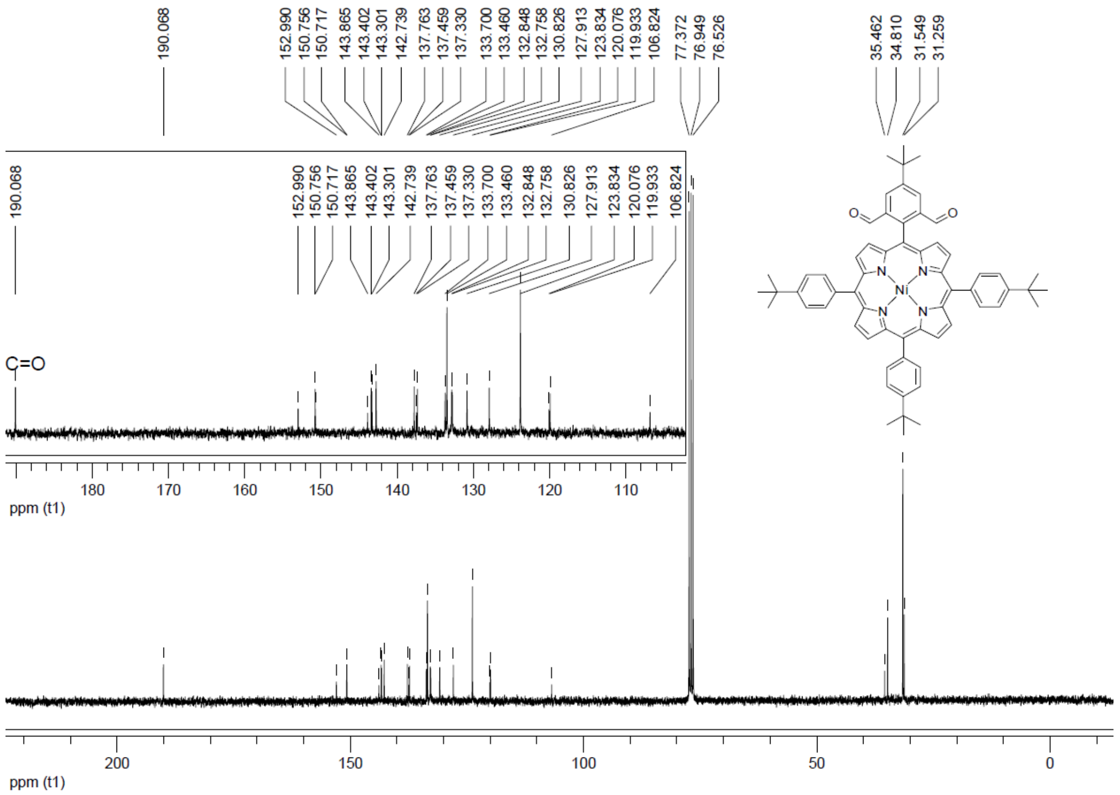

Fig. S22. ^{13}C NMR spectrum of compound **6** (500 MHz, $CDCl_3$, rt).

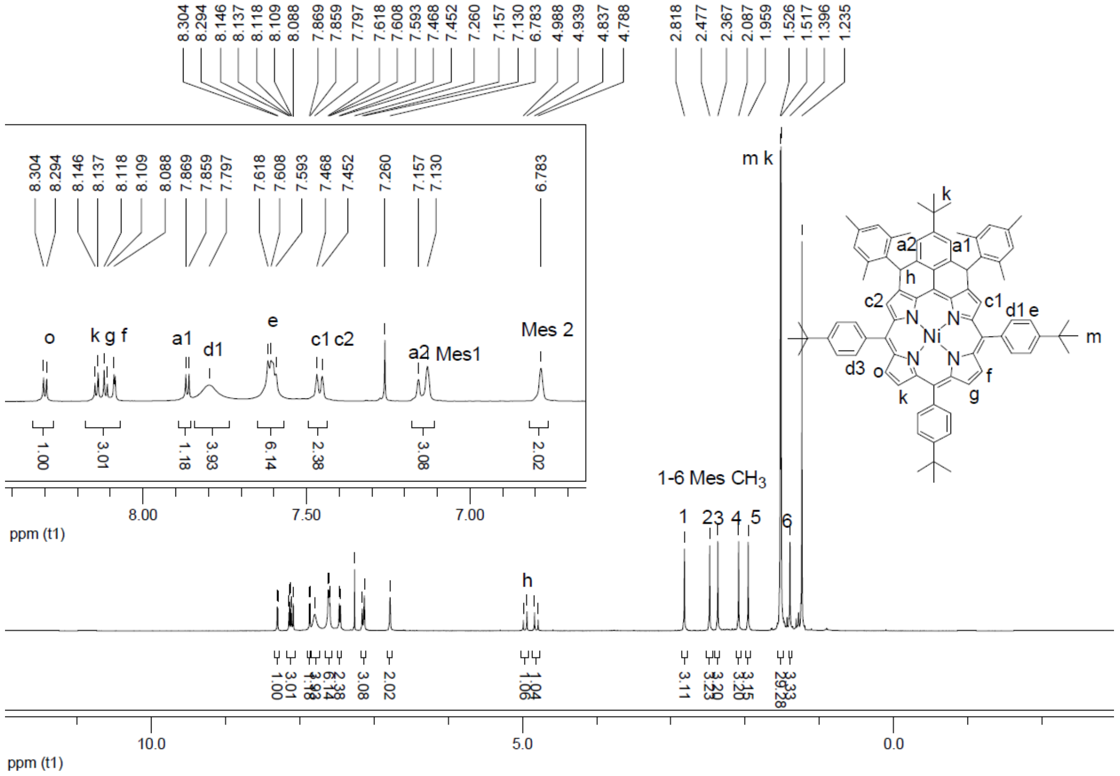

Fig. S23. ^{1}H NMR spectrum of compound **7** (500 MHz, $CDCl_3$, rt).

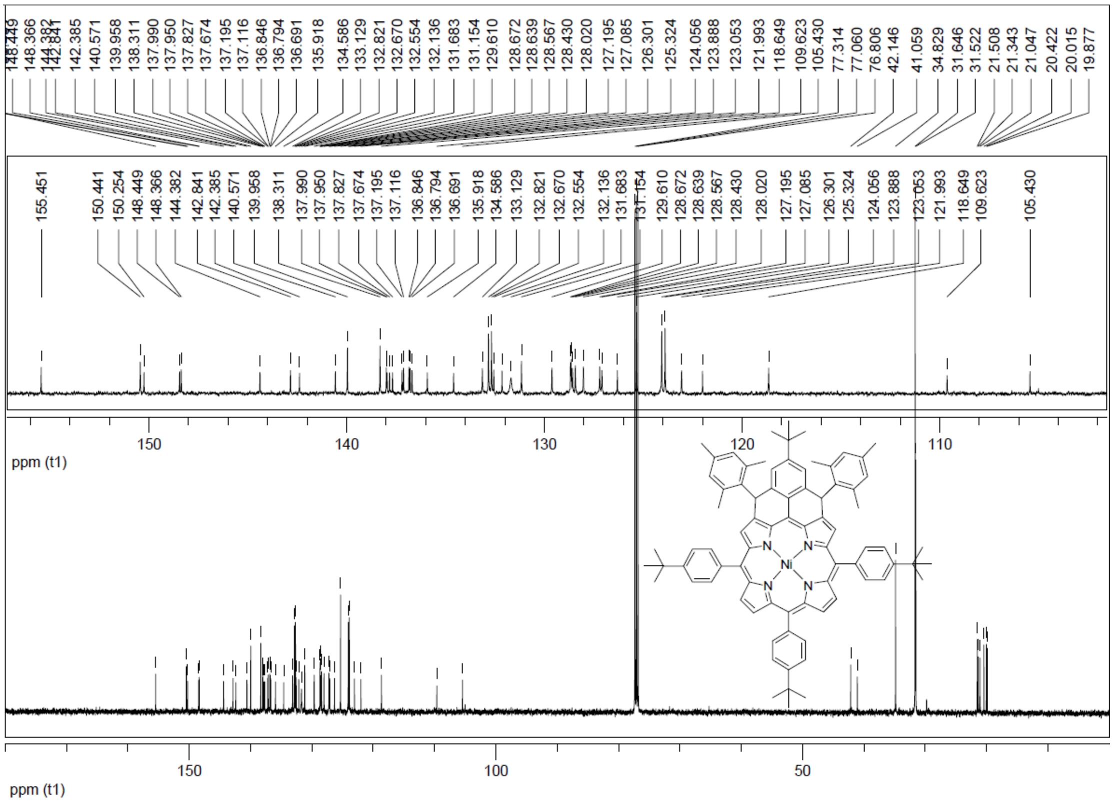

Fig. S24. ^{13}C NMR spectrum of compound **5** (500 MHz, $CDCl_3$, rt).

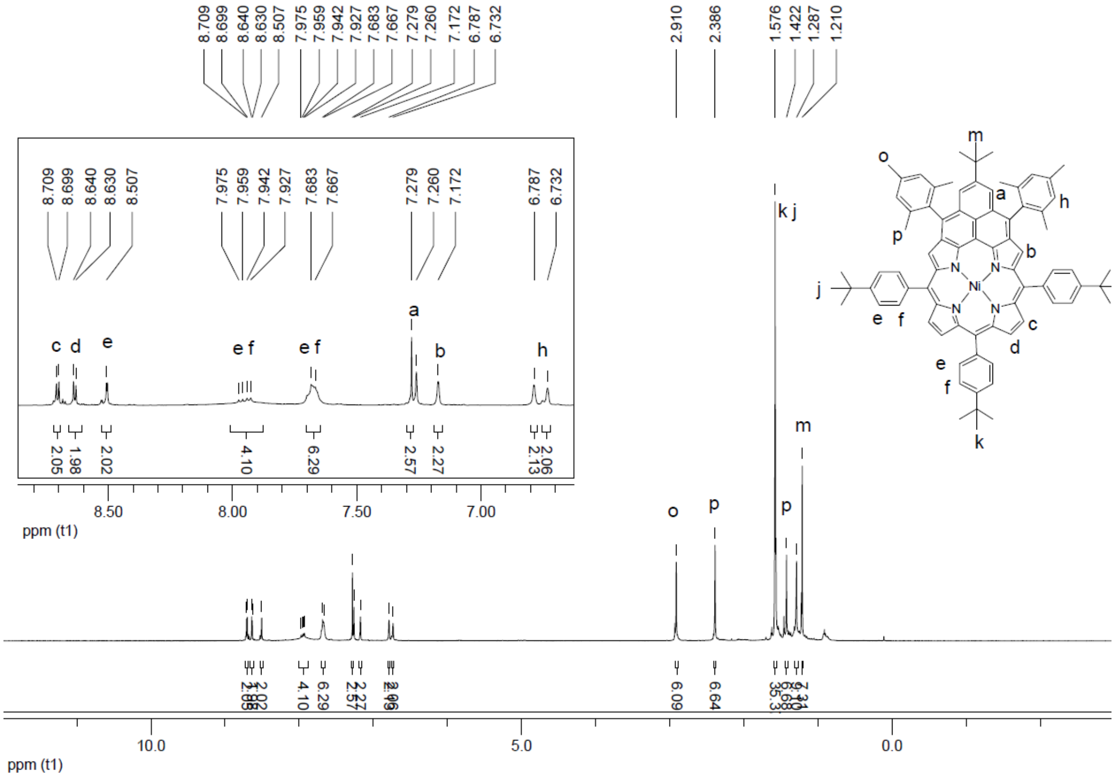

Fig. S25. ^{1}H NMR spectrum of compound **1** (500 MHz, $CDCl_3$, rt).

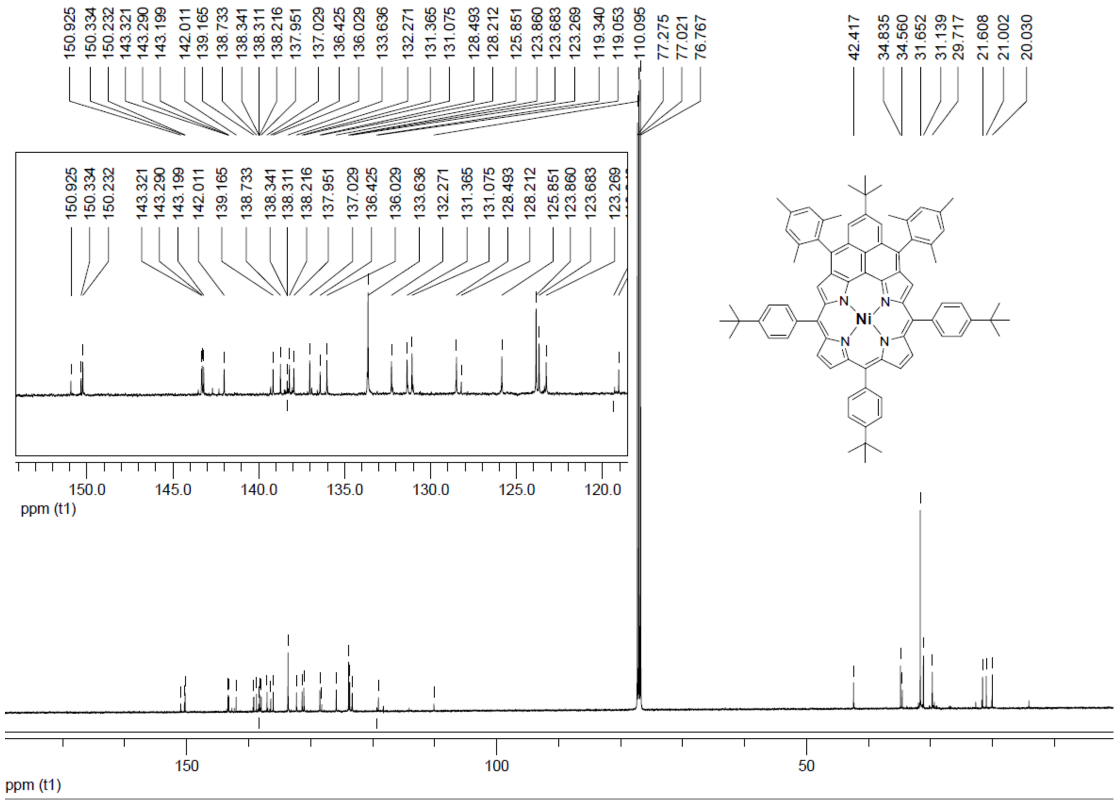

Fig. S26. ^{13}C NMR spectrum of compound **1** (500 MHz, $CDCl_3$, rt).

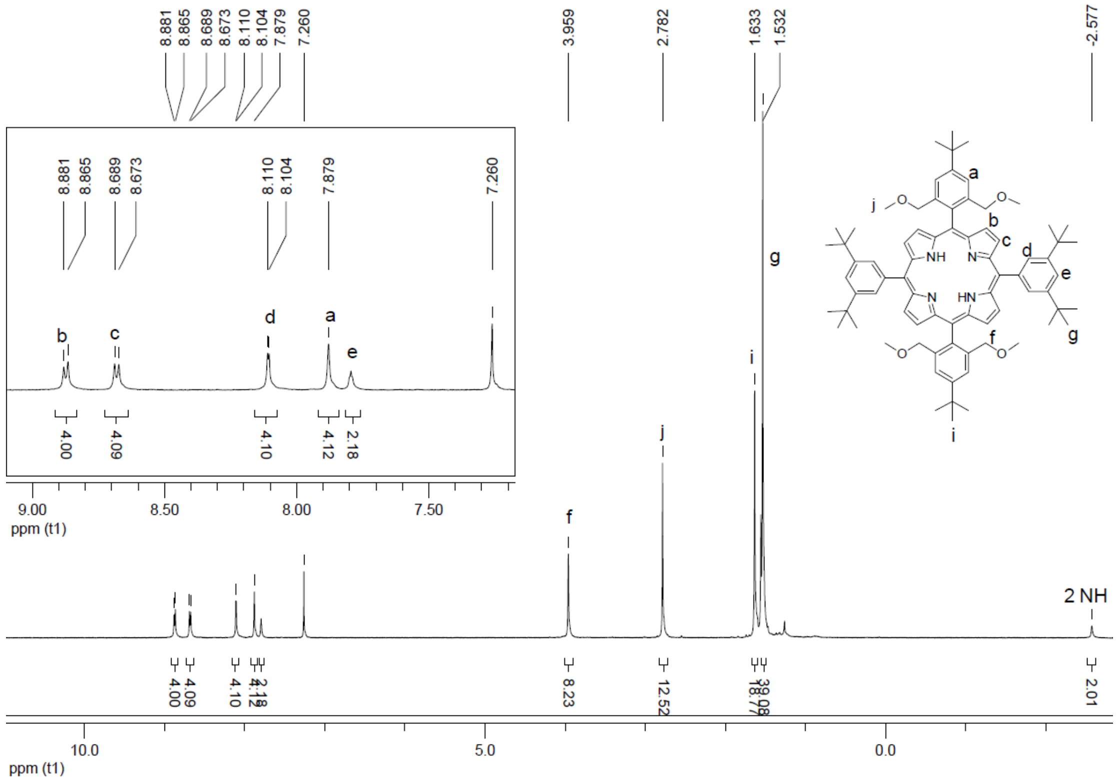

Fig. S27. ^{1}H NMR spectrum of compound **8** (500 MHz, $CDCl_3$, rt).

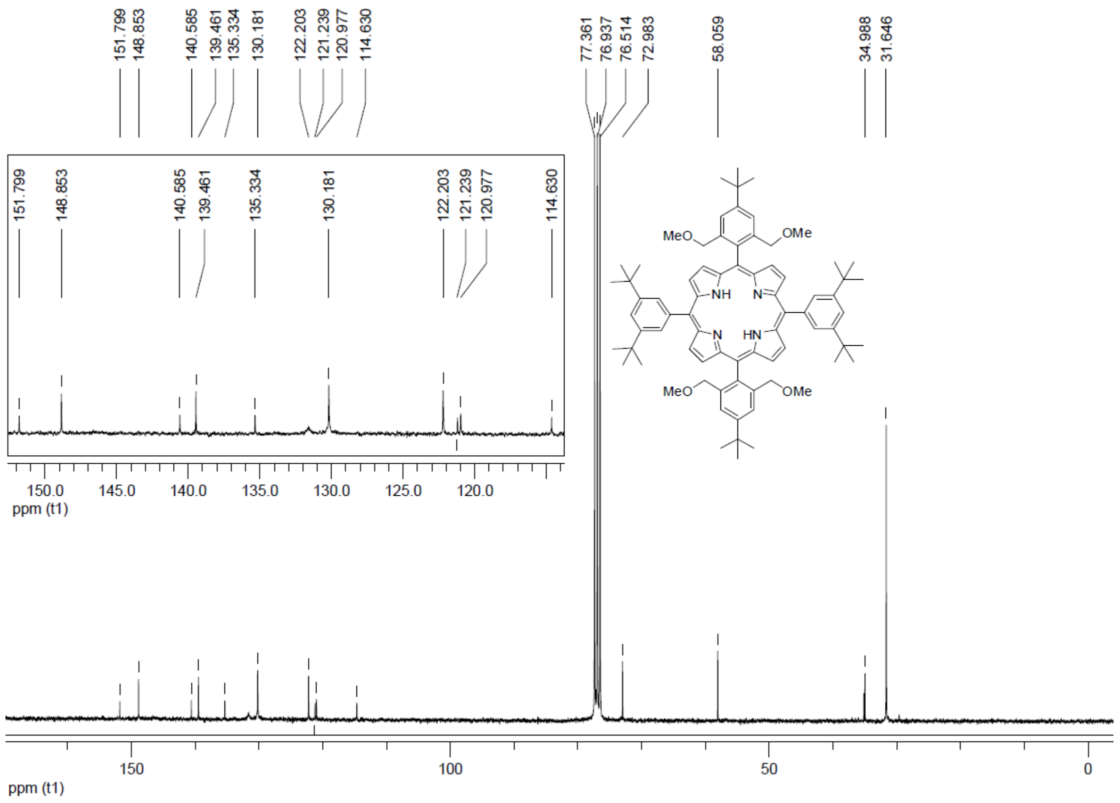

Fig. S28. ^{13}C NMR spectrum of compound **8** (500 MHz, $CDCl_3$, rt).

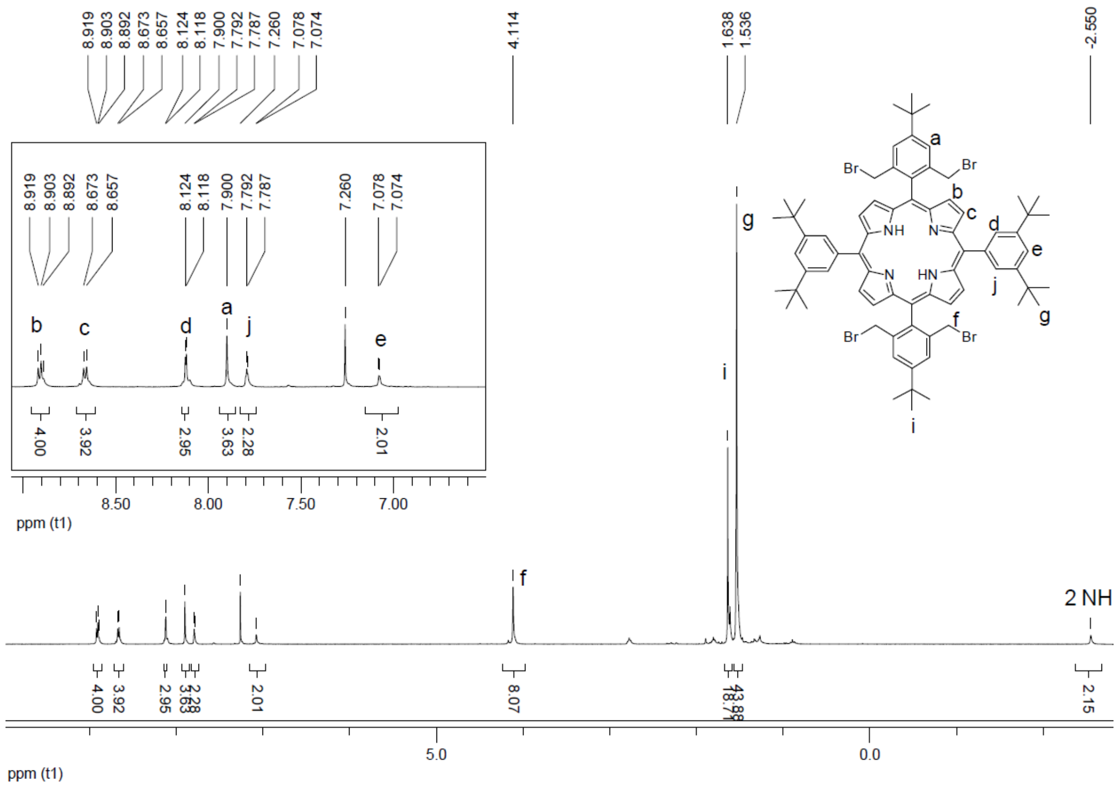

Fig. S29. ^{1}H NMR spectrum of compound **9** (500 MHz, $CDCl_3$, rt).

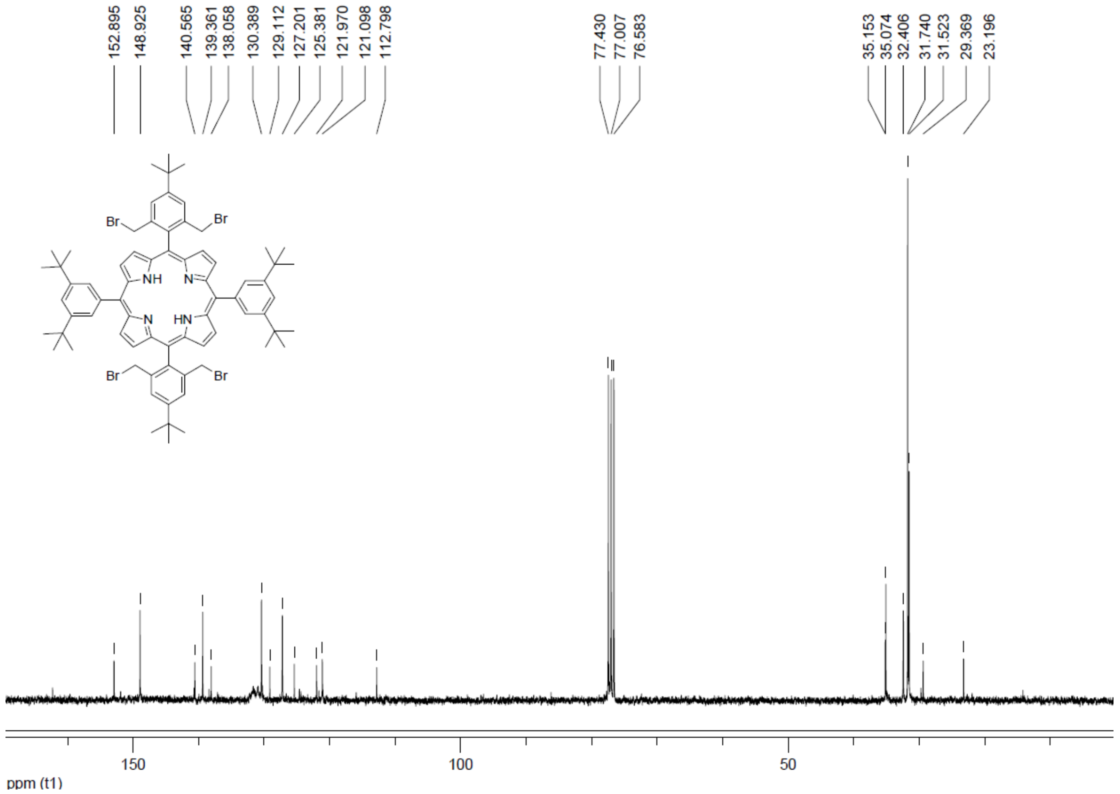

Fig. S30. ^{13}C NMR spectrum of compound **9** (500 MHz, $CDCl_3$, rt).

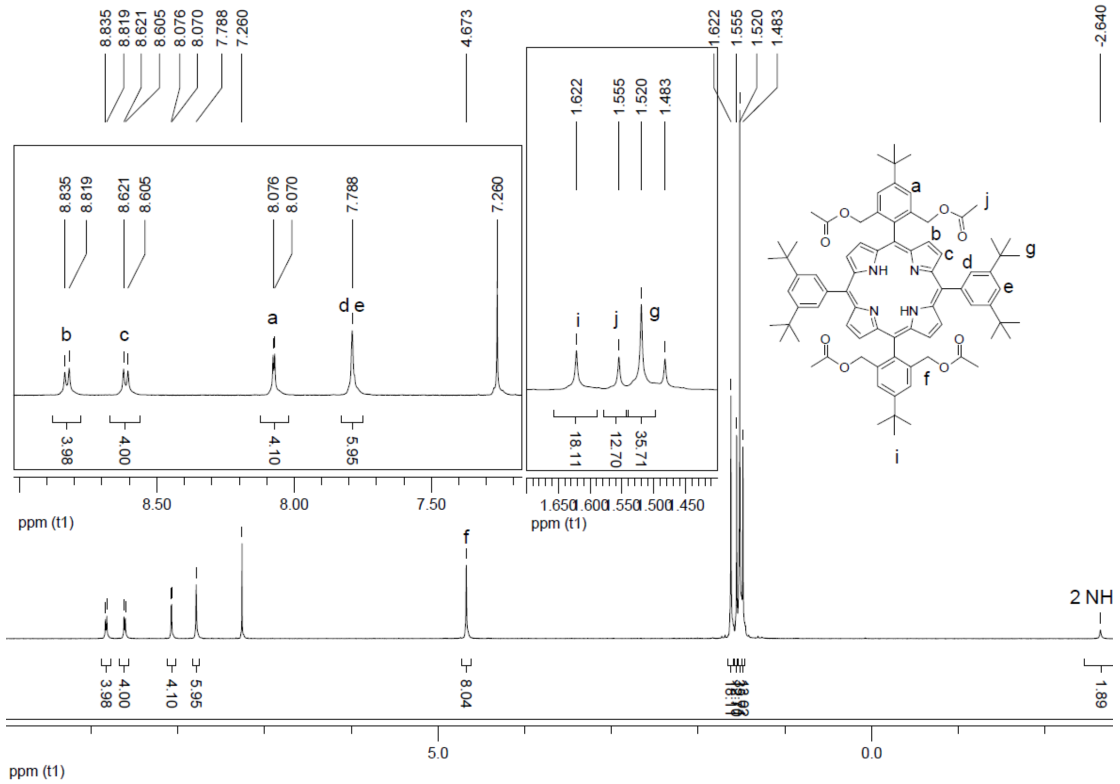

Fig. S31. ^{1}H NMR spectrum of compound **13** (500 MHz, $CDCl_3$, rt).

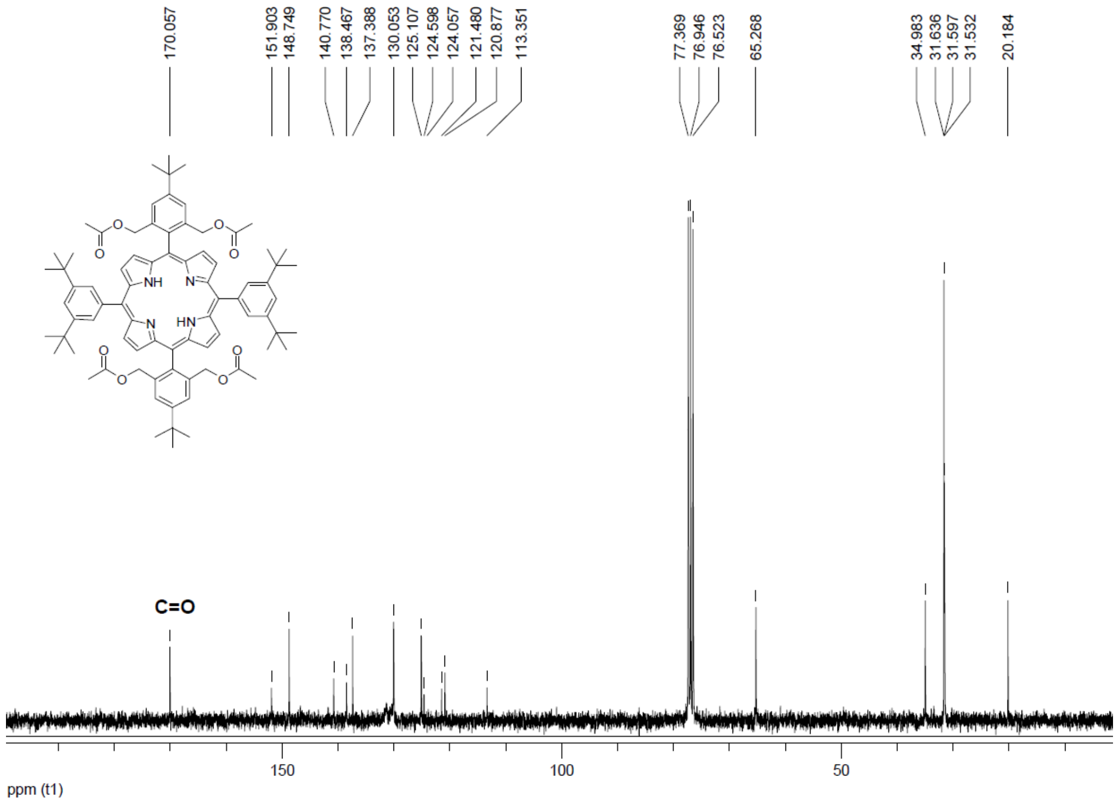

Fig. S32. ^{13}C NMR spectrum of compound **13** (500 MHz, $CDCl_3$, rt).

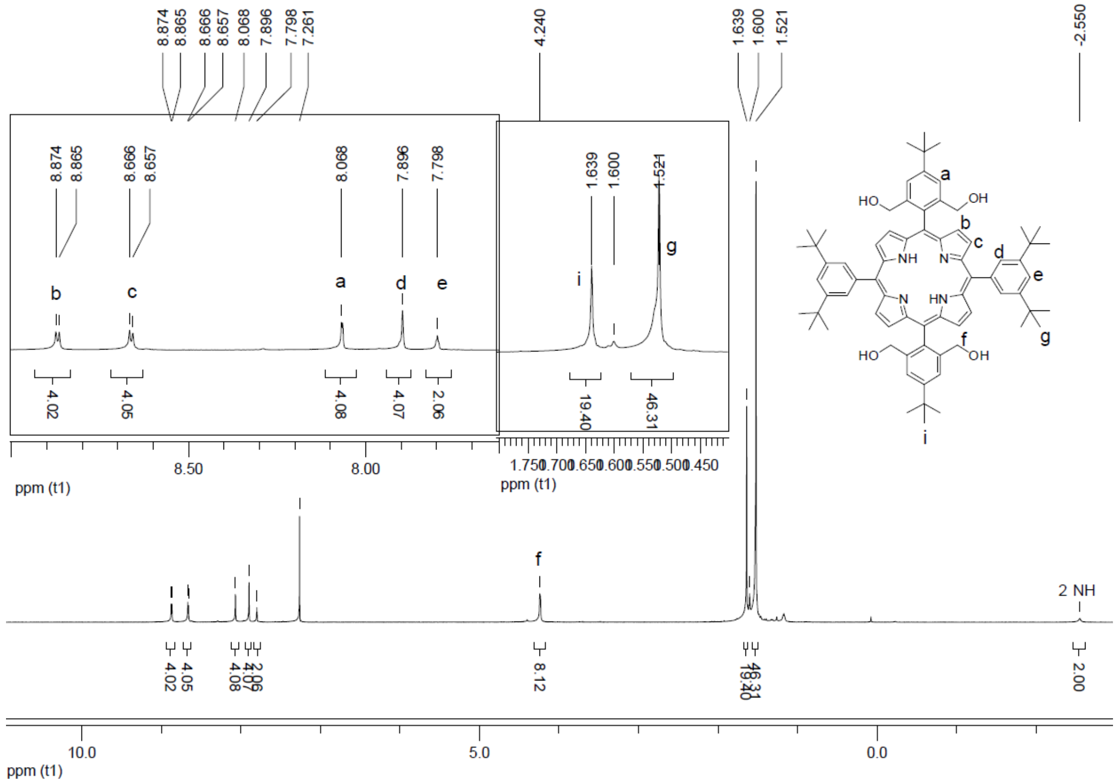

Fig. S33. ^{1}H NMR spectrum of compound **14** (500 MHz, $CDCl_3$, rt).

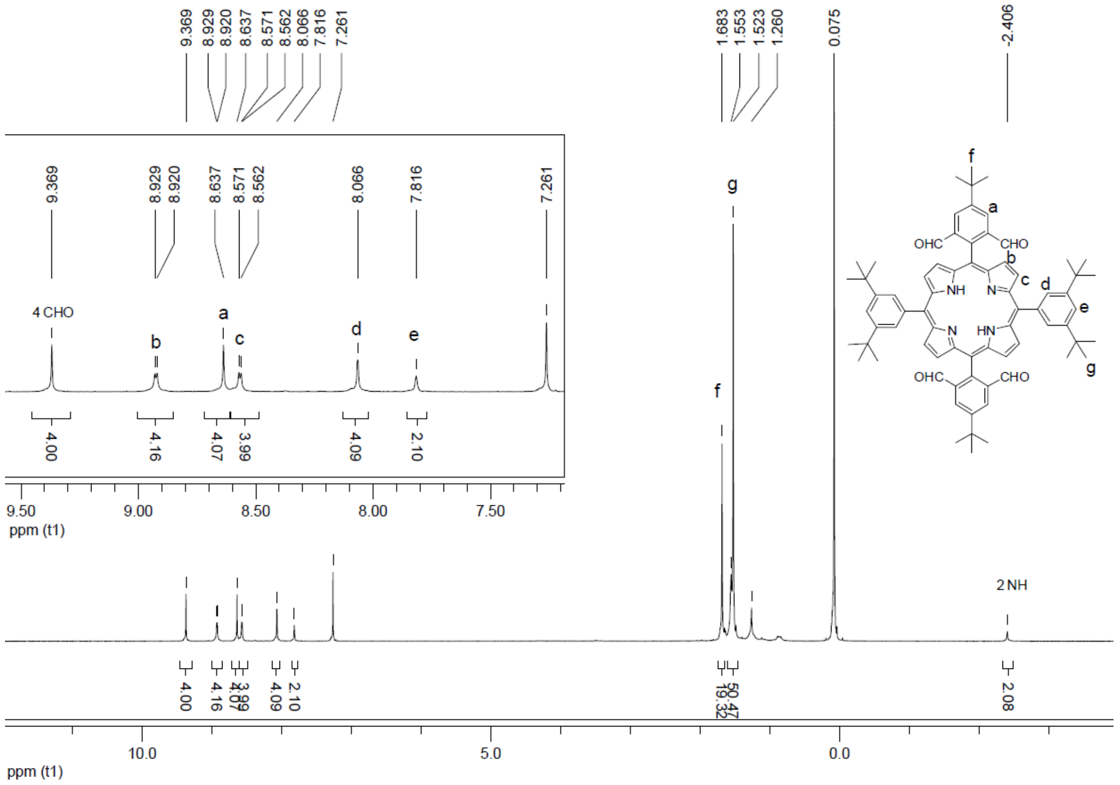

Fig. S34. ^{1}H NMR spectrum of compound **15-H2** (500 MHz, $CDCl_3$, rt).

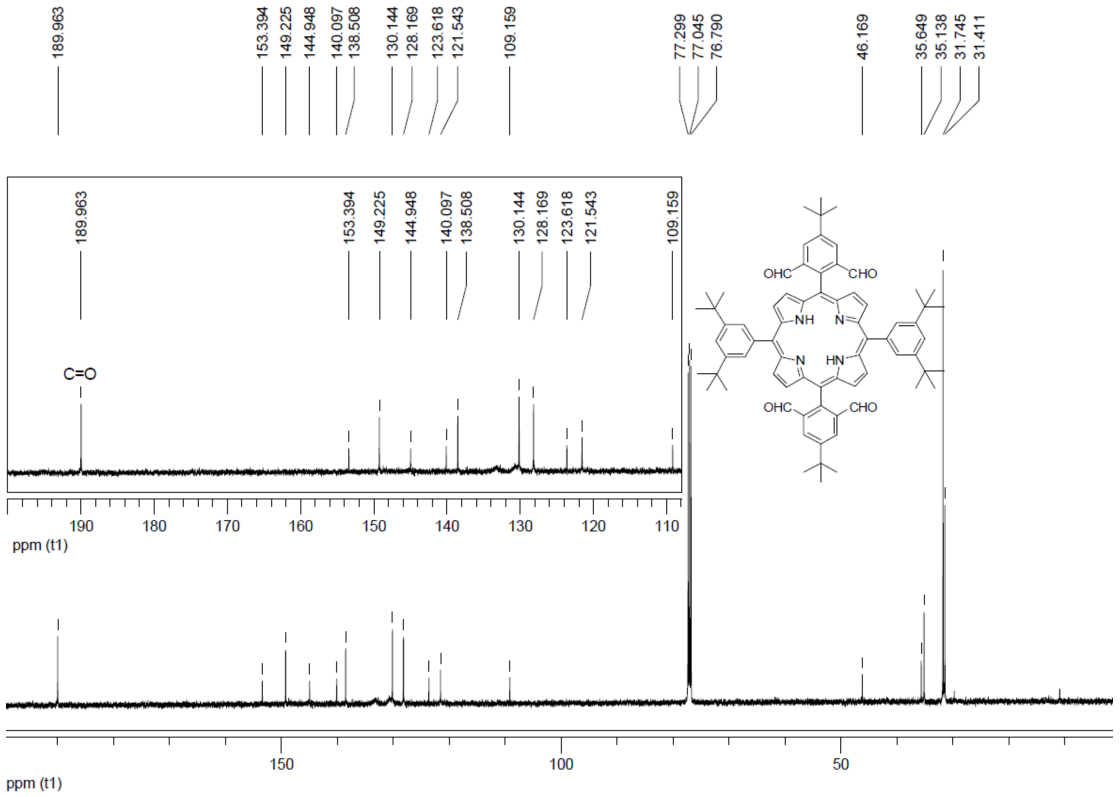

Fig. S35. ^{13}C NMR spectrum of compound **15-H2** (500 MHz, $CDCl_3$, rt).

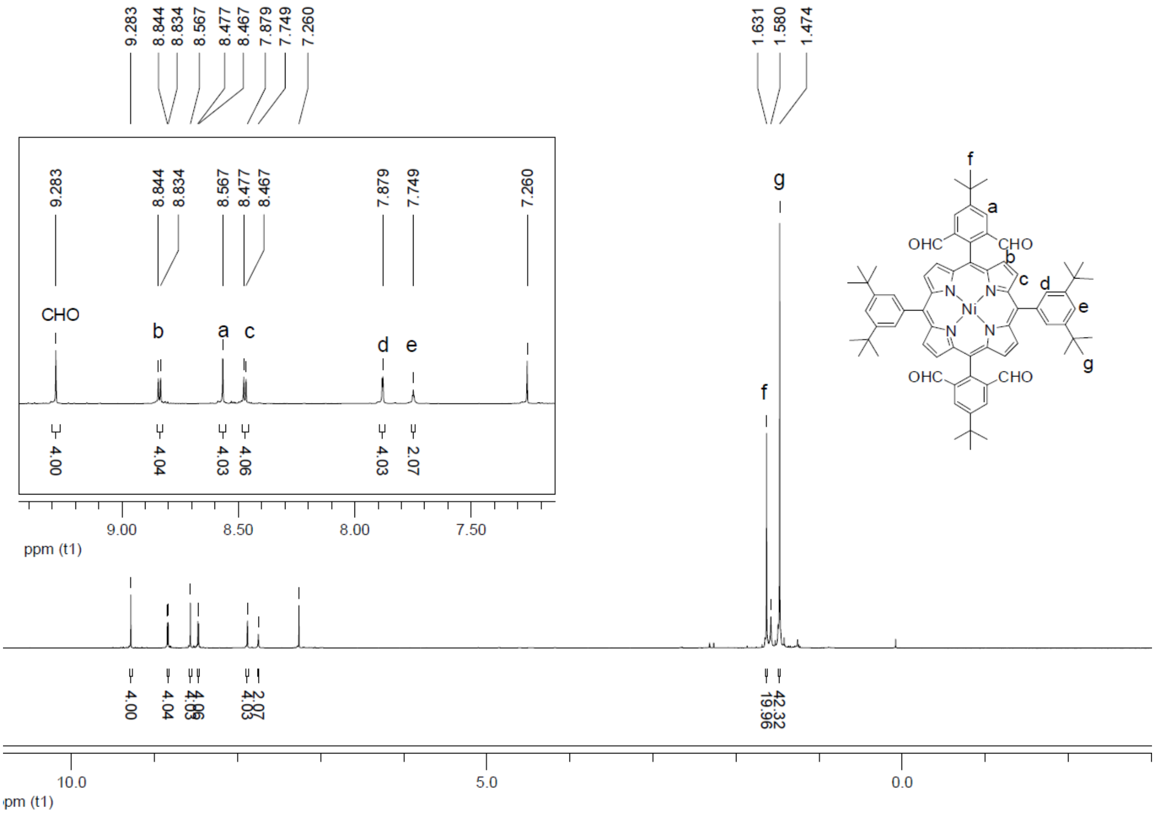

Fig. S36. ^{1}H NMR spectrum of compound **15** (500 MHz, $CDCl_3$, rt)

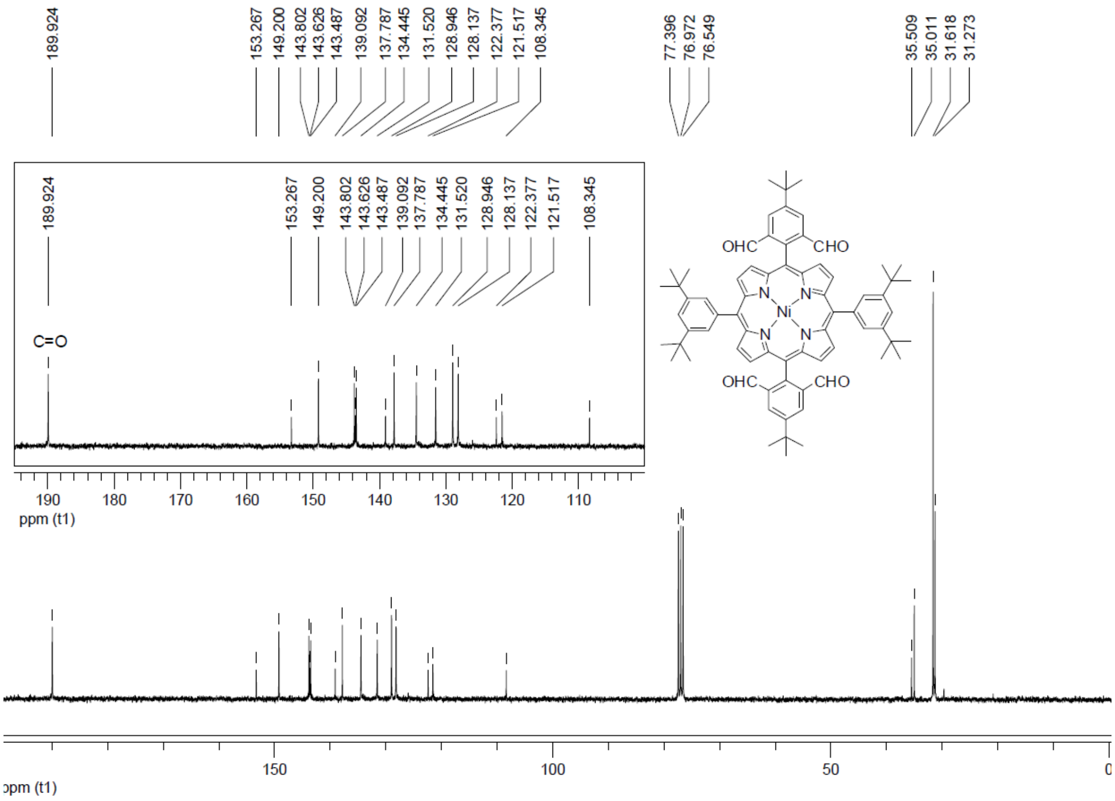

Fig. S37. ^{13}C NMR spectrum of compound **15** (500 MHz, $CDCl_3$, rt).

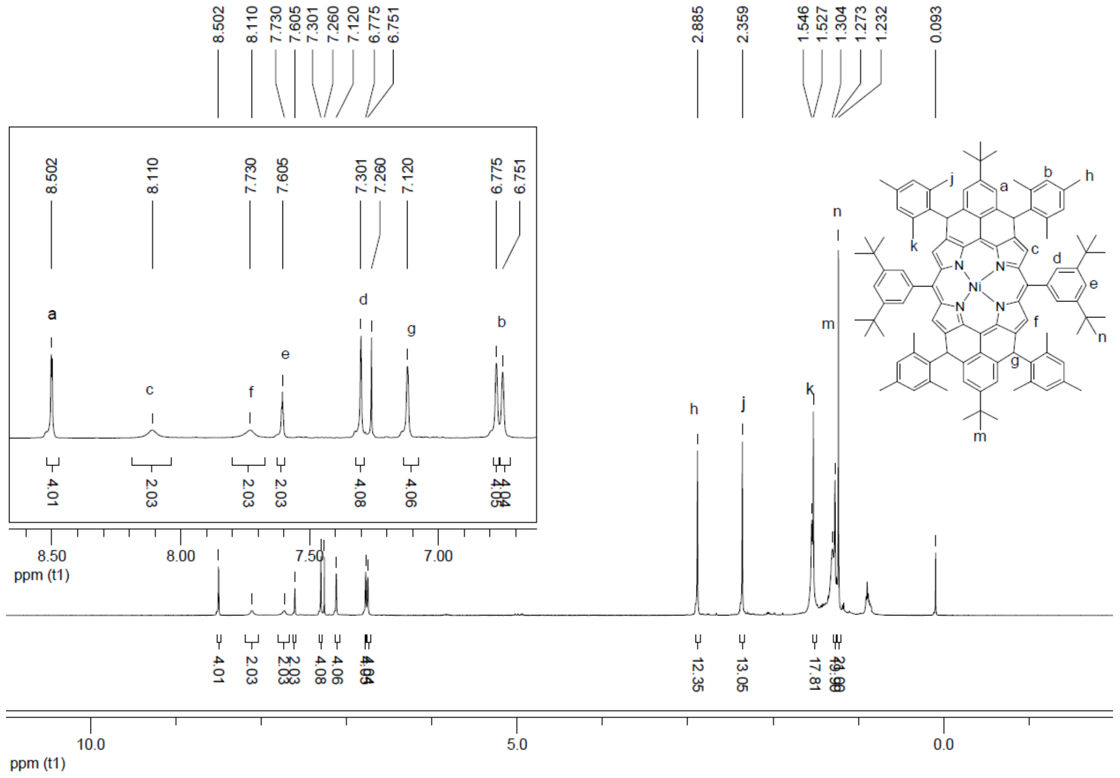

Fig. S38. ^{1}H NMR spectrum of compound **10** (500 MHz, $CDCl_3$, rt)

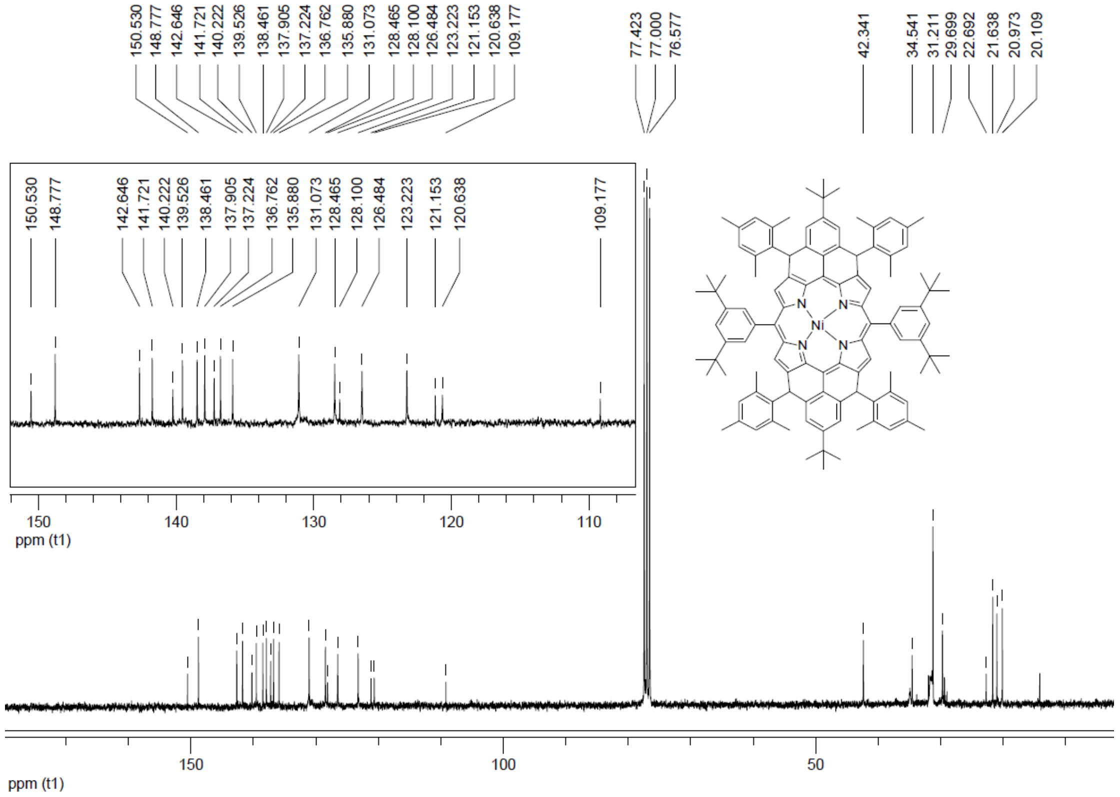

Fig. S39. ^{13}C NMR spectrum of compound **10** (500 MHz, $CDCl_3$, rt).

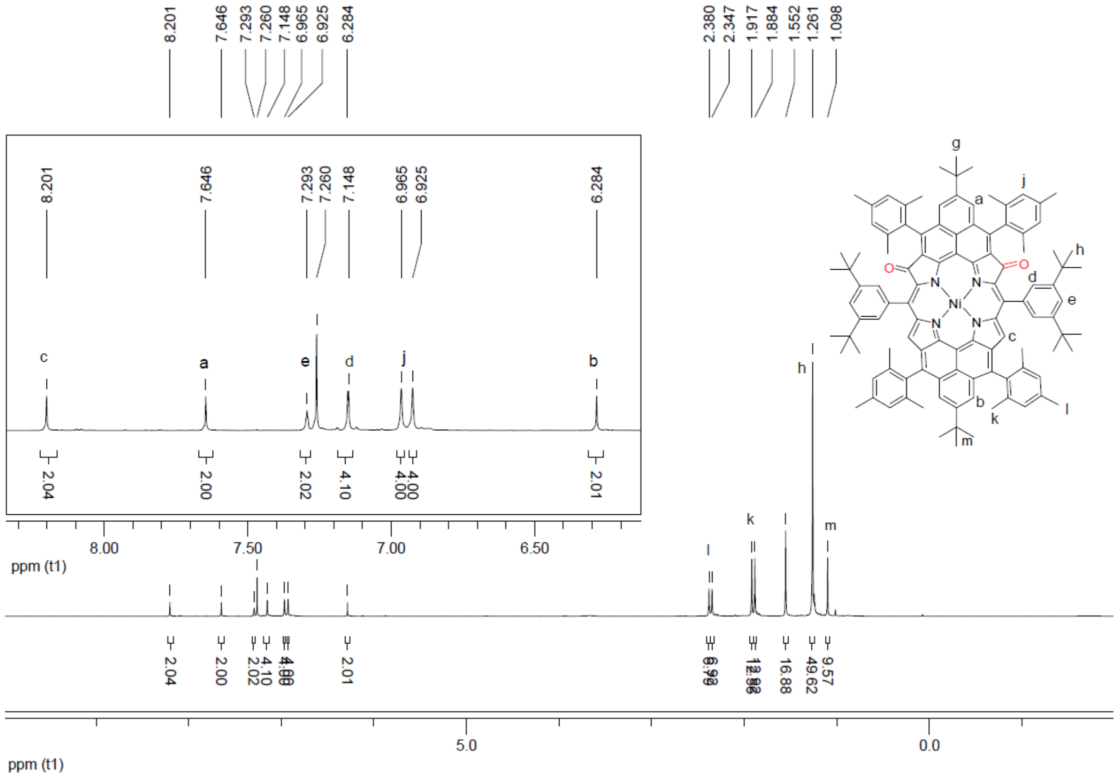

Fig. S40. ^{1}H NMR spectrum of compound **11a** (500 MHz, $CDCl_3$, rt)

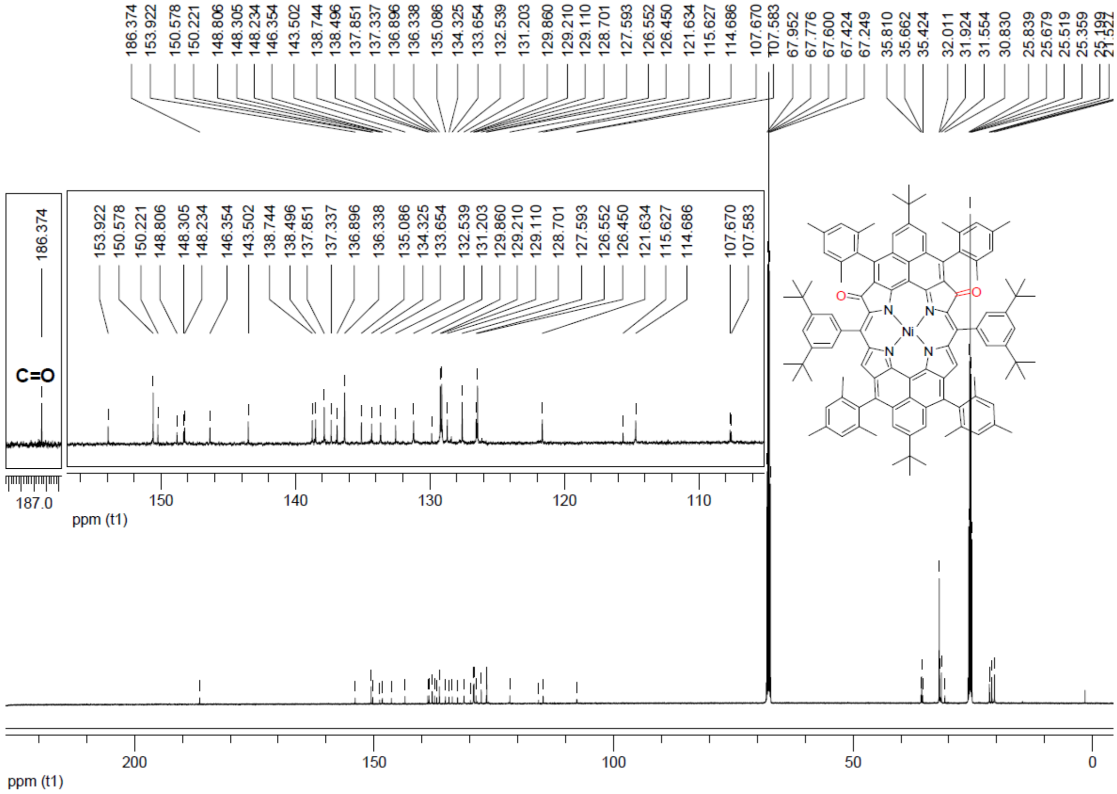

Fig. S41. ^{13}C NMR spectrum of compound **11a** (500 MHz, THF-*d*8, rt).

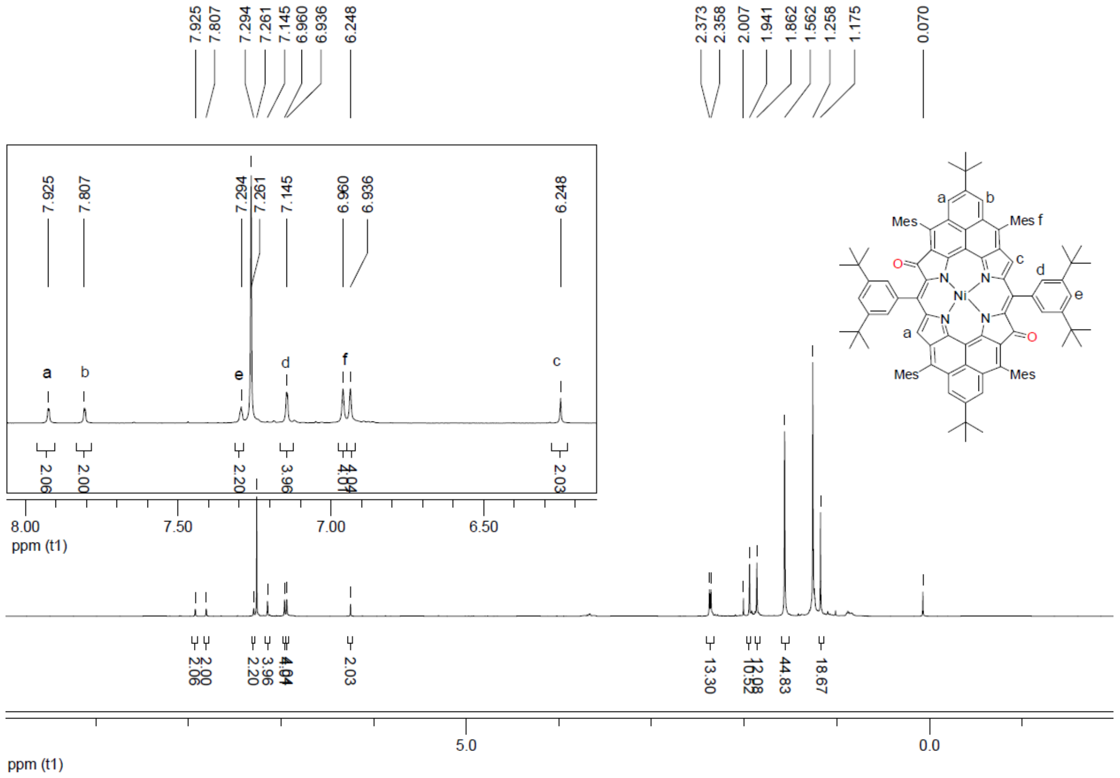

Fig. S42. ^{1}H NMR spectrum of compound **11b** (500 MHz, $CDCl_3$, rt)

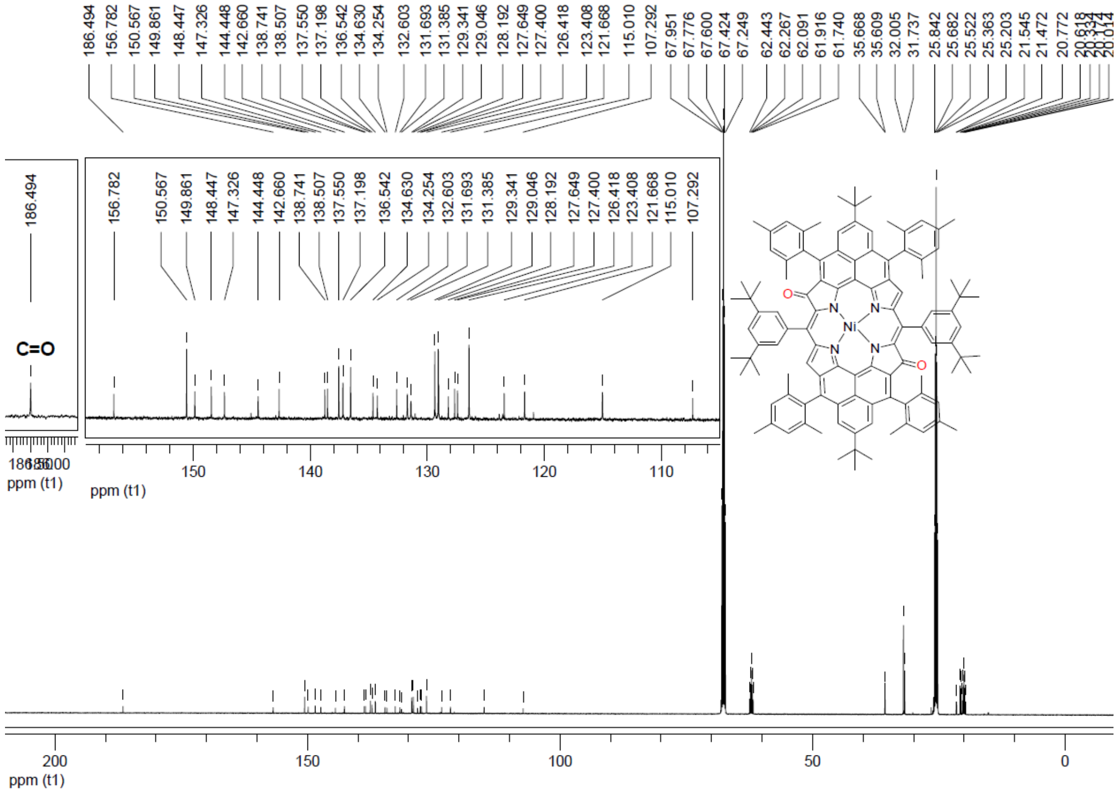

Fig. S43. ^{13}C NMR spectrum of compound **11b** (500 MHz, THF-*d*8, rt).

Mass Spectrum SmartFormula Report

Analysis Info

Analysis Name	D:\Data\Chemistry\2013 Sample\Jan 2013\PoroCOCH3.d	Acquisition Date	1/4/2013 5:32:26 PM
Method	tune_wide_pos_200ul.m	Operator	default user
Sample Name	PoroCOCH3	Instrument / Ser#	micrOTOF-Q II 10269
Comment	A/P WU JISHAN		

Acquisition Parameter

Source Type	APCI	Ion Polarity	Positive	Set Nebulizer	3.0 Bar
Focus	Active	Set Capillary	4000 V	Set Dry Heater	250 °C
Scan Begin	50 m/z	Set End Plate Offset	-500 V	Set Dry Gas	4.0 l/min
Scan End	3000 m/z	Set Collision Cell RF	700.0 Vpp	Set Divert Valve	Waste

Meas. m/z	#	Formula	m/z	err [ppm]	rdb	e⁻ Conf	N-Rule
983.5491	1	C 68 H 73 N O 5	983.5483	-0.8	33.0	odd	ok
	2	C 66 H 71 N 4 O 4	983.5470	-2.2	33.5	even	ok
	3	C 71 H 71 N 2 O 2	983.5510	1.9	37.5	even	ok

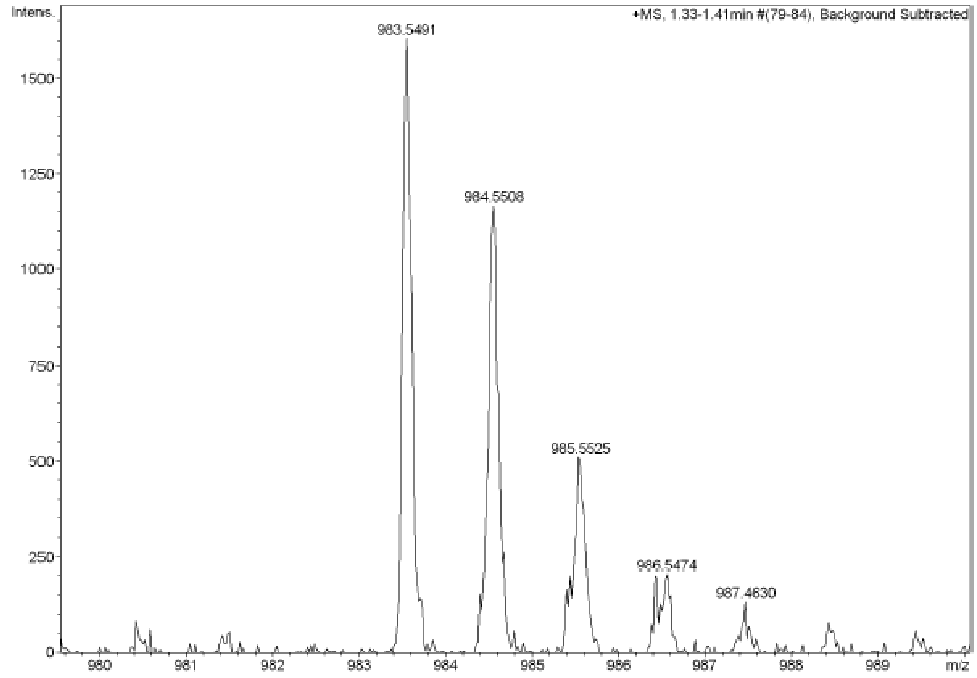

Bruker Compass DataAnalysis 4.0 printed: 1/4/2013 5:39:16 PM Page 1 of 1

Fig. S44. HR mass spectrum (APCI) of the compound **4**.

Mass Spectrum SmartFormula Report

Analysis Info

Analysis Name	D:\Data\Chemistry\2013 Sample\Jan 2013\Por(CH2OH)2.d	Acquisition Date	1/4/2013 5:25:53 PM
Method	tune_wide_pos_200ul.m	Operator	default user
Sample Name	Por(CH2OH)2	Instrument / Ser#	micrOTOF-Q II 10269
Comment	A/P WU JISHAN		

Acquisition Parameter

Source Type	APCI	Ion Polarity	Positive	Set Nebulizer	3.0 Bar
Focus	Active	Set Capillary	4000 V	Set Dry Heater	250 °C
Scan Begin	50 m/z	Set End Plate Offset	-500 V	Set Dry Gas	4.0 l/min
Scan End	3000 m/z	Set Collision Cell RF	700.0 Vpp	Set Divert Valve	Waste

Meas. m/z	#	Formula	m/z	err [ppm]	rdb	e¯ Conf	N-Rule
899.5257	1	C 62 H 67 N 4 O 2	899.5259	0.1	31.5	even	ok
	2	C 64 H 69 N O 3	899.5272	1.6	31.0	odd	ok
	3	C 67 H 67 N 2	899.5299	4.6	35.5	even	ok

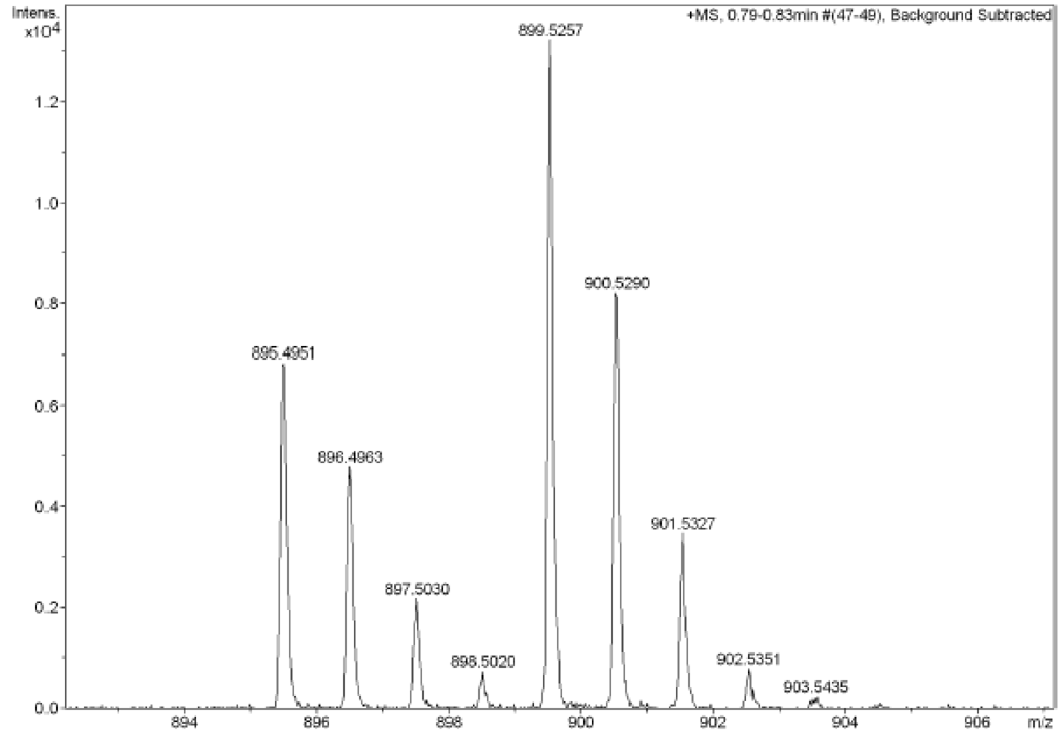

Bruker Compass DataAnalysis 4.0 printed: 1/4/2013 5:29:56 PM Page 1 of 1

Fig. S45. HR mass spectrum (APCI) of the compound **5**.

Mass Spectrum SmartFormula Report

Analysis Info

Analysis Name	D:\Data\Chemistry\2013 Sample\Jan 2013\Por(CHO)2-S.d	Acquisition Date	1/4/2013 5:15:48 PM
Method	tune_wide_pos_200ul.m	Operator	default user
Sample Name	Por(CHO)2-S	Instrument / Ser#	micrOTOF-Q II 10269
Comment	A/P WU JISHAN		

Acquisition Parameter

Source Type	APCI	Ion Polarity	Positive	Set Nebulizer	3.0 Bar
Focus	Active	Set Capillary	4000 V	Set Dry Heater	250 °C
Scan Begin	50 m/z	Set End Plate Offset	-500 V	Set Dry Gas	4.0 l/min
Scan End	3000 m/z	Set Collision Cell RF	700.0 Vpp	Set Divert Valve	Waste

Meas. m/z	#	Formula	m/z	err [ppm]	rdb	e¯ Conf	N-Rule
895.4972	1	C 64 H 65 N O 3	895.4959	-1.5	33.0	odd	ok
	2	C 62 H 63 N 4 O 2	895.4946	-3.0	33.5	even	ok
	3	C 67 H 63 N 2	895.4986	1.5	37.5	even	ok

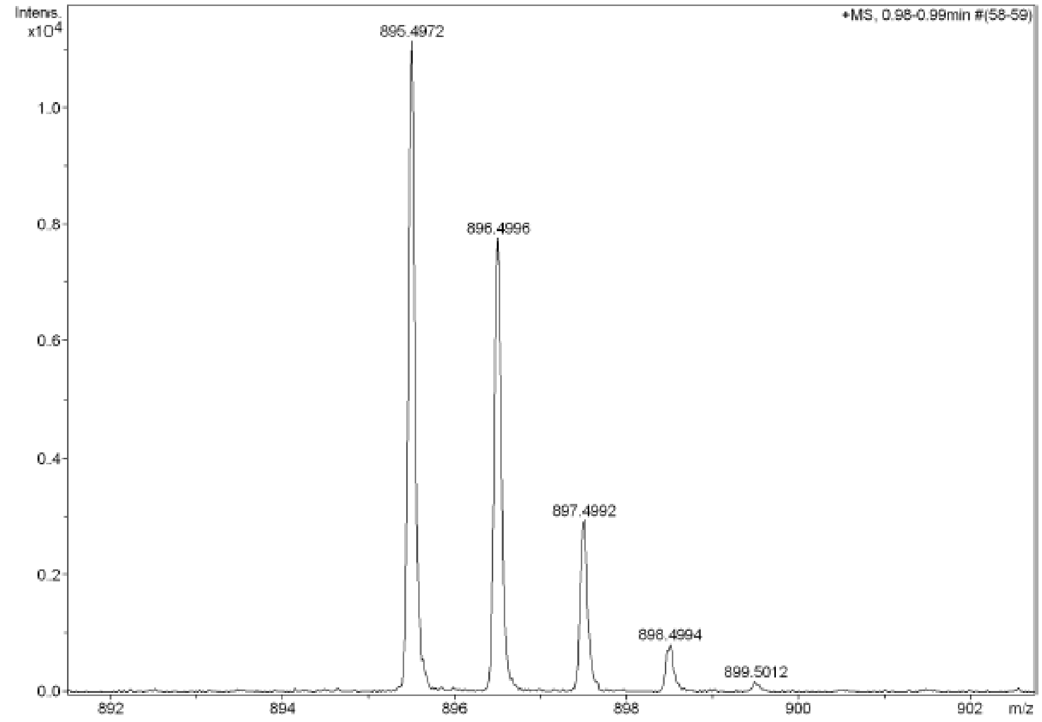

Bruker Compass DataAnalysis 4.0 printed: 1/4/2013 5:21:56 PM Page 1 of 1

Fig. S46. HR mass spectrum (APCI) of the compound **6-H2**.

Mass Spectrum SmartFormula Report

Analysis Info

Analysis Name	D:\Data\Chemistry\2014 Sample\Jul 2014\POR-Ni-2CHO.d	Acquisition Date	7/3/2014 5:28:24 PM
Method	APCI tune_pos-6Dec12.m	Operator	default user
Sample Name	POR-Ni-2CHO	Instrument / Ser#	micrOTOF-Q II 10269
Comment	A/P WU JISHAN		

Acquisition Parameter

Source Type	APCI	Ion Polarity	Positive	Set Nebulizer	3.0 Bar
Focus	Not active	Set Capillary	4500 V	Set Dry Heater	200 °C
Scan Begin	50 m/z	Set End Plate Offset	-500 V	Set Dry Gas	4.0 l/min
Scan End	3000 m/z	Set Collision Cell RF	700.0 Vpp	Set Divert Valve	Waste

Meas. m/z	#	Formula	m/z	err [ppm]	rdb	e¯ Conf	N-Rule
951.4176	1	C 62 H 61 N 4 Ni O 2	951.4143	-3.5	34.5	even	ok
	2	C 55 H 65 N 4 Ni O 7	951.4201	2.6	25.5	even	ok

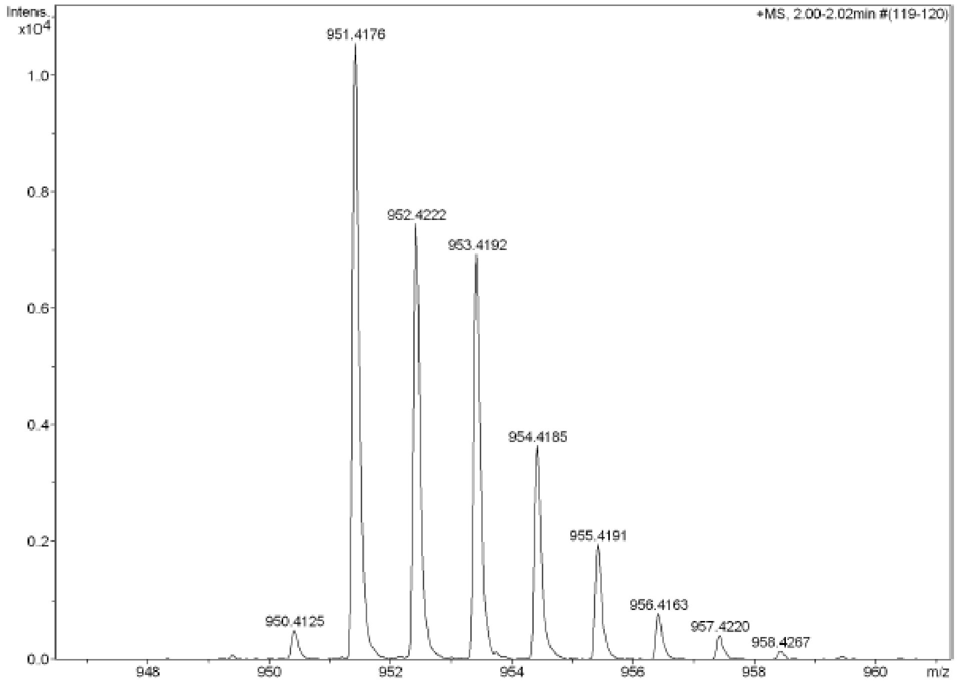

Bruker Compass DataAnalysis 4.0 printed: 7/3/2014 5:33:18 PM Page 1 of 1

Fig. S47. HR mass spectrum (APCI) of the compound **6**.

Mass Spectrum SmartFormula Report

Analysis Info

Analysis Name	D:\Data\Chemistry\2014 Sample\May 2014\Ni-3-1.d	Acquisition Date	5/22/2014 10:14:48 AM
Method	APCI tune_pos-6Dec12.m	Operator	default user
Sample Name	Ni-3	Instrument / Ser#	micrOTOF-Q II 10269
Comment	A/P WU JISHAN		

Acquisition Parameter

Source Type	APCI	Ion Polarity	Positive	Set Nebulizer	3.0 Bar
Focus	Not active	Set Capillary	4500 V	Set Dry Heater	200 °C
Scan Begin	50 m/z	Set End Plate Offset	-500 V	Set Dry Gas	4.0 l/min
Scan End	3000 m/z	Set Collision Cell RF	700.0 Vpp	Set Divert Valve	Waste

Meas. m/z	#	Formula	m/z	err [ppm]	rdb	e⁻ Conf	N-Rule
1155.5776	1	C 80 H 81 N 4 Ni	1155.5809	2.9	42.5	even	ok

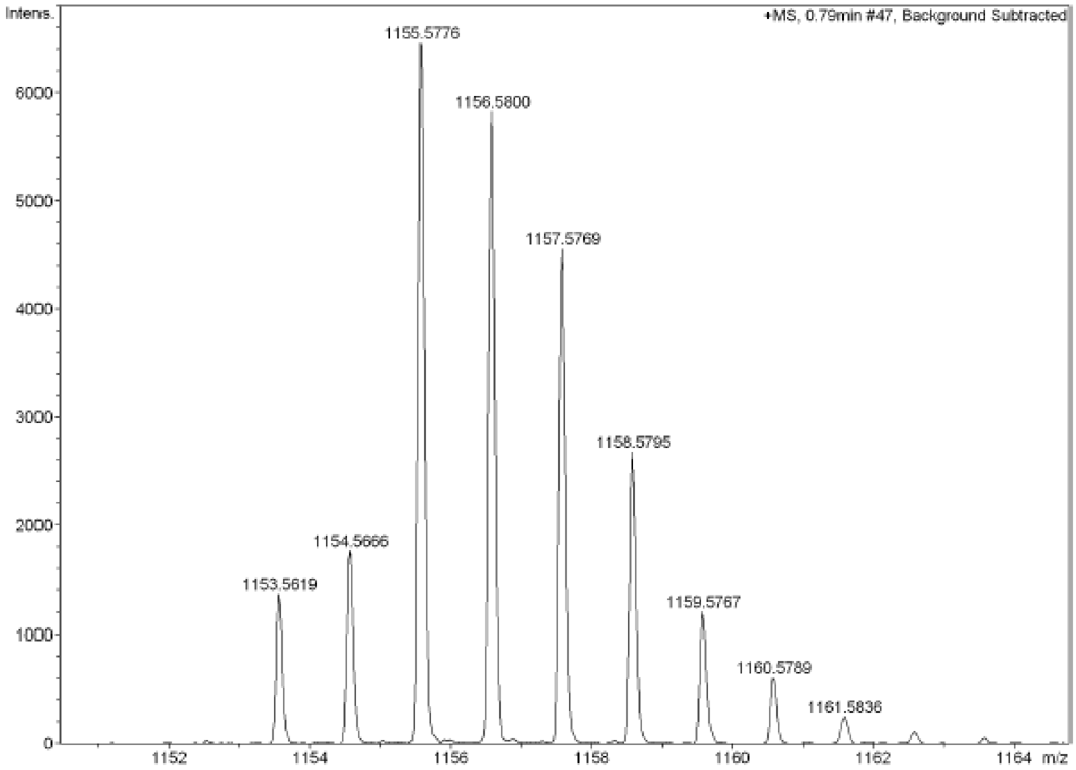

Bruker Compass DataAnalysis 4.0 printed: 5/22/2014 10:19:52 AM Page 1 of 1

Fig. S48. HR mass spectrum (APCI) of the compound **7**.

Mass Spectrum SmartFormula Report

Analysis Info

Analysis Name	D:\Data\Chemistry\2014 Sample\Jun 2014\Ni-3.d	Acquisition Date	6/25/2014 5:03:19 PM
Method	APCI tune_pos-6Dec12.m	Operator	default user
Sample Name	Ni-3	Instrument / Ser#	micrOTOF-Q II 10269
Comment	A/P WU J S		

Acquisition Parameter

Source Type	APCI	Ion Polarity	Positive	Set Nebulizer	3.0 Bar
Focus	Not active	Set Capillary	4500 V	Set Dry Heater	200 °C
Scan Begin	50 m/z	Set End Plate Offset	-500 V	Set Dry Gas	4.0 l/min
Scan End	3000 m/z	Set Collision Cell RF	500.0 Vpp	Set Divert Valve	Waste

Meas. m/z	#	Formula	m/z	err [ppm]	rdb	e¯ Conf	N-Rule
1153.5662	1	C 80 H 79 N 4 Ni	1153.5653	-0.8	43.5	even	ok

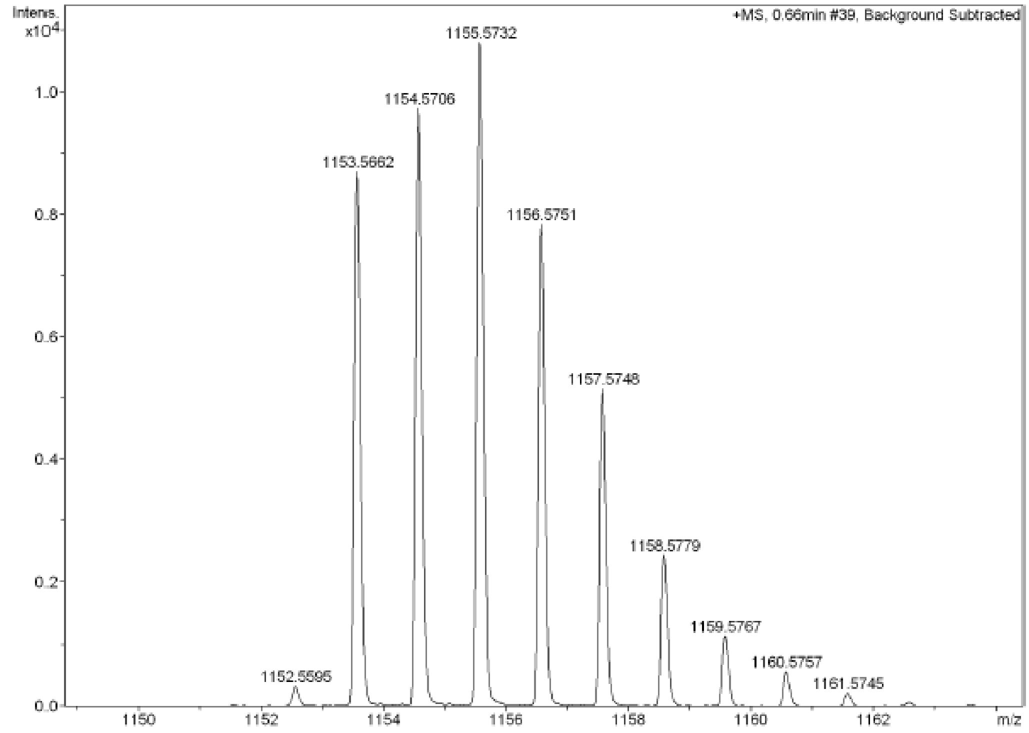

Bruker Compass DataAnalysis 4.0 printed: 6/25/2014 5:08:34 PM Page 1 of 1

Fig. S49. HR mass spectrum (APCI) of the compound **1**.

Mass Spectrum SmartFormula Report

Analysis Info

Analysis Name	D:\Data\Chemistry\2014 Sample\May 2014\Ni-4.d	Acquisition Date	5/22/2014 9:52:07 AM
Method	APCI tune_pos-6Dec12.m	Operator	default user
Sample Name	Ni-4	Instrument / Ser#	micrOTOF-Q II 10269
Comment	A/P WU JISHAN		

Acquisition Parameter

Source Type	APCI	Ion Polarity	Positive	Set Nebulizer	3.0 Bar
Focus	Not active	Set Capillary	4500 V	Set Dry Heater	200 °C
Scan Begin	50 m/z	Set End Plate Offset	-500 V	Set Dry Gas	4.0 l/min
Scan End	3000 m/z	Set Collision Cell RF	700.0 Vpp	Set Divert Valve	Waste

Meas. m/z	#	Formula	m/z	err [ppm]	rdb	e¯ Conf	N-Rule
1155.5762	1	C 80 H 81 N 4 Ni	1155.5809	4.1	42.5	even	ok

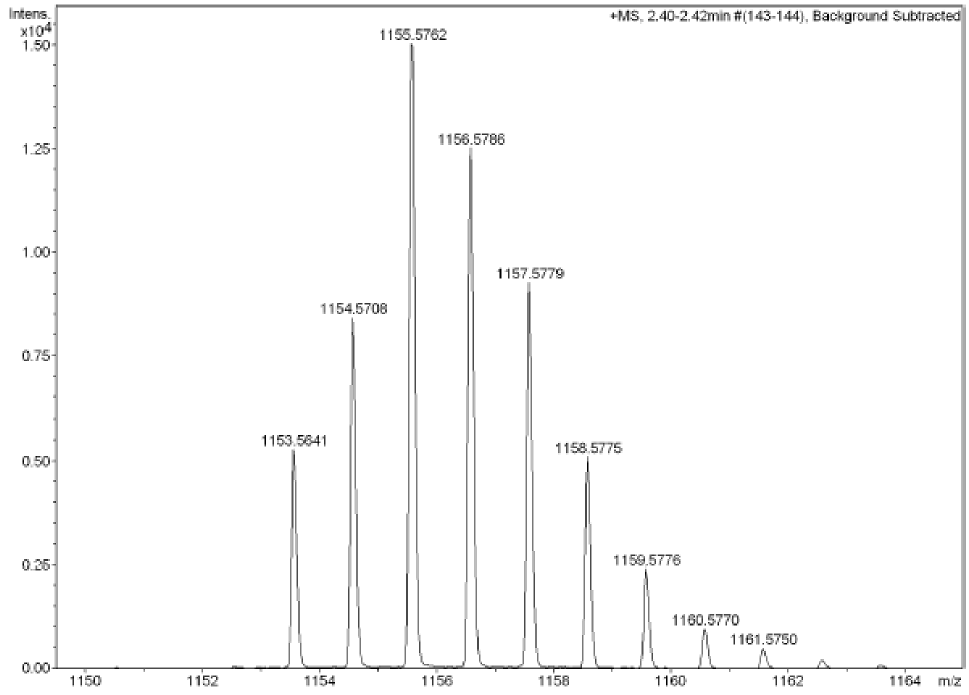

Bruker Compass DataAnalysis 4.0 printed: 5/22/2014 9:59:57 AM Page 1 of 1

Fig. S50. HR mass spectrum (APCI) of the compound **1-H2** (after crystal growing).

Mass Spectrum SmartFormula Report

Analysis Info

Analysis Name	D:\Data\Chemistry\2014 Sample\Jun 2014\Por-4OMe.d	Acquisition Date	6/25/2014 5:39:32 PM
Method	APCI tune_pos-6Dec12.m	Operator	default user
Sample Name	Por-4OMe	Instrument / Ser#	micrOTOF-Q II 10269
Comment	A/P WU J S		

Acquisition Parameter

Source Type	APCI	Ion Polarity	Positive	Set Nebulizer	3.0 Bar		
Focus	Not active	Set Capillary	4500 V	Set Dry Heater	200 °C		
Scan Begin	50 m/z	Set End Plate Offset	-500 V	Set Dry Gas	4.0 l/min		
Scan End	3000 m/z	Set Collision Cell RF	500.0 Vpp	Set Divert Valve	Waste		

Meas. m/z	#	Formula	m/z	err [ppm]	rdb	e⁻ Conf	N-Rule
1127.7368	1	C 76 H 95 N 4 O 4	1127.7348	-1.8	31.5	even	ok

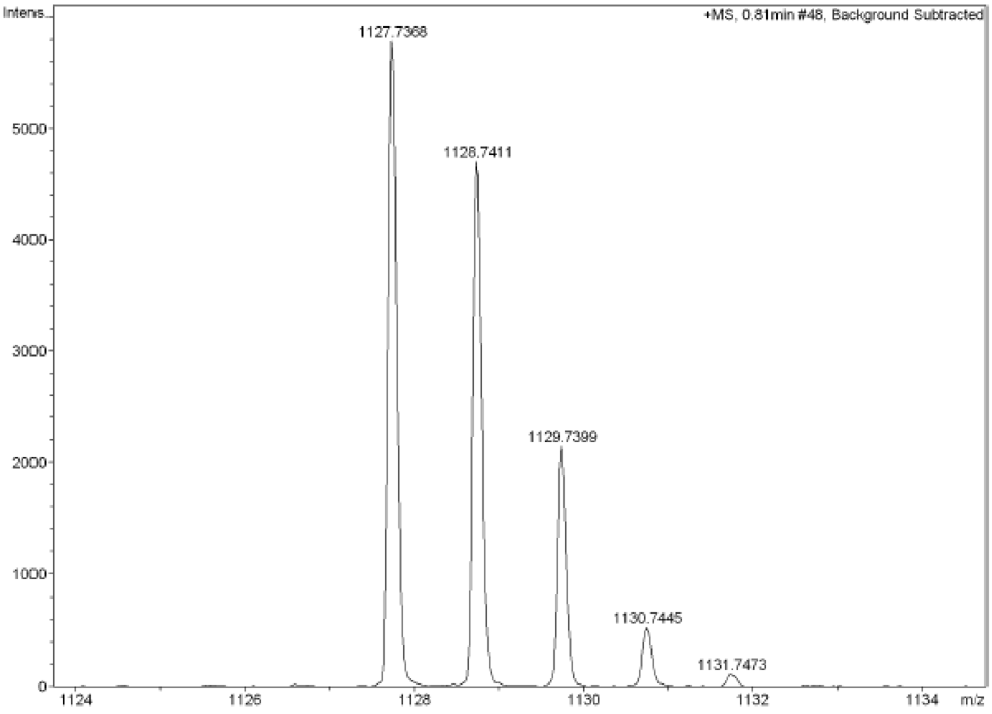

Bruker Compass DataAnalysis 4.0 printed: 6/25/2014 5:42:57 PM Page 1 of 1

Fig. S51. HR mass spectrum (APCI) of the compound **8**.

Mass Spectrum SmartFormula Report

Analysis Info

Analysis Name	D:\Data\Chemistry\2014 Sample\Jun 2014\Por-4Br.d	Acquisition Date	6/25/2014 6:39:46 PM
Method	APCI tune_pos-6Dec12.m	Operator	default user
Sample Name	Por-4Br	Instrument / Ser#	micrOTOF-Q II 10269
Comment	A/P WU J S		

Acquisition Parameter

Source Type	APCI	Ion Polarity	Positive	Set Nebulizer	3.0 Bar
Focus	Not active	Set Capillary	4500 V	Set Dry Heater	200 °C
Scan Begin	50 m/z	Set End Plate Offset	-500 V	Set Dry Gas	4.0 l/min
Scan End	3000 m/z	Set Collision Cell RF	500.0 Vpp	Set Divert Valve	Waste

Meas. m/z	#	Formula	m/z	err [ppm]	rdb	e⁻ Conf	N-Rule
1319.3364	1	C 72 H 83 Br 4 N 4	1319.3346	-1.4	31.5	even	ok
	2	C 64 H 94 Br 2 N 4 ^81Br 3	1319.3328	-2.7	17.5	even	ok
1321.3379	1	C 87 H 63 Br 2 N 4	1321.3414	2.6	57.5	even	ok
	2	C 72 H 83 Br 3 N 4 ^81Br	1321.3325	-4.1	31.5	even	ok
1323.3353	1	C 87 H 63 Br N 4 ^81Br	1323.3394	3.1	57.5	even	ok
	2	C 72 H 83 Br 2 N 4 ^81Br 2	1323.3305	-3.6	31.5	even	ok

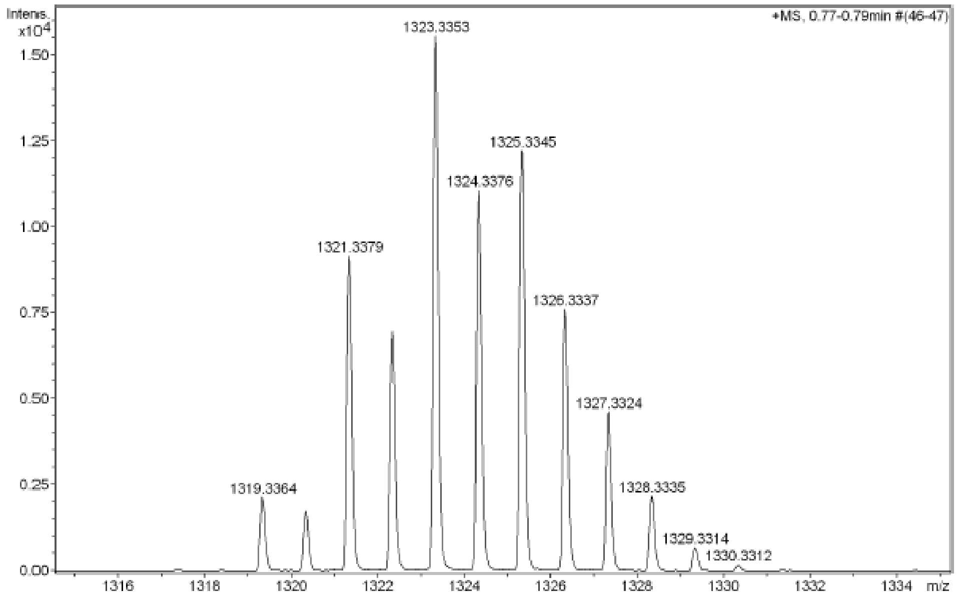

Bruker Compass DataAnalysis 4.0	printed:	6/25/2014 6:46:12 PM	Page 1 of 1

Fig. S52. HR mass spectrum (APCI) of the compound **9**.

Mass Spectrum SmartFormula Report

Analysis Info

		Acquisition Date	7/8/2014 10:52:56 AM
Analysis Name	D:\Data\Chemistry\2014 Sample\Jul 2014\POR(CH2OCOCH3)4-0708-1.d		
Method	APCI tune_pos-6Dec12.m	Operator	default user
Sample Name	POR(CH2OCOCH3)4	Instrument / Ser#	micrOTOF-Q II 10269
Comment	A/P Wu JiShan		

Acquisition Parameter

Source Type	APCI	Ion Polarity	Positive	Set Nebulizer	4.0 Bar
Focus	Not active	Set Capillary	4500 V	Set Dry Heater	200 °C
Scan Begin	50 m/z	Set End Plate Offset	-500 V	Set Dry Gas	4.0 l/min
Scan End	3000 m/z	Set Collision Cell RF	100.0 Vpp	Set Divert Valve	Waste

Meas. m/z	#	Formula	m/z	err [ppm]	rdb	e¯ Conf	N-Rule
1239.7142	1	C 80 H 95 N 4 O 8	1239.7144	0.2	35.5	even	ok

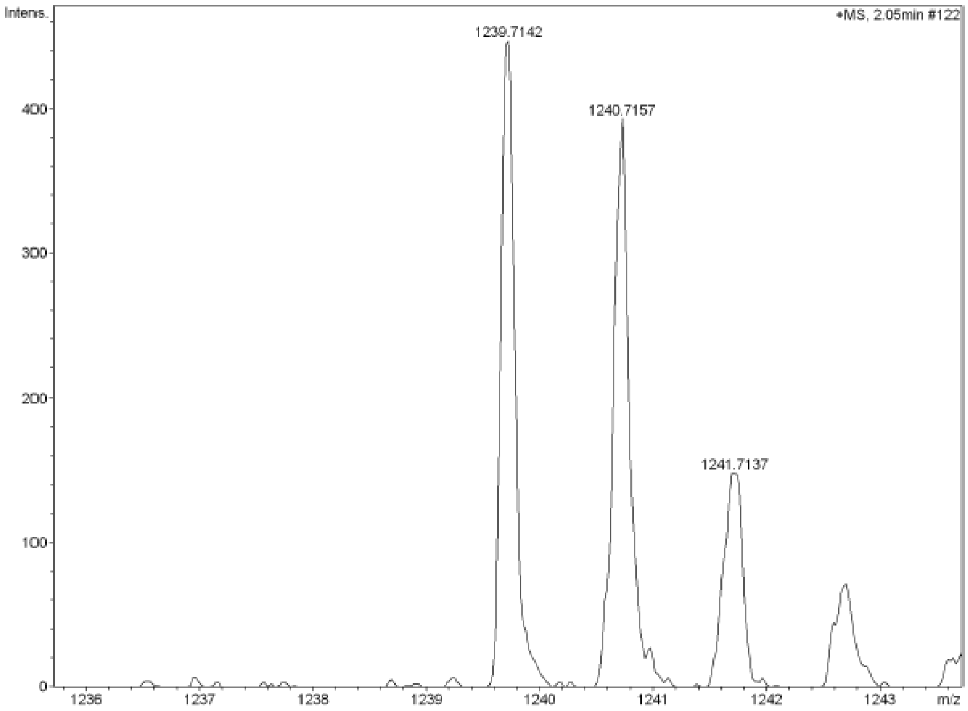

Bruker Compass DataAnalysis 4.0 printed: 7/8/2014 10:58:26 AM Page 1 of 1

Fig. S53. HR mass spectrum (APCI) of the compound **13**.

Mass Spectrum SmartFormula Report

Analysis Info

Analysis Name	D:\Data\Chemistry\2014 Sample\Jun 2014\Por-4OH.d	Acquisition Date	6/25/2014 5:19:13 PM
Method	APCI tune_pos-6Dec12.m	Operator	default user
Sample Name	Por-4OH	Instrument / Ser#	micrOTOF-Q II 10269
Comment	A/P WU J S		

Acquisition Parameter

Source Type	APCI	Ion Polarity	Positive	Set Nebulizer	3.0 Bar
Focus	Not active	Set Capillary	4500 V	Set Dry Heater	200 °C
Scan Begin	50 m/z	Set End Plate Offset	-500 V	Set Dry Gas	4.0 l/min
Scan End	3000 m/z	Set Collision Cell RF	500.0 Vpp	Set Divert Valve	Waste

Meas. m/z	#	Formula	m/z	err [ppm]	rdb	e¯ Conf	N-Rule
1071.6722	1	C 72 H 87 N 4 O 4	1071.6722	0.0	31.5	even	ok
	2	C 72 H 93 N 4 Ni	1071.6748	2.5	28.5	even	ok

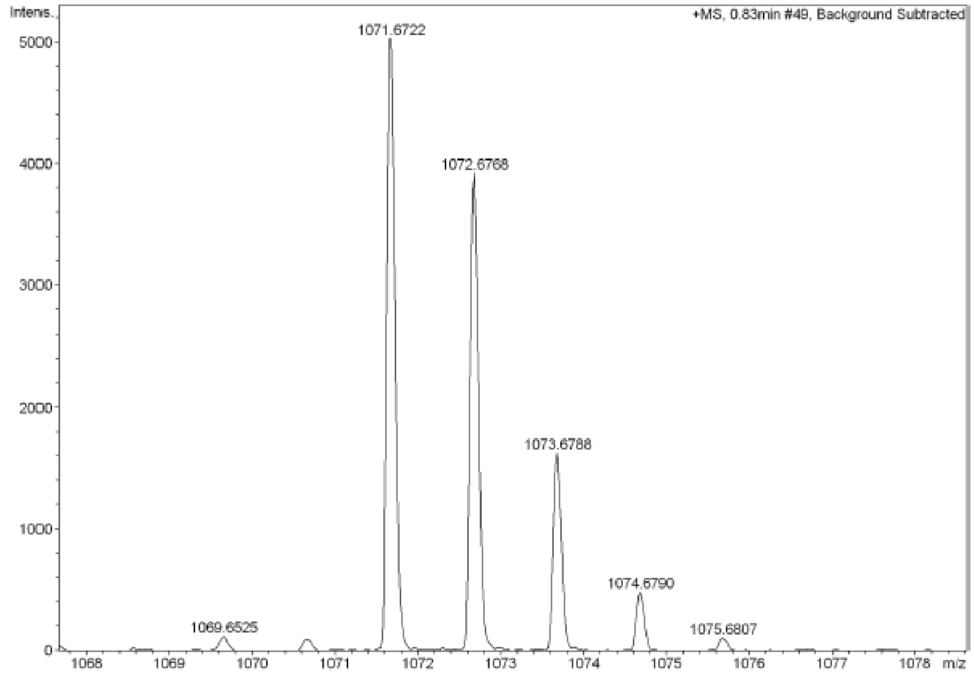

Bruker Compass DataAnalysis 4.0 printed: 6/25/2014 5:22:53 PM Page 1 of 1

Fig. S54. HR mass spectrum (APCI) of the compound **14**.

Mass Spectrum SmartFormula Report

Analysis Info

Analysis Name	D:\Data\Chemistry\2014 Sample\Jun 2014\Por-4CHO.d	Acquisition Date	6/25/2014 6:08:16 PM
Method	APCI tune_pos-6Dec12.m	Operator	default user
Sample Name	Por-4CHO	Instrument / Ser#	micrOTOF-Q II 10269
Comment	A/P WU J S		

Acquisition Parameter

Source Type	APCI	Ion Polarity	Positive	Set Nebulizer	3.0 Bar
Focus	Not active	Set Capillary	4500 V	Set Dry Heater	200 °C
Scan Begin	50 m/z	Set End Plate Offset	-500 V	Set Dry Gas	4.0 l/min
Scan End	3000 m/z	Set Collision Cell RF	500.0 Vpp	Set Divert Valve	Waste

Meas. m/z	#	Formula	m/z	err [ppm]	rdb	e¯ Conf	N-Rule
1063.6117	1	C 72 H 79 N 4 O 4	1063.6096	-2.0	35.5	even	ok

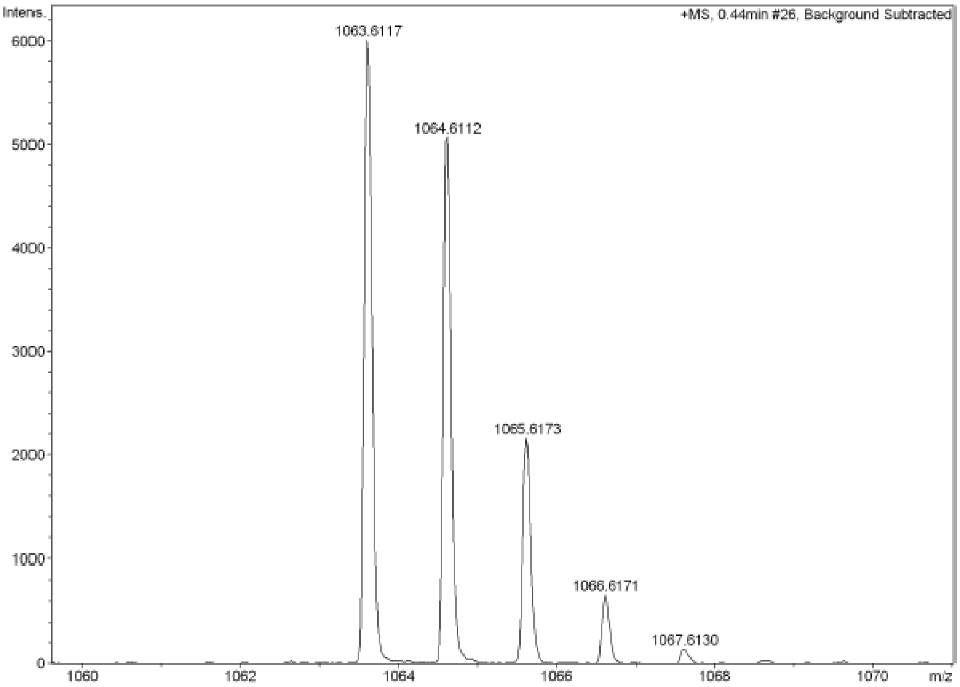

Bruker Compass DataAnalysis 4.0 printed: 6/25/2014 6:11:39 PM Page 1 of 1

Fig. S55. HR mass spectrum (HPCI-HR) of the compound **15-H2**.

Mass Spectrum SmartFormula Report

Analysis Info

Analysis Name	D:\Data\Chemistry\2014 Sample\Jun 2014\Por-4CHONi.d	Acquisition Date	6/25/2014 5:12:28 PM
Method	APCI tune_pos-6Dec12.m	Operator	default user
Sample Name	Por-4CHONi	Instrument / Ser#	micrOTOF-Q II 10269
Comment	A/P WU J S		

Acquisition Parameter

Source Type	APCI	Ion Polarity	Positive	Set Nebulizer	3.0 Bar
Focus	Not active	Set Capillary	4500 V	Set Dry Heater	200 °C
Scan Begin	50 m/z	Set End Plate Offset	-500 V	Set Dry Gas	4.0 l/min
Scan End	3000 m/z	Set Collision Cell RF	500.0 Vpp	Set Divert Valve	Waste

Meas. m/z	#	Formula	m/z	err [ppm]	rdb	e⁻ Conf	N-Rule
1119.5305	1	C 72 H 77 N 4 Ni O 4	1119.5293	-1.1	36.5	even	ok

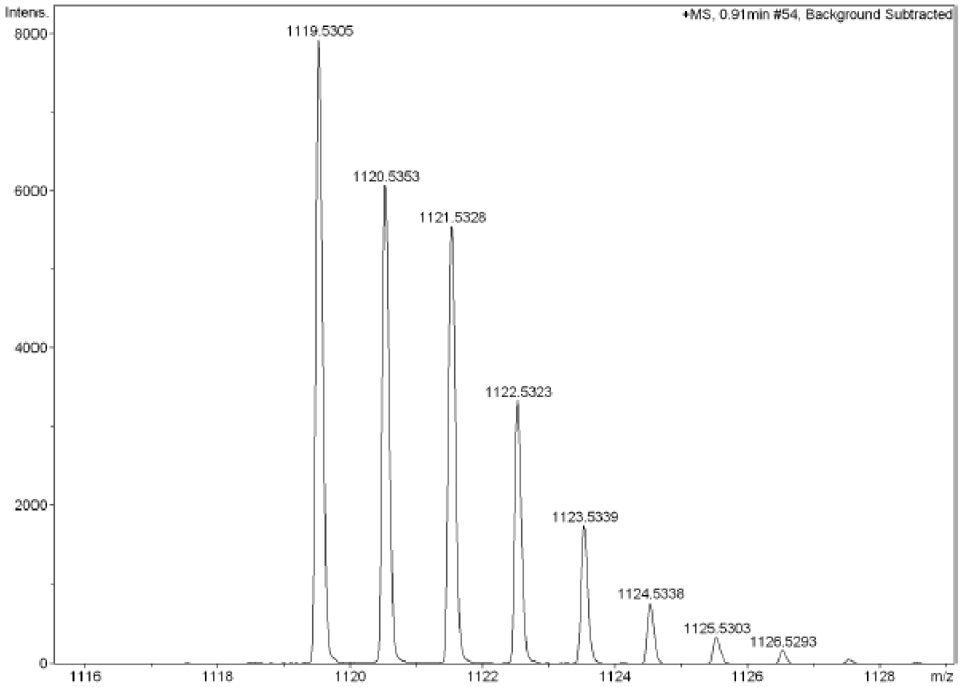

Bruker Compass DataAnalysis 4.0 printed: 6/25/2014 5:16:57 PM Page 1 of 1

Fig. S56. HR mass spectrum (HPCI-HR) of the compound **15**.

Mass Spectrum SmartFormula Report

Analysis Info

Analysis Name	D:\Data\Chemistry\2014 Sample\Jun 2014\Por-4NiOH-1.d	Acquisition Date	6/25/2014 6:28:52 PM
Method	APCI tune_pos-6Dec12.m	Operator	default user
Sample Name	Por-4NiOH	Instrument / Ser#	micrOTOF-Q II 10269
Comment	A/P WU J S		

Acquisition Parameter

Source Type	APCI	Ion Polarity	Positive	Set Nebulizer	3.0 Bar
Focus	Not active	Set Capillary	4500 V	Set Dry Heater	200 °C
Scan Begin	50 m/z	Set End Plate Offset	-500 V	Set Dry Gas	4.0 l/min
Scan End	3000 m/z	Set Collision Cell RF	500.0 Vpp	Set Divert Valve	Waste

Meas. m/z	#	Formula	m/z	err [ppm]	rdb	e¯ Conf	N-Rule
1597.8960	1	C 108 H 123 N 4 Ni O 4	1597.8892	-4.2	49.5	even	ok
1598.9018	1	C 108 H 124 N 4 Ni O 4	1598.8971	-2.9	49.0	odd	ok

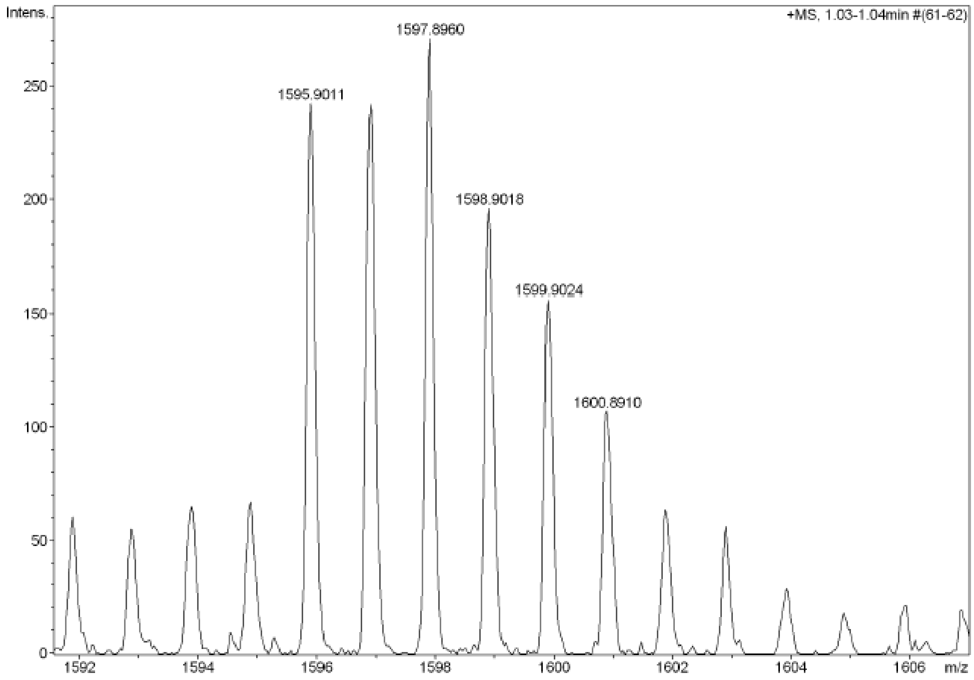

Bruker Compass DataAnalysis 4.0 printed: 6/25/2014 6:36:39 PM Page 1 of 1

Fig. S57. HR mass spectrum (HPCI-HR) of the compound **16**.

Mass Spectrum SmartFormula Report

Analysis Info

Analysis Name	D:\Data\Chemistry\2014 Sample\Jun 2014\Por-Ni-4H-1.d	Acquisition Date	6/25/2014 5:32:30 PM
Method	APCI tune_pos-6Dec12.m	Operator	default user
Sample Name	Por-Ni-4H	Instrument / Ser#	micrOTOF-Q II 10269
Comment	A/P WU J S		

Acquisition Parameter

Source Type	APCI	Ion Polarity	Positive	Set Nebulizer	3.0 Bar
Focus	Not active	Set Capillary	4500 V	Set Dry Heater	200 °C
Scan Begin	50 m/z	Set End Plate Offset	-500 V	Set Dry Gas	4.0 l/min
Scan End	3000 m/z	Set Collision Cell RF	500.0 Vpp	Set Divert Valve	Waste

Meas. m/z	#	Formula	m/z	err [ppm]	rdb	e¯ Conf	N-Rule
1527.8586	1	C 108 H 117 N 4 Ni	1527.8626	2.6	52.5	even	ok

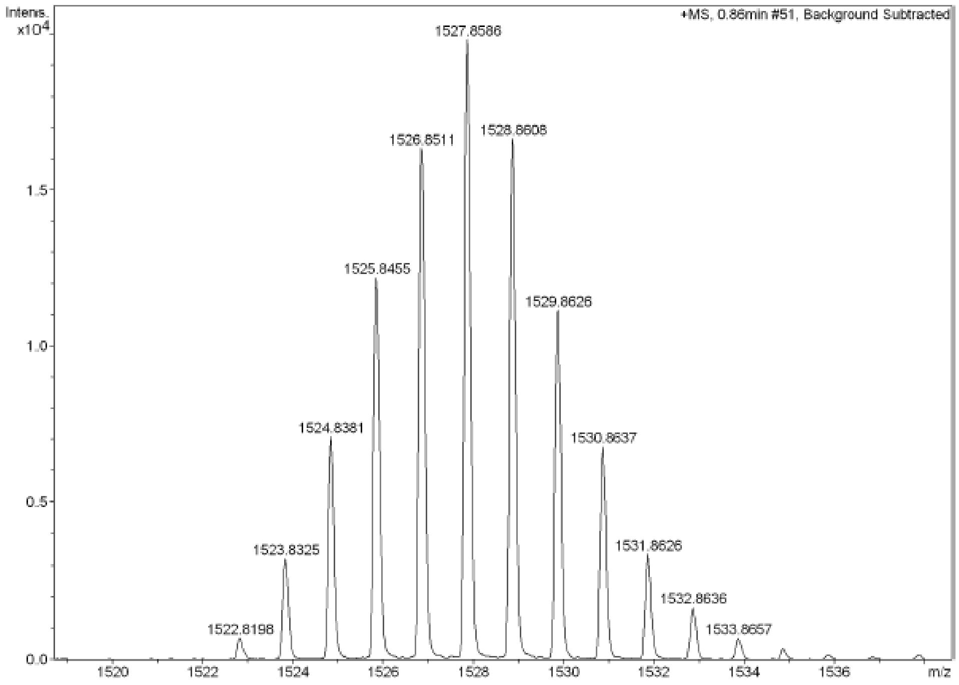

Bruker Compass DataAnalysis 4.0 printed: 6/25/2014 5:36:59 PM Page 1 of 1

Fig. S58. HR mass spectrum (HPCI-HR) of the compound **10**.

Mass Spectrum SmartFormula Report

Analysis Info

Analysis Name	D:\Data\Chemistry\2014 Sample\Jun 2014\Ni-DR.d	Acquisition Date	6/25/2014 6:49:14 PM
Method	APCI tune_pos-6Dec12.m	Operator	default user
Sample Name	Ni-DR	Instrument / Ser#	micrOTOF-Q II 10269
Comment	A/P WU J S		

Acquisition Parameter

Source Type	APCI	Ion Polarity	Positive	Set Nebulizer	3.0 Bar
Focus	Not active	Set Capillary	4500 V	Set Dry Heater	200 °C
Scan Begin	50 m/z	Set End Plate Offset	-500 V	Set Dry Gas	4.0 l/min
Scan End	3000 m/z	Set Collision Cell RF	500.0 Vpp	Set Divert Valve	Waste

Meas. m/z	#	Formula	m/z	err [ppm]	rdb	e¯ Conf	N-Rule
1522.8208	1	C 108 H 112 N 4 Ni	1522.8235	1.8	55.0	odd	ok
1523.8324	1	C 108 H 113 N 4 Ni	1523.8313	-0.7	54.5	even	ok
1524.8384	1	C 108 H 114 N 4 Ni	1524.8391	0.5	54.0	odd	ok

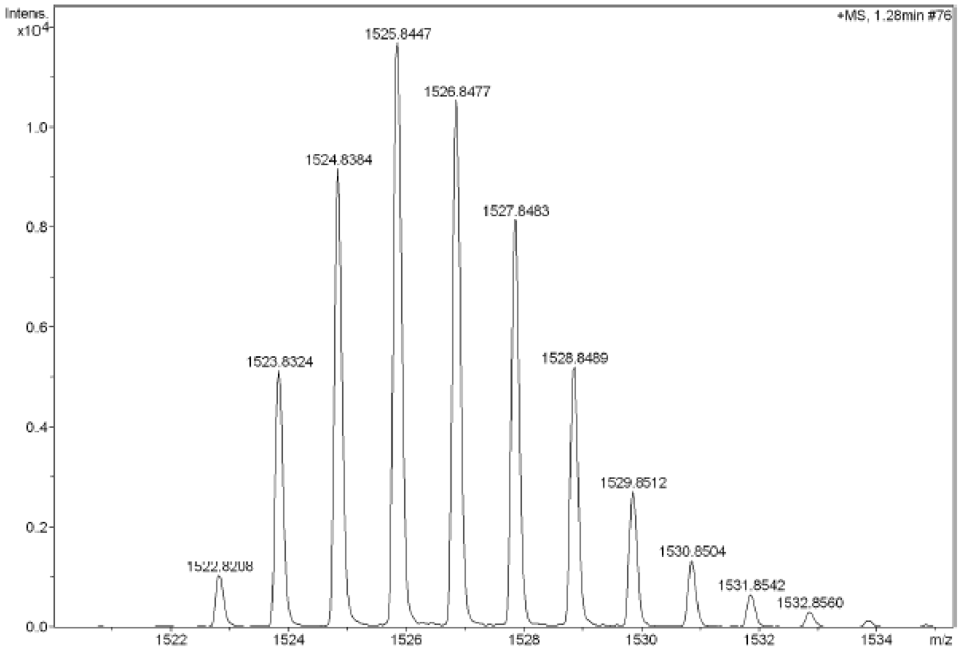

Bruker Compass DataAnalysis 4.0 printed: 6/25/2014 6:56:33 PM Page 1 of 1

Fig. S59. HR mass spectrum (APCI) of the compound **2**.

Mass Spectrum SmartFormula Report

Analysis Info

Analysis Name	D:\Data\Chemistry\2014 Sample\Jun 2014\Ni-1.d	Acquisition Date	6/25/2014 4:54:00 PM
Method	APCI tune_pos-6Dec12.m	Operator	default user
Sample Name	Ni-1	Instrument / Ser#	micrOTOF-Q II 10269
Comment	A/P WU J S		

Acquisition Parameter

Source Type	APCI	Ion Polarity	Positive	Set Nebulizer	3.0 Bar
Focus	Not active	Set Capillary	4500 V	Set Dry Heater	200 °C
Scan Begin	50 m/z	Set End Plate Offset	-500 V	Set Dry Gas	4.0 l/min
Scan End	3000 m/z	Set Collision Cell RF	500.0 Vpp	Set Divert Valve	Waste

Meas. m/z	#	Formula	m/z	err [ppm]	rdb	e¯ Conf	N-Rule
1553.8089	1	C 108 H 111 N 4 Ni O 2	1553.8055	-2.2	55.5	even	ok

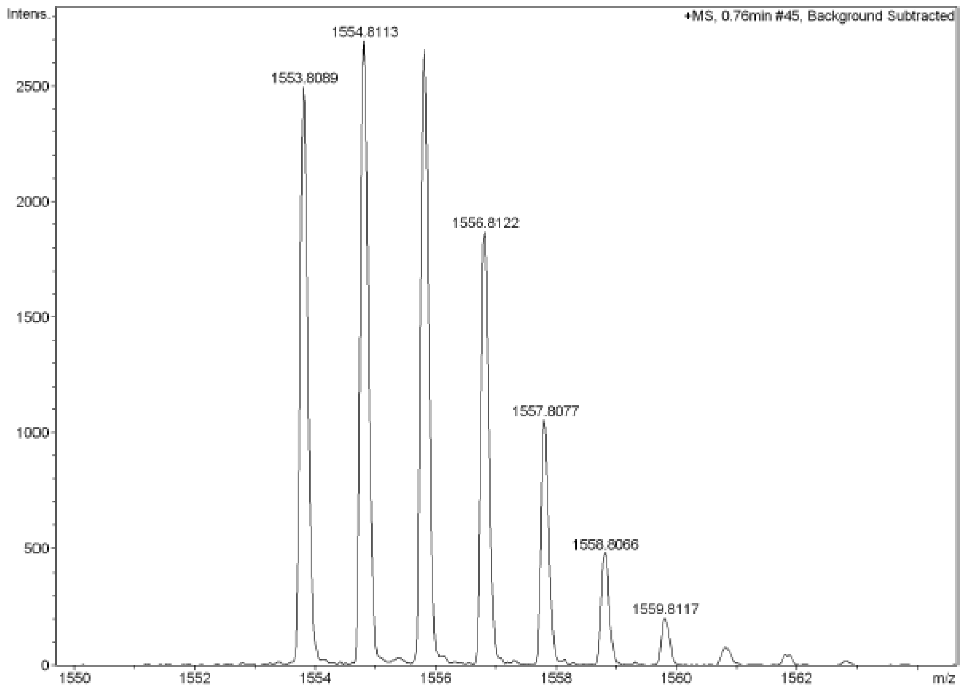

Bruker Compass DataAnalysis 4.0 printed: 6/25/2014 5:00:12 PM Page 1 of 1

Fig. S60. HR mass spectrum (APCI) of the compound **11a**.

Mass Spectrum SmartFormula Report

Analysis Info

Analysis Name	D:\Data\Chemistry\2014 Sample\Jun 2014\Ni-2.d	Acquisition Date	6/25/2014 6:13:34 PM
Method	APCI tune_pos-6Dec12.m	Operator	default user
Sample Name	Ni-2	Instrument / Ser#	micrOTOF-Q II 10269
Comment	A/P WU J S		

Acquisition Parameter

Source Type	APCI	Ion Polarity	Positive	Set Nebulizer	3.0 Bar
Focus	Not active	Set Capillary	4500 V	Set Dry Heater	200 °C
Scan Begin	50 m/z	Set End Plate Offset	-500 V	Set Dry Gas	4.0 l/min
Scan End	3000 m/z	Set Collision Cell RF	500.0 Vpp	Set Divert Valve	Waste

Meas. m/z	#	Formula	m/z	err [ppm]	rdb	e¯ Conf	N-Rule
1553.8108	1	C 108 H 111 N 4 Ni O 2	1553.8055	-3.4	55.5	even	ok

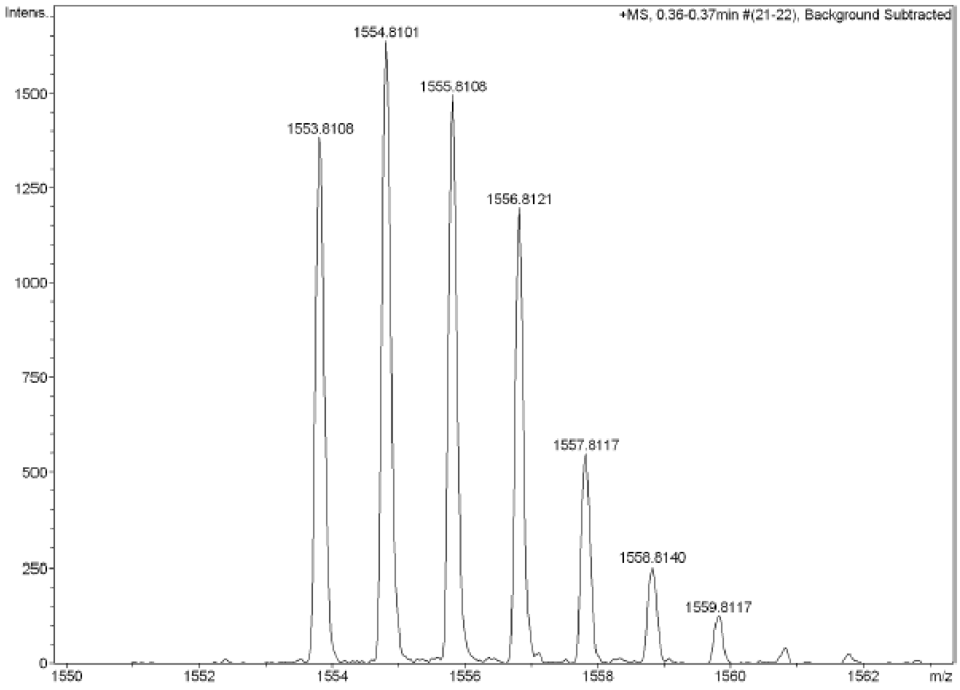

Bruker Compass DataAnalysis 4.0 printed: 6/25/2014 6:18:07 PM Page 1 of 1

Fig. S61. HR mass spectrum (APCI) of the compound **11b**.

8. Crystallographic structures of 1-H2, **11a** and **11b**

8.1 Crystallographic data for compound **1-H2**

Singlet crystal of compound **1-H2** was obtained through slow diffusion of acetonitrile to the toluene solution.

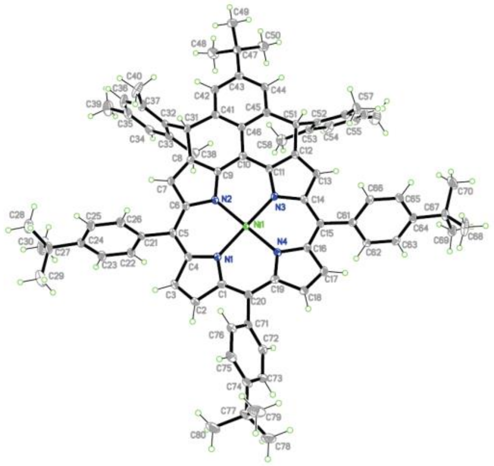

Fig. S62. X-ray crystallographic structure of **1-H2**. Solvent molecules are omitted for clarity; ellipsoids are set to 50% probability

Table S6. Crystallographic data and structure refinement for **1-H2**.

Chemical formula	$C_{85.50}H_{87}N_5Ni$	
Formula weight	1243.31	
Temperature	100(2) K	
Wavelength	0.71073 Å	
Crystal size	0.065 x 0.074 x 0.357 mm	
Crystal system	triclinic	
Space group	P -1	
Unit cell dimensions	a = 15.0498(12) Å	α = 111.901(3)°
	b = 15.6708(13) Å	β = 107.076(3)°
	c = 17.4668(15) Å	γ = 102.416(3)°
Volume	3400.2(5) $Å^3$	
Z	2	
Density (calculated)	1.214 g/cm^3	
Absorption coefficient	0.336 mm^{-1}	
F(000)	1326	
Theta range for data collection	2.12 to 25.03°	
Index ranges	-17<=h<=17, -18<=k<=18, -20<=l<=20	

Reflections collected	123595	
Independent reflections	12000 [R(int) = 0.0471]	
Coverage of independent reflections	99.9%	
Absorption correction	multi-scan	
Max. and min. transmission	0.7457 and 0.7138	
Structure solution technique	direct methods	
Structure solution program	SHELXS-97 (Sheldrick 2008)	
Refinement method	Full-matrix least-squares on F2	
Refinement program	SHELXL-2013 (Sheldrick, 2013)	
Function minimized	Σ w(Fo2 - Fc2)2	
Data / restraints / parameters	12000 / 71 / 893	
Goodness-of-fit on F2	1.034	
Δ/σmax	0.001	
Final R indices	10101 data; I>2σ(I)	R1 = 0.0441, wR2 = 0.1065
	all data	R1 = 0.0564, wR2 = 0.1134
Weighting scheme	$w=1/[\sigma^2(F_o^2)+(0.0476P)^2+4.6525P]$ where $P=(F_o^2+2F_c^2)/3$	
Largest diff. peak and hole	0.987 and -0.558 eÅ^{-3}	
R.M.S. deviation from mean	0.059 eÅ^{-3}	

Table S7. Atomic coordinates and equivalent isotropic atomic displacement parameters (Å^2), U(eq) is defined as one third of the trace of the orthogonalized U_{ij} tensor.

	x/a	y/b	z/c	U(eq)
C28	0.0717(3)	0.1176(2)	0.5142(3)	0.0339(9)
C29	0.2023(4)	0.2691(4)	0.6456(3)	0.0699(16)
C30	0.1801(3)	0.2355(3)	0.4879(3)	0.0510(11)
C28A	0.0629(14)	0.1188(6)	0.4784(12)	0.0343(12)
C29A	0.169(2)	0.233(2)	0.6415(9)	0.0677(18)
C30A	0.2191(12)	0.2693(16)	0.5351(18)	0.0515(14)
N1X	0.1472(3)	0.9441(3)	0.7059(2)	0.0608(10)
C1X	0.1163(3)	0.8688(3)	0.7017(2)	0.0350(8)
C2X	0.0778(14)	0.7745(8)	0.6972(10)	0.0449(9)
C1Y	0.0712(13)	0.8448(12)	0.7631(10)	0.0350(8)
N1Y	0.0565(14)	0.9025(12)	0.8162(11)	0.0608(10)
C2Y	0.075(8)	0.767(5)	0.689(5)	0.0449(9)
C1S	0.0687(4)	0.4886(3)	0.9686(3)	0.0606(14)

	x/a	y/b	z/c	U(eq)
C2S	0.1662(4)	0.4997(3)	0.9785(3)	0.0673(17)
C3S	0.1865(3)	0.4634(3)	0.9025(4)	0.067(2)
C4S	0.1092(5)	0.4161(3)	0.8164(3)	0.0606(14)
C5S	0.0117(4)	0.4050(3)	0.8064(3)	0.0673(17)
C6S	0.9914(3)	0.4413(4)	0.8825(4)	0.077(3)
C7S	0.0404(7)	0.5219(6)	0.0436(5)	0.071(2)
Ni1	0.72197(2)	0.59837(2)	0.59057(2)	0.01105(8)
N1	0.84069(13)	0.59747(13)	0.67243(12)	0.0136(4)
N2	0.71543(13)	0.48113(13)	0.49247(12)	0.0125(4)
N3	0.60683(13)	0.60256(13)	0.50754(12)	0.0125(4)
N4	0.72658(13)	0.71350(13)	0.68902(12)	0.0128(4)
C1	0.88805(16)	0.65437(16)	0.76519(15)	0.0156(5)
C2	0.97574(17)	0.63493(17)	0.80120(15)	0.0188(5)
C3	0.98152(17)	0.56549(17)	0.73083(15)	0.0201(5)
C4	0.89693(16)	0.54030(16)	0.65109(15)	0.0152(5)
C5	0.87040(16)	0.46122(16)	0.56729(15)	0.0146(5)
C6	0.78264(15)	0.43280(15)	0.49324(14)	0.0125(4)
C7	0.75032(16)	0.34968(16)	0.40640(14)	0.0147(5)
C8	0.66257(16)	0.34616(15)	0.35194(14)	0.0134(4)
C9	0.64245(15)	0.42762(15)	0.40510(14)	0.0124(4)
C10	0.56235(15)	0.45096(15)	0.36707(14)	0.0124(4)
C11	0.54977(15)	0.53583(15)	0.41760(14)	0.0119(4)
C12	0.47816(16)	0.56921(16)	0.37566(14)	0.0130(4)
C13	0.49333(16)	0.65931(16)	0.43955(14)	0.0143(4)
C14	0.57019(16)	0.67834(16)	0.52174(14)	0.0135(4)
C15	0.59878(15)	0.75951(15)	0.60509(14)	0.0129(4)
C16	0.66867(16)	0.77059(16)	0.68402(14)	0.0136(4)
C17	0.68694(16)	0.84174(16)	0.77298(15)	0.0162(5)
C18	0.75505(16)	0.82822(16)	0.83251(15)	0.0159(5)
C19	0.78176(16)	0.75077(16)	0.78079(14)	0.0141(4)
C20	0.85809(16)	0.72285(16)	0.81864(14)	0.0145(5)
C21	0.93504(16)	0.40085(16)	0.55911(14)	0.0142(4)
C22	0.03031(17)	0.43758(17)	0.56366(15)	0.0182(5)
C23	0.08973(17)	0.38094(17)	0.55817(16)	0.0196(5)
C24	0.05755(17)	0.28586(16)	0.54872(15)	0.0175(5)

	x/a	y/b	z/c	U(eq)
C25	0.96227(17)	0.24970(17)	0.54433(16)	0.0210(5)
C26	0.90244(17)	0.30600(17)	0.54972(16)	0.0197(5)
C27	0.12659(16)	0.22673(16)	0.54777(16)	0.0254(6)
C31	0.59008(16)	0.27123(16)	0.25696(14)	0.0147(5)
C32	0.63952(16)	0.22769(16)	0.19499(14)	0.0159(5)
C33	0.70250(17)	0.28870(17)	0.17563(15)	0.0196(5)
C34	0.74374(17)	0.24662(19)	0.11612(16)	0.0231(5)
C35	0.72351(19)	0.1459(2)	0.07426(16)	0.0261(6)
C36	0.6596(2)	0.08669(19)	0.09310(17)	0.0282(6)
C37	0.61683(18)	0.12533(18)	0.15250(16)	0.0220(5)
C38	0.7253(2)	0.39841(18)	0.21494(18)	0.0295(6)
C39	0.7696(2)	0.1021(2)	0.0104(2)	0.0401(7)
C40	0.5451(2)	0.05551(18)	0.16697(19)	0.0341(7)
C41	0.51363(16)	0.30911(16)	0.21539(15)	0.0144(4)
C42	0.45886(16)	0.25924(16)	0.12236(15)	0.0158(5)
C43	0.38818(16)	0.28879(16)	0.07799(15)	0.0149(5)
C44	0.37071(16)	0.36862(16)	0.13234(15)	0.0154(5)
C45	0.42448(16)	0.42094(16)	0.22586(15)	0.0143(4)
C46	0.49805(16)	0.39203(15)	0.26907(14)	0.0134(4)
C47	0.33520(17)	0.23535(17)	0.97437(15)	0.0186(5)
C48	0.40697(19)	0.2036(2)	0.93327(17)	0.0281(6)
C49	0.24595(18)	0.14289(18)	0.94384(16)	0.0235(5)
C50	0.29868(19)	0.30219(19)	0.93778(16)	0.0251(5)
C51	0.40304(16)	0.50985(16)	0.27893(14)	0.0140(4)
C52	0.38848(16)	0.57819(16)	0.23627(14)	0.0150(5)
C53	0.45263(17)	0.60808(17)	0.19920(15)	0.0193(5)
C54	0.4354(2)	0.66953(18)	0.16070(17)	0.0272(6)
C55	0.3592(2)	0.70578(19)	0.16051(17)	0.0286(6)
C56	0.30053(19)	0.68064(18)	0.20229(16)	0.0251(6)
C57	0.31315(17)	0.61730(17)	0.23993(15)	0.0189(5)
C58	0.54242(18)	0.57902(19)	0.20127(18)	0.0261(6)
C59	0.3422(3)	0.7728(2)	0.1183(2)	0.0449(8)
C60	0.24579(18)	0.59464(18)	0.28475(17)	0.0242(5)
C61	0.54810(16)	0.83238(16)	0.60920(14)	0.0138(4)
C62	0.60123(16)	0.93188(16)	0.64106(15)	0.0169(5)

	x/a	y/b	z/c	U(eq)
C63	0.55257(17)	0.99764(16)	0.63854(15)	0.0181(5)
C64	0.44912(17)	0.96715(16)	0.60519(15)	0.0164(5)
C65	0.39665(17)	0.86801(17)	0.57715(15)	0.0173(5)
C66	0.44443(16)	0.80214(16)	0.57826(14)	0.0152(5)
C67	0.39320(18)	0.03737(18)	0.59971(16)	0.0218(5)
C68	0.3668(2)	0.0711(2)	0.6821(2)	0.0378(7)
C69	0.45626(19)	0.12848(17)	0.60025(17)	0.0243(5)
C70	0.29743(19)	0.9843(2)	0.5120(2)	0.0328(6)
C71	0.91137(16)	0.76734(16)	0.91943(15)	0.0158(5)
C72	0.96975(16)	0.86711(17)	0.97336(15)	0.0181(5)
C73	0.01851(17)	0.90695(18)	0.06699(15)	0.0198(5)
C74	0.01073(16)	0.84885(18)	0.11016(15)	0.0196(5)
C75	0.95455(17)	0.74822(18)	0.05528(16)	0.0212(5)
C76	0.90592(16)	0.70797(17)	0.96227(15)	0.0186(5)
C77	0.06279(18)	0.8907(2)	0.21316(16)	0.0260(6)
C78	0.1093(2)	0.0037(2)	0.26199(17)	0.0331(6)
C79	0.9874(2)	0.8606(2)	0.25028(18)	0.0376(7)
C80	0.1451(2)	0.8492(2)	0.23380(18)	0.0351(7)

Table S8. Band length **(Å)** and Bond angles (°) for **1-H2**

C28-C27	1.520(3)	C29-C27	1.530(3)
C30-C27	1.522(3)	C28A-C27	1.530(5)
C29A-C27	1.529(5)	C30A-C27	1.524(5)
N1X-C1X	1.137(5)	C1X-C2X	1.431(18)
C1Y-N1Y	1.145(10)	C1Y-C2Y	1.43(2)
C1S-C2S	1.39	C1S-C6S	1.39
C1S-C7S	1.444(9)	C2S-C3S	1.39
C3S-C4S	1.39	C4S-C5S	1.39
C5S-C6S	1.39	Ni1-N3	1.9377(17)
Ni1-N1	1.9397(18)	Ni1-N4	1.9439(18)
Ni1-N2	1.9482(18)	N1-C1	1.378(3)
N1-C4	1.386(3)	N2-C9	1.380(3)
N2-C6	1.388(3)	N3-C11	1.376(3)
N3-C14	1.385(3)	N4-C19	1.382(3)
N4-C16	1.384(3)	C1-C20	1.391(3)

C1-C2	1.435(3)	C2-C3	1.345(3)
C3-C4	1.431(3)	C4-C5	1.389(3)
C5-C6	1.389(3)	C5-C21	1.496(3)
C6-C7	1.433(3)	C7-C8	1.358(3)
C8-C9	1.425(3)	C8-C31	1.501(3)
C9-C10	1.389(3)	C10-C11	1.384(3)
C10-C46	1.477(3)	C11-C12	1.427(3)
C12-C13	1.352(3)	C12-C51	1.501(3)
C13-C14	1.430(3)	C14-C15	1.392(3)
C15-C16	1.389(3)	C15-C61	1.495(3)
C16-C17	1.435(3)	C17-C18	1.345(3)
C18-C19	1.434(3)	C19-C20	1.391(3)
C20-C71	1.498(3)	C21-C26	1.391(3)
C21-C22	1.392(3)	C22-C23	1.387(3)
C23-C24	1.393(3)	C24-C25	1.392(3)
C24-C27	1.533(3)	C25-C26	1.388(3)
C31-C41	1.526(3)	C31-C32	1.527(3)
C32-C33	1.402(3)	C32-C37	1.403(3)
C33-C34	1.394(3)	C33-C38	1.507(3)
C34-C35	1.385(4)	C35-C36	1.387(4)
C35-C39	1.512(3)	C36-C37	1.396(3)
C37-C40	1.512(4)	C41-C42	1.386(3)
C41-C46	1.405(3)	C42-C43	1.394(3)
C43-C44	1.392(3)	C43-C47	1.532(3)
C44-C45	1.391(3)	C45-C46	1.408(3)
C45-C51	1.520(3)	C47-C50	1.532(3)
C47-C48	1.533(3)	C47-C49	1.534(3)
C51-C52	1.533(3)	C52-C53	1.403(3)
C52-C57	1.406(3)	C53-C54	1.391(3)
C53-C58	1.511(3)	C54-C55	1.385(4)
C55-C56	1.383(4)	C55-C59	1.516(3)
C56-C57	1.396(3)	C57-C60	1.512(3)
C61-C62	1.391(3)	C61-C66	1.395(3)
C62-C63	1.393(3)	C63-C64	1.390(3)
C64-C65	1.398(3)	C64-C67	1.536(3)
C65-C66	1.382(3)	C67-C69	1.530(3)
C67-C70	1.534(4)	C67-C68	1.539(3)

C71-C72	1.389(3)	C71-C76	1.398(3)
C72-C73	1.393(3)	C73-C74	1.389(3)
C74-C75	1.394(3)	C74-C77	1.534(3)
C75-C76	1.383(3)	C77-C78	1.531(4)
C77-C80	1.532(4)	C77-C79	1.536(4)

N1X-C1X-C2X	179.5(9)	N1Y-C1Y-C2Y	172.(5)
C2S-C1S-C6S	120.0	C2S-C1S-C7S	123.8(5)
C6S-C1S-C7S	116.2(5)	C3S-C2S-C1S	120.0
C4S-C3S-C2S	120.0	C3S-C4S-C5S	120.0
C6S-C5S-C4S	120.0	C5S-C6S-C1S	120.0
N3-Ni1-N1	177.67(8)	N3-Ni1-N4	89.79(7)
N1-Ni1-N4	90.30(7)	N3-Ni1-N2	90.21(7)
N1-Ni1-N2	89.75(7)	N4-Ni1-N2	178.75(7)
C1-N1-C4	104.68(17)	C1-N1-Ni1	127.33(14)
C4-N1-Ni1	127.99(15)	C9-N2-C6	103.97(17)
C9-N2-Ni1	127.43(14)	C6-N2-Ni1	128.60(14)
C11-N3-C14	104.02(17)	C11-N3-Ni1	127.61(14)
C14-N3-Ni1	127.99(14)	C19-N4-C16	104.40(17)
C19-N4-Ni1	127.38(14)	C16-N4-Ni1	127.95(14)
N1-C1-C20	126.1(2)	N1-C1-C2	110.65(19)
C20-C1-C2	123.2(2)	C3-C2-C1	107.0(2)
C2-C3-C4	107.2(2)	N1-C4-C5	125.8(2)
N1-C4-C3	110.40(19)	C5-C4-C3	123.4(2)
C4-C5-C6	122.0(2)	C4-C5-C21	118.46(19)
C6-C5-C21	119.40(19)	N2-C6-C5	125.08(19)
N2-C6-C7	110.87(18)	C5-C6-C7	124.05(19)
C8-C7-C6	106.85(19)	C7-C8-C9	106.60(19)
C7-C8-C31	131.04(19)	C9-C8-C31	122.17(18)
N2-C9-C10	126.74(19)	N2-C9-C8	111.70(18)
C10-C9-C8	121.34(19)	C11-C10-C9	120.4(2)
C11-C10-C46	119.55(19)	C9-C10-C46	119.56(19)
N3-C11-C10	127.26(19)	N3-C11-C12	111.58(18)
C10-C11-C12	120.84(19)	C13-C12-C11	106.47(19)
C13-C12-C51	130.13(19)	C11-C12-C51	123.40(19)
C12-C13-C14	107.02(19)	N3-C14-C15	125.41(19)
N3-C14-C13	110.79(18)	C15-C14-C13	123.69(19)

C16-C15-C14	121.31(19)	C16-C15-C61	120.05(19)
C14-C15-C61	118.54(19)	N4-C16-C15	125.86(19)
N4-C16-C17	110.66(18)	C15-C16-C17	123.43(19)
C18-C17-C16	107.09(19)	C17-C18-C19	107.01(19)
N4-C19-C20	125.2(2)	N4-C19-C18	110.79(18)
C20-C19-C18	123.9(2)	C19-C20-C1	121.7(2)
C19-C20-C71	120.24(19)	C1-C20-C71	118.02(19)
C26-C21-C22	117.5(2)	C26-C21-C5	120.39(19)
C22-C21-C5	122.1(2)	C23-C22-C21	120.9(2)
C22-C23-C24	121.9(2)	C25-C24-C23	116.8(2)
C25-C24-C27	122.2(2)	C23-C24-C27	120.9(2)
C26-C25-C24	121.5(2)	C25-C26-C21	121.3(2)
C28-C27-C30	108.1(3)	C30A-C27-C29A	103.9(16)
C30A-C27-C28A	114.2(13)	C29A-C27-C28A	108.4(14)
C28-C27-C29	107.4(3)	C30-C27-C29	110.3(3)
C28-C27-C24	113.1(2)	C30-C27-C24	110.5(2)
C30A-C27-C24	114.3(9)	C29A-C27-C24	107.9(11)
C28A-C27-C24	107.8(9)	C29-C27-C24	107.3(2)
C8-C31-C41	112.26(17)	C8-C31-C32	114.00(18)
C41-C31-C32	112.49(18)	C33-C32-C37	119.7(2)
C33-C32-C31	120.30(19)	C37-C32-C31	119.9(2)
C34-C33-C32	119.2(2)	C34-C33-C38	118.4(2)
C32-C33-C38	122.4(2)	C35-C34-C33	122.1(2)
C34-C35-C36	117.7(2)	C34-C35-C39	121.2(2)
C36-C35-C39	121.1(2)	C35-C36-C37	122.3(2)
C36-C37-C32	118.9(2)	C36-C37-C40	119.1(2)
C32-C37-C40	121.9(2)	C42-C41-C46	120.2(2)
C42-C41-C31	118.22(19)	C46-C41-C31	121.59(19)
C41-C42-C43	122.5(2)	C44-C43-C42	116.6(2)
C44-C43-C47	123.13(19)	C42-C43-C47	120.22(19)
C45-C44-C43	122.5(2)	C44-C45-C46	119.9(2)
C44-C45-C51	118.61(19)	C46-C45-C51	121.49(19)
C41-C46-C45	118.16(19)	C41-C46-C10	120.49(19)
C45-C46-C10	121.21(19)	C43-C47-C50	111.60(19)
C43-C47-C48	110.16(18)	C50-C47-C48	107.6(2)
C43-C47-C49	109.28(19)	C50-C47-C49	109.44(19)
C48-C47-C49	108.7(2)	C12-C51-C45	112.59(17)

C12-C51-C52	110.01(17)	C45-C51-C52	115.90(18)
C53-C52-C57	119.1(2)	C53-C52-C51	121.70(19)
C57-C52-C51	119.1(2)	C54-C53-C52	119.3(2)
C54-C53-C58	117.6(2)	C52-C53-C58	123.1(2)
C55-C54-C53	122.4(2)	C56-C55-C54	117.6(2)
C56-C55-C59	121.0(2)	C54-C55-C59	121.4(3)
C55-C56-C57	122.2(2)	C56-C57-C52	119.3(2)
C56-C57-C60	118.3(2)	C52-C57-C60	122.4(2)
C62-C61-C66	117.2(2)	C62-C61-C15	121.88(19)
C66-C61-C15	120.86(19)	C61-C62-C63	121.3(2)
C64-C63-C62	121.6(2)	C63-C64-C65	116.7(2)
C63-C64-C67	122.8(2)	C65-C64-C67	120.5(2)
C66-C65-C64	121.9(2)	C65-C66-C61	121.2(2)
C69-C67-C70	107.5(2)	C69-C67-C64	112.04(19)
C70-C67-C64	110.2(2)	C69-C67-C68	108.9(2)
C70-C67-C68	109.8(2)	C64-C67-C68	108.29(19)
C72-C71-C76	117.6(2)	C72-C71-C20	122.1(2)
C76-C71-C20	120.3(2)	C71-C72-C73	121.1(2)
C74-C73-C72	121.5(2)	C73-C74-C75	117.0(2)
C73-C74-C77	122.7(2)	C75-C74-C77	120.2(2)
C76-C75-C74	121.9(2)	C75-C76-C71	120.9(2)
C78-C77-C80	109.1(2)	C78-C77-C74	112.1(2)
C80-C77-C74	108.3(2)	C78-C77-C79	107.4(2)
C80-C77-C79	109.7(2)	C74-C77-C79	110.3(2)

Table S9. Anisotropic atomic displacement parameters (Å^2) for **1-H2**, The anisotropic atomic isplacement factor exponent takes the form: $-2\pi^2[h^2 a^{*2} U_{11} + ... + 2 h k a^* b^* U_{12}]$

	**U_{11}**	**U_{22}**	**U_{33}**	**U_{23}**	**U_{13}**	**U_{12}**
C28	0.0333(14)	0.0285(13)	0.0481(18)	0.0190(12)	0.0199(14)	0.0199(11)
C29	0.061(2)	0.063(2)	0.072(2)	0.0233(15)	0.0042(14)	0.0461(19)
C30	0.0528(18)	0.0452(18)	0.077(2)	0.0308(15)	0.0446(16)	0.0294(14)
C28A	0.0336(16)	0.0293(16)	0.047(2)	0.0185(14)	0.0191(16)	0.0194(14)
C29A	0.061(2)	0.062(2)	0.071(2)	0.0253(16)	0.0073(15)	0.044(2)
C30A	0.053(2)	0.046(2)	0.075(2)	0.0299(16)	0.0420(17)	0.0284(15)
N1X	0.068(2)	0.046(2)	0.048(2)	0.0171(17)	0.0162(18)	0.0043(18)
C1X	0.0343(18)	0.040(2)	0.0235(17)	0.0122(15)	0.0078(14)	0.0103(16)
C2X	0.053(2)	0.047(2)	0.037(3)	0.023(2)	0.015(2)	0.022(2)

	U_{11}	U_{22}	U_{33}	U_{23}	U_{13}	U_{12}
C1Y	0.0343(18)	0.040(2)	0.0235(17)	0.0122(15)	0.0078(14)	0.0103(16)
N1Y	0.068(2)	0.046(2)	0.048(2)	0.0171(17)	0.0162(18)	0.0043(18)
C2Y	0.053(2)	0.047(2)	0.037(3)	0.023(2)	0.015(2)	0.022(2)
C1S	0.085(4)	0.042(3)	0.088(4)	0.048(3)	0.044(3)	0.037(3)
C2S	0.094(5)	0.025(2)	0.053(3)	0.023(2)	-0.002(3)	0.006(3)
C3S	0.082(6)	0.061(5)	0.132(8)	0.086(5)	0.069(6)	0.051(4)
C4S	0.085(4)	0.042(3)	0.088(4)	0.048(3)	0.044(3)	0.037(3)
C5S	0.094(5)	0.025(2)	0.053(3)	0.023(2)	-0.002(3)	0.006(3)
C6S	0.082(6)	0.047(5)	0.100(7)	0.046(5)	0.021(6)	0.022(4)
C7S	0.094(6)	0.049(4)	0.051(4)	0.008(4)	0.018(4)	0.037(4)
Ni1	0.01113(14)	0.01162(14)	0.01055(15)	0.00472(11)	0.00376(11)	0.00623(11)
N1	0.0134(9)	0.0133(9)	0.0133(9)	0.0056(8)	0.0044(8)	0.0062(8)
N2	0.0119(9)	0.0131(9)	0.0143(9)	0.0072(8)	0.0057(7)	0.0058(7)
N3	0.0133(9)	0.0122(9)	0.0127(9)	0.0052(8)	0.0060(8)	0.0060(7)
N4	0.0123(9)	0.0145(9)	0.0116(9)	0.0068(8)	0.0037(7)	0.0053(7)
C1	0.0156(11)	0.0139(11)	0.0137(11)	0.0056(9)	0.0029(9)	0.0047(9)
C2	0.0158(11)	0.0200(12)	0.0151(11)	0.0057(10)	0.0014(9)	0.0083(10)
C3	0.0160(11)	0.0217(12)	0.0200(12)	0.0075(10)	0.0039(10)	0.0114(10)
C4	0.0138(11)	0.0169(11)	0.0167(11)	0.0083(9)	0.0060(9)	0.0082(9)
C5	0.0141(11)	0.0149(11)	0.0183(11)	0.0095(9)	0.0077(9)	0.0073(9)
C6	0.0130(10)	0.0120(10)	0.0159(11)	0.0079(9)	0.0075(9)	0.0060(9)
C7	0.0158(11)	0.0144(11)	0.0159(11)	0.0067(9)	0.0076(9)	0.0085(9)
C8	0.0149(11)	0.0125(11)	0.0133(11)	0.0059(9)	0.0062(9)	0.0058(9)
C9	0.0131(11)	0.0122(10)	0.0138(11)	0.0068(9)	0.0067(9)	0.0051(9)
C10	0.0123(10)	0.0122(10)	0.0148(11)	0.0076(9)	0.0070(9)	0.0042(9)
C11	0.0115(10)	0.0135(11)	0.0125(11)	0.0067(9)	0.0059(9)	0.0049(9)
C12	0.0141(11)	0.0149(11)	0.0128(11)	0.0074(9)	0.0067(9)	0.0068(9)
C13	0.0153(11)	0.0156(11)	0.0147(11)	0.0081(9)	0.0061(9)	0.0086(9)
C14	0.0127(11)	0.0151(11)	0.0156(11)	0.0086(9)	0.0064(9)	0.0070(9)
C15	0.0123(10)	0.0130(11)	0.0150(11)	0.0069(9)	0.0068(9)	0.0051(9)
C16	0.0136(11)	0.0140(11)	0.0151(11)	0.0069(9)	0.0072(9)	0.0065(9)
C17	0.0179(11)	0.0163(11)	0.0165(11)	0.0068(9)	0.0084(9)	0.0099(9)
C18	0.0171(11)	0.0184(11)	0.0115(11)	0.0052(9)	0.0061(9)	0.0078(9)
C19	0.0151(11)	0.0142(11)	0.0134(11)	0.0069(9)	0.0062(9)	0.0046(9)
C20	0.0142(11)	0.0145(11)	0.0151(11)	0.0076(9)	0.0054(9)	0.0056(9)
C21	0.0144(11)	0.0159(11)	0.0113(11)	0.0053(9)	0.0035(9)	0.0079(9)

	U_{11}	U_{22}	U_{33}	U_{23}	U_{13}	U_{12}
C22	0.0175(12)	0.0155(11)	0.0221(12)	0.0097(10)	0.0076(10)	0.0063(9)
C23	0.0139(11)	0.0210(12)	0.0271(13)	0.0117(10)	0.0103(10)	0.0083(10)
C24	0.0179(11)	0.0183(12)	0.0172(12)	0.0076(10)	0.0073(10)	0.0094(10)
C25	0.0199(12)	0.0155(11)	0.0294(13)	0.0118(10)	0.0097(10)	0.0082(10)
C26	0.0141(11)	0.0189(12)	0.0259(13)	0.0099(10)	0.0081(10)	0.0068(10)
C27	0.0185(12)	0.0222(13)	0.0407(15)	0.0158(12)	0.0130(11)	0.0134(10)
C31	0.0168(11)	0.0133(11)	0.0138(11)	0.0053(9)	0.0063(9)	0.0077(9)
C32	0.0148(11)	0.0191(11)	0.0126(11)	0.0055(9)	0.0036(9)	0.0107(9)
C33	0.0161(11)	0.0244(13)	0.0159(11)	0.0081(10)	0.0039(9)	0.0095(10)
C34	0.0183(12)	0.0356(14)	0.0194(12)	0.0146(11)	0.0084(10)	0.0126(11)
C35	0.0299(14)	0.0379(15)	0.0205(13)	0.0153(12)	0.0129(11)	0.0238(12)
C36	0.0450(16)	0.0258(13)	0.0250(13)	0.0122(11)	0.0191(12)	0.0257(12)
C37	0.0298(13)	0.0218(12)	0.0188(12)	0.0095(10)	0.0113(11)	0.0158(11)
C38	0.0304(14)	0.0230(13)	0.0307(14)	0.0092(12)	0.0157(12)	0.0035(11)
C39	0.0530(19)	0.0514(19)	0.0410(17)	0.0255(15)	0.0341(15)	0.0365(16)
C40	0.0604(19)	0.0165(13)	0.0356(16)	0.0106(12)	0.0320(15)	0.0183(13)
C41	0.0130(11)	0.0140(11)	0.0164(11)	0.0074(9)	0.0063(9)	0.0042(9)
C42	0.0157(11)	0.0141(11)	0.0157(11)	0.0045(9)	0.0072(9)	0.0056(9)
C43	0.0139(11)	0.0158(11)	0.0143(11)	0.0067(9)	0.0064(9)	0.0039(9)
C44	0.0137(11)	0.0161(11)	0.0167(11)	0.0086(9)	0.0048(9)	0.0063(9)
C45	0.0137(11)	0.0141(11)	0.0161(11)	0.0071(9)	0.0069(9)	0.0056(9)
C46	0.0130(11)	0.0127(11)	0.0144(11)	0.0063(9)	0.0061(9)	0.0038(9)
C47	0.0196(12)	0.0205(12)	0.0141(11)	0.0060(10)	0.0060(10)	0.0090(10)
C48	0.0297(14)	0.0402(15)	0.0174(12)	0.0120(12)	0.0125(11)	0.0167(12)
C49	0.0223(13)	0.0230(13)	0.0167(12)	0.0050(10)	0.0041(10)	0.0061(10)
C50	0.0288(14)	0.0298(14)	0.0151(12)	0.0107(11)	0.0060(10)	0.0115(11)
C51	0.0127(11)	0.0149(11)	0.0145(11)	0.0067(9)	0.0049(9)	0.0068(9)
C52	0.0162(11)	0.0129(11)	0.0103(10)	0.0030(9)	0.0015(9)	0.0053(9)
C53	0.0230(12)	0.0171(11)	0.0147(11)	0.0055(10)	0.0066(10)	0.0068(10)
C54	0.0406(15)	0.0255(13)	0.0223(13)	0.0135(11)	0.0173(12)	0.0137(12)
C55	0.0435(16)	0.0273(14)	0.0219(13)	0.0158(11)	0.0121(12)	0.0191(12)
C56	0.0300(14)	0.0257(13)	0.0222(13)	0.0115(11)	0.0077(11)	0.0183(11)
C57	0.0194(12)	0.0176(12)	0.0143(11)	0.0052(10)	0.0024(9)	0.0075(10)
C58	0.0253(13)	0.0272(13)	0.0327(14)	0.0171(12)	0.0169(12)	0.0095(11)
C59	0.069(2)	0.0514(19)	0.0420(18)	0.0364(16)	0.0273(16)	0.0380(17)
C60	0.0200(12)	0.0233(13)	0.0327(14)	0.0131(11)	0.0114(11)	0.0132(10)

	U_{11}	U_{22}	U_{33}	U_{23}	U_{13}	U_{12}
C61	0.0172(11)	0.0165(11)	0.0086(10)	0.0058(9)	0.0050(9)	0.0082(9)
C62	0.0138(11)	0.0165(11)	0.0158(11)	0.0049(9)	0.0033(9)	0.0059(9)
C63	0.0205(12)	0.0121(11)	0.0188(12)	0.0051(9)	0.0069(10)	0.0063(9)
C64	0.0220(12)	0.0189(12)	0.0133(11)	0.0080(9)	0.0094(9)	0.0125(10)
C65	0.0140(11)	0.0211(12)	0.0174(11)	0.0083(10)	0.0067(9)	0.0085(10)
C66	0.0175(11)	0.0130(11)	0.0155(11)	0.0064(9)	0.0078(9)	0.0054(9)
C67	0.0263(13)	0.0243(13)	0.0252(13)	0.0147(11)	0.0138(11)	0.0179(11)
C68	0.0587(19)	0.0431(17)	0.0496(18)	0.0320(15)	0.0409(16)	0.0415(16)
C69	0.0327(14)	0.0210(12)	0.0244(13)	0.0126(11)	0.0112(11)	0.0163(11)
C70	0.0240(14)	0.0341(15)	0.0462(17)	0.0258(14)	0.0081(12)	0.0166(12)
C71	0.0132(11)	0.0211(12)	0.0150(11)	0.0084(10)	0.0057(9)	0.0101(9)
C72	0.0178(12)	0.0190(12)	0.0187(12)	0.0098(10)	0.0066(10)	0.0087(10)
C73	0.0170(12)	0.0216(12)	0.0158(12)	0.0058(10)	0.0039(10)	0.0077(10)
C74	0.0135(11)	0.0294(13)	0.0164(12)	0.0102(10)	0.0053(9)	0.0108(10)
C75	0.0193(12)	0.0294(13)	0.0201(12)	0.0162(11)	0.0066(10)	0.0115(10)
C76	0.0158(11)	0.0197(12)	0.0173(12)	0.0080(10)	0.0039(9)	0.0067(10)
C77	0.0193(12)	0.0388(15)	0.0160(12)	0.0121(11)	0.0040(10)	0.0098(11)
C78	0.0290(14)	0.0407(16)	0.0149(13)	0.0060(12)	0.0019(11)	0.0095(12)
C79	0.0326(15)	0.0553(19)	0.0174(13)	0.0150(13)	0.0083(12)	0.0087(14)
C80	0.0283(14)	0.0468(17)	0.0215(14)	0.0141(13)	0.0000(11)	0.0159(13)

Table S10. Hydrogen atomic coordinates and isotropic atomic displacement parameters (Å^2) for **1-H2**

	x/a	y/b	z/c	U(eq)
H28A	1.0409	0.1100	0.5546	0.051
H28B	1.1191	0.0834	0.5134	0.051
H28C	1.0198	0.0890	0.4528	0.051
H29A	1.2406	0.3391	0.6686	0.105
H29B	1.2477	0.2329	0.6475	0.105
H29C	1.1668	0.2625	0.6832	0.105
H30A	1.1308	0.2086	0.4256	0.076
H30B	1.2248	0.1984	0.4889	0.076
H30C	1.2192	0.3052	0.5105	0.076
H28D	1.0114	0.0936	0.4964	0.051
H28E	1.1055	0.0794	0.4758	0.051
H28F	1.0311	0.1143	0.4187	0.051
H29D	1.1944	0.3018	0.6881	0.102

	x/a	y/b	z/c	U(eq)
H29E	1.2231	0.2073	0.6467	0.102
H29F	1.1159	0.1931	0.6496	0.102
H30D	1.1987	0.2692	0.4764	0.077
H30E	1.2603	0.2289	0.5372	0.077
H30F	1.2577	0.3372	0.5837	0.077
H2X1	0.0495	0.7228	0.6342	0.067
H2X2	0.0257	0.7733	0.7202	0.067
H2X3	0.1319	0.7626	0.7342	0.067
H2Y1	0.0116	0.7382	0.6362	0.067
H2Y2	0.0868	0.7164	0.7064	0.067
H2Y3	0.1296	0.7943	0.6753	0.067
H2S	0.2190	0.5321	1.0374	0.081
H3S	0.2531	0.4710	0.9093	0.08
H4S	0.1231	0.3913	0.7644	0.073
H5S	-0.0411	0.3726	0.7476	0.081
H6S	-0.0752	0.4337	0.8757	0.093
H7S1	0.0841	0.5160	1.0939	0.107
H7S2	-0.0291	0.4812	1.0245	0.107
H7S3	0.0469	0.5911	1.0633	0.107
H2	1.0211	0.6653	0.8630	0.023
H3	1.0323	0.5382	0.7335	0.024
H7	0.7839	0.3055	0.3901	0.018
H13	0.4594	0.7019	0.4314	0.017
H17	0.6566	0.8894	0.7872	0.019
H18	0.7806	0.8633	0.8967	0.019
H22	1.0550	0.5024	0.5706	0.022
H23	1.1542	0.4078	0.5609	0.024
H25	0.9376	0.1850	0.5375	0.025
H26	0.8380	0.2792	0.5469	0.024
H31	0.5512	0.2148	0.2621	0.018
H34	0.7871	0.2884	0.1039	0.028
H36	0.6445	0.0174	0.0646	0.034
H38A	0.6628	0.4105	0.1992	0.044
H38B	0.7672	0.4265	0.1902	0.044
H38C	0.7609	0.4296	0.2810	0.044
H39A	0.8165	0.0761	0.0375	0.06

	x/a	y/b	z/c	U(eq)
H39B	0.8053	0.1534	-0.0006	0.06
H39C	0.7169	0.0484	-0.0471	0.06
H40A	0.5399	-0.0123	0.1322	0.051
H40B	0.4791	0.0602	0.1466	0.051
H40C	0.5697	0.0734	0.2315	0.051
H42	0.4699	0.2028	0.0875	0.019
H44	0.3201	0.3881	0.1045	0.018
H48A	0.4678	0.2610	-0.0422	0.042
H48B	0.3748	0.1761	-0.1327	0.042
H48C	0.4242	0.1533	-0.0515	0.042
H49A	0.2149	0.1059	-0.1225	0.035
H49B	0.1970	0.1626	-0.0342	0.035
H49C	0.2690	0.1011	-0.0313	0.035
H50A	0.3551	0.3630	-0.0399	0.038
H50B	0.2479	0.3188	-0.0416	0.038
H50C	0.2698	0.2676	-0.1286	0.038
H51	0.3380	0.4824	0.2812	0.017
H54	0.4775	0.6873	0.1335	0.033
H56	0.2499	0.7074	0.2055	0.03
H58A	0.5735	0.5781	0.2586	0.039
H58B	0.5907	0.6270	0.1959	0.039
H58C	0.5212	0.5132	0.1507	0.039
H59A	0.3742	0.8419	0.1656	0.067
H59B	0.2704	0.7570	0.0883	0.067
H59C	0.3712	0.7631	0.0736	0.067
H60A	0.2117	0.6418	0.2947	0.036
H60B	0.2859	0.6000	0.3430	0.036
H60C	0.1961	0.5274	0.2455	0.036
H62	0.6721	0.9554	0.6650	0.02
H63	0.5910	1.0650	0.6601	0.022
H65	0.3261	0.8453	0.5567	0.021
H66	0.4060	0.7351	0.5576	0.018
H68A	0.3219	1.0137	0.6796	0.057
H68B	0.3336	1.1183	0.6812	0.057
H68C	0.4280	1.1028	0.7379	0.057
H69A	0.5146	1.1679	0.6584	0.036

	x/a	y/b	z/c	U(eq)
H69B	0.4161	1.1684	0.5915	0.036
H69C	0.4784	1.1075	0.5511	0.036
H70A	0.3140	0.9565	0.4600	0.049
H70B	0.2659	1.0315	0.5063	0.049
H70C	0.2513	0.9310	0.5135	0.049
H72	0.9765	0.9089	0.9459	0.022
H73	1.0580	0.9755	1.1022	0.024
H75	0.9495	0.7061	1.0826	0.025
H76	0.8683	0.6390	0.9270	0.022
H78A	1.1595	1.0255	1.2413	0.05
H78B	1.1410	1.0274	1.3275	0.05
H78C	1.0571	1.0307	1.2485	0.05
H79A	0.9351	0.8875	1.2372	0.056
H79B	1.0218	0.8867	1.3159	0.056
H79C	0.9573	0.7884	1.2214	0.056
H80A	1.1154	0.7770	1.2050	0.053
H80B	1.1794	0.8754	1.2995	0.053
H80C	1.1931	0.8687	1.2102	0.053

8.2 Crystallographic data for compound **11a**

Singlet crystal of compound **11a** was obtained by slow diffusion of acetonitrile to the toluene solution.

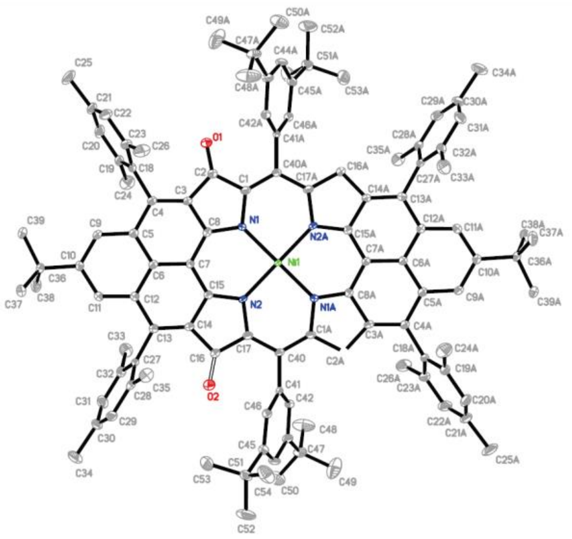

Fig. S63. X-ray crystallographic structure of **11a**. Solvent molecules are omitted for clarity; ellipsoids are set to 50% probability.

Table S11. Crystal data and structure refinement for **11a**

Chemical formula	$C_{136}H_{142}N_4NiO_2$	
Formula weight	1923.24	
Temperature	100(2) K	
Wavelength	0.71073 Å	
Crystal system	triclinic	
Space group	P -1	
Unit cell dimensions	a = 12.8361(14) Å	α = 100.921(4)°
	b = 15.5717(18) Å	β = 109.976(4)°
	c = 15.9281(17) Å	γ = 108.180(4)°
Volume	2682.0(5) $Å^3$	
Z	1	
Density (calculated)	1.191 g/cm^3	
Absorption coefficient	0.237 mm^{-1}	
F(000)	1030	
Theta range for data collection	2.13 to 27.50°	
Index ranges	-16<=h<=16, -20<=k<=20, -20<=l<=20	
Reflections collected	147706	
Independent reflections	12338 [R(int) = 0.0918]	
Structure solution technique	direct methods	
Structure solution program	SHELXS-97 (Sheldrick 2008)	
Refinement method	Full-matrix least-squares on F2	
Refinement program	SHELXL-2013 (Sheldrick, 2013)	
Function minimized	Σ w(Fo2 - Fc2)2	
Data / restraints / parameters	12338 / 1 / 644	
Goodness-of-fit on F2	1.040	
Final R indices	9448 data; I>2σ(I)	R1 = 0.0506, wR2 = 0.1087
	all data	R1 = 0.0789, wR2 = 0.1184
Weighting scheme	$w=1/[\sigma^2(F_o^2)+(0.0424P)^2+2.3179P]$ where $P=(F_o^2+2F_c^2)/3$	
Largest diff. peak and hole	0.504 and -0.544 $eÅ^{-3}$	
R.M.S. deviation from mean	0.058 $eÅ^{-3}$	

Table S12. Atomic coordinates and equivalent isotropic atomic displacement parameters ($Å^2$), U(eq) is defined as one third of the trace of the orthogonalized U_{ij} tensor.

	x/a	y/b	z/c	U(eq)

	x/a	y/b	z/c	U(eq)
C14S	0.867(2)	0.2072(12)	0.0006(10)	0.050(2)
C8S	0.9271(5)	0.2607(3)	0.9497(4)	0.0486(5)
C9S	0.9931(6)	0.2240(2)	0.9118(4)	0.0486(5)
C10S	0.0612(5)	0.2769(3)	0.8731(4)	0.0486(5)
C11S	0.0634(4)	0.3665(3)	0.8725(3)	0.0607(10)
C12S	0.9974(5)	0.4033(2)	0.9104(4)	0.0607(10)
C13S	0.9292(4)	0.3504(3)	0.9490(4)	0.0607(10)
C14X	0.842(2)	0.1816(11)	0.9854(10)	0.050(2)
C8X	0.9058(5)	0.2512(3)	0.9477(4)	0.0486(5)
C9X	0.9950(5)	0.2413(3)	0.9211(4)	0.0486(5)
C10X	0.0476(4)	0.3051(3)	0.8820(4)	0.0486(5)
C11X	0.0110(4)	0.3787(3)	0.8695(3)	0.0607(10)
C12X	0.9218(4)	0.3886(3)	0.8962(4)	0.0607(10)
C13X	0.8692(4)	0.3248(3)	0.9353(4)	0.0607(10)
Ni1	0.0	0.0	0.0	0.01042(9)
N1	0.88167(13)	0.95695(10)	0.05138(10)	0.0122(3)
N2	0.08797(13)	0.12300(10)	0.10293(10)	0.0125(3)
O1	0.6325(3)	0.79524(19)	0.0724(2)	0.0292(7)
O2	0.3340(3)	0.3497(2)	0.2536(2)	0.0300(7)
C1	0.77954(16)	0.86838(12)	0.01645(13)	0.0139(4)
C2	0.71925(17)	0.86385(13)	0.07789(13)	0.0165(4)
C3	0.78499(16)	0.95474(13)	0.15215(13)	0.0154(4)
C4	0.76827(16)	0.99316(13)	0.22920(13)	0.0156(4)
C5	0.85149(16)	0.08866(12)	0.29165(13)	0.0140(4)
C6	0.94900(16)	0.14023(12)	0.27245(12)	0.0127(4)
C7	0.96341(16)	0.09890(12)	0.19153(12)	0.0125(3)
C8	0.88162(16)	0.00693(12)	0.13106(12)	0.0126(3)
C9	0.83956(16)	0.13209(13)	0.37167(13)	0.0148(4)
C10	0.92026(16)	0.22338(13)	0.43405(12)	0.0142(4)
C11	0.01532(16)	0.27319(13)	0.41444(13)	0.0154(4)
C12	0.03217(16)	0.23406(12)	0.33647(12)	0.0141(4)
C13	0.13396(16)	0.28645(13)	0.32044(13)	0.0159(4)
C14	0.14603(16)	0.24361(12)	0.24254(13)	0.0153(4)
C15	0.06075(16)	0.15084(12)	0.17564(12)	0.0128(4)
C16	0.23569(17)	0.27568(13)	0.20825(13)	0.0166(4)
C17	0.19684(16)	0.20091(12)	0.12219(13)	0.0139(4)

	x/a	y/b	z/c	U(eq)
C18	0.66583(17)	0.93726(12)	0.24845(13)	0.0174(4)
C19	0.68948(19)	0.89869(13)	0.32174(14)	0.0214(4)
C20	0.5939(2)	0.85219(14)	0.34237(16)	0.0271(5)
C21	0.4773(2)	0.84311(14)	0.29236(17)	0.0293(5)
C22	0.45560(19)	0.87905(15)	0.21824(17)	0.0287(5)
C23	0.54766(18)	0.92619(14)	0.19463(15)	0.0219(4)
C24	0.8139(2)	0.90364(16)	0.37601(17)	0.0323(5)
C25	0.3765(2)	0.79724(17)	0.3193(2)	0.0446(7)
C26	0.5185(2)	0.96465(17)	0.11346(17)	0.0327(5)
C27	0.22340(16)	0.38426(13)	0.38718(13)	0.0152(4)
C28	0.22204(16)	0.46463(13)	0.36018(13)	0.0177(4)
C29	0.30261(18)	0.55517(14)	0.42550(15)	0.0233(4)
C30	0.38230(18)	0.56794(14)	0.51593(15)	0.0256(5)
C31	0.38349(18)	0.48728(15)	0.54013(14)	0.0253(5)
C32	0.30571(17)	0.39534(14)	0.47729(13)	0.0196(4)
C33	0.3120(2)	0.31029(15)	0.50785(15)	0.0284(5)
C34	0.4643(2)	0.66695(16)	0.58636(19)	0.0430(7)
C35	0.13469(19)	0.45534(16)	0.26435(15)	0.0280(5)
C36	0.91291(17)	0.27054(13)	0.52439(13)	0.0165(4)
C37	0.91277(18)	0.36946(14)	0.52670(14)	0.0215(4)
C38	0.02426(19)	0.28187(15)	0.60989(14)	0.0250(4)
C39	0.79823(19)	0.21087(14)	0.53093(15)	0.0251(5)
C40	0.25878(15)	0.20497(12)	0.06580(12)	0.0126(3)
C41	0.37476(16)	0.29134(12)	0.09919(12)	0.0128(4)
C42	0.48409(16)	0.28160(13)	0.13417(13)	0.0152(4)
C43	0.59414(16)	0.36061(13)	0.17233(13)	0.0169(4)
C44	0.59101(17)	0.45002(13)	0.17613(13)	0.0184(4)
C45	0.48354(17)	0.46220(13)	0.14191(13)	0.0170(4)
C46	0.37481(16)	0.38031(12)	0.10142(12)	0.0148(4)
C47	0.71559(17)	0.35077(14)	0.20623(15)	0.0220(4)
C48	0.7033(2)	0.25546(17)	0.2217(2)	0.0468(7)
C49	0.7614(2)	0.3571(2)	0.12955(18)	0.0483(7)
C50	0.8094(2)	0.43066(17)	0.29825(17)	0.0348(5)
C51	0.48054(18)	0.56114(13)	0.15108(14)	0.0203(4)
C52	0.6074(2)	0.64186(15)	0.1932(2)	0.0406(6)
C53	0.4189(2)	0.57938(16)	0.21573(17)	0.0344(5)

	**x/a**	**y/b**	**z/c**	**U(eq)**
C54	0.4075(2)	0.56435(17)	0.05456(16)	0.0386(6)
C1S	0.2292(3)	0.01247(18)	0.3383(2)	0.0414(6)
C2S	0.3394(3)	0.03335(17)	0.41146(18)	0.0424(6)
C3S	0.4463(3)	0.06667(17)	0.4018(2)	0.0477(7)
C4S	0.4452(3)	0.08152(17)	0.3192(2)	0.0473(7)
C5S	0.3363(3)	0.06120(19)	0.2457(2)	0.0462(7)
C6S	0.2295(3)	0.0263(2)	0.2551(2)	0.0470(7)
C7S	0.1124(3)	0.9764(3)	0.3479(3)	0.0657(9)

Table S13. Band length **(Å)** and Bond angles (°) for **11a**

C14S-C8S	1.495(5)	C14S-H14A	0.98
C14S-H14B	0.98	C14S-H14C	0.98
C8S-C9S	1.39	C8S-C13S	1.39
C9S-C10S	1.39	C9S-H9S	0.95
C10S-C11S	1.39	C10S-H10S	0.95
C11S-C12S	1.39	C11S-H11S	0.95
C12S-C13S	1.39	C12S-H12S	0.95
C13S-H13S	0.95	C14X-C8X	1.494(5)
C14X-H14X	0.98	C14X-H14Y	0.98
C14X-H14Z	0.98	C8X-C9X	1.39
C8X-C13X	1.39	C9X-C10X	1.39
C9X-H9X	0.95	C10X-C11X	1.39
C10X-H10X	0.95	C11X-C12X	1.39
C11X-H11X	0.95	C12X-C13X	1.39
C12X-H12X	0.95	C13X-H13X	0.95
Ni1-N2	1.9563(14)	Ni1-N2	1.9563(14)
Ni1-N1	1.9615(14)	Ni1-N1	1.9616(14)
N1-C8	1.358(2)	N1-C1	1.419(2)
N2-C15	1.353(2)	N2-C17	1.424(2)
O1-C2	1.243(3)	O2-C16	1.264(3)
C1-C40	1.380(2)	C1-C2	1.439(2)
C2-C3	1.433(2)	C2-H2	0.95
C3-C4	1.375(2)	C3-C8	1.439(2)
C4-C5	1.437(2)	C4-C18	1.498(2)
C5-C9	1.406(2)	C5-C6	1.426(2)

C6-C12	1.426(2)	C6-C7	1.426(2)
C7-C8	1.389(2)	C7-C15	1.394(2)
C9-C10	1.387(2)	C9-H9	0.95
C10-C11	1.396(2)	C10-C36	1.534(2)
C11-C12	1.393(2)	C11-H11	0.95
C12-C13	1.442(2)	C13-C14	1.375(2)
C13-C27	1.493(2)	C14-C16	1.431(2)
C14-C15	1.433(2)	C16-C17	1.432(2)
C16-H16	0.95	C17-C40	1.386(2)
C18-C19	1.398(3)	C18-C23	1.399(3)
C19-C20	1.396(3)	C19-C24	1.503(3)
C20-C21	1.381(3)	C20-H20	0.95
C21-C22	1.381(3)	C21-C25	1.511(3)
C22-C23	1.394(3)	C22-H22	0.95
C23-C26	1.508(3)	C24-H24A	0.98
C24-H24B	0.98	C24-H24C	0.98
C25-H25A	0.98	C25-H25B	0.98
C25-H25C	0.98	C26-H26A	0.98
C26-H26B	0.98	C26-H26C	0.98
C27-C32	1.401(3)	C27-C28	1.401(3)
C28-C29	1.392(3)	C28-C35	1.501(3)
C29-C30	1.385(3)	C29-H29	0.95
C30-C31	1.385(3)	C30-C34	1.506(3)
C31-C32	1.389(3)	C31-H31	0.95
C32-C33	1.510(3)	C33-H33A	0.98
C33-H33B	0.98	C33-H33C	0.98
C34-H34A	0.98	C34-H34B	0.98
C34-H34C	0.98	C35-H35A	0.98
C35-H35B	0.98	C35-H35C	0.98
C36-C39	1.526(2)	C36-C38	1.533(3)
C36-C37	1.534(3)	C37-H37A	0.98
C37-H37B	0.98	C37-H37C	0.98
C38-H38A	0.98	C38-H38B	0.98
C38-H38C	0.98	C39-H39A	0.98
C39-H39B	0.98	C39-H39C	0.98
C40-C1	1.380(2)	C40-C41	1.499(2)
C41-C46	1.379(2)	C41-C42	1.390(3)

C42-C43	1.387(2)	C42-H42	0.95
C43-C44	1.396(3)	C43-C47	1.533(3)
C44-C45	1.387(3)	C44-H44	0.95
C45-C46	1.397(2)	C45-C51	1.534(3)
C46-H46	0.95	C47-C48	1.519(3)
C47-C50	1.521(3)	C47-C49	1.532(3)
C48-H48A	0.98	C48-H48B	0.98
C48-H48C	0.98	C49-H49A	0.98
C49-H49B	0.98	C49-H49C	0.98
C50-H50A	0.98	C50-H50B	0.98
C50-H50C	0.98	C51-C54	1.523(3)
C51-C52	1.530(3)	C51-C53	1.534(3)
C52-H52A	0.98	C52-H52B	0.98
C52-H52C	0.98	C53-H53A	0.98
C53-H53B	0.98	C53-H53C	0.98
C54-H54A	0.98	C54-H54B	0.98
C54-H54C	0.98	C1S-C2S	1.382(4)
C1S-C6S	1.383(4)	C1S-C7S	1.501(4)
C2S-C3S	1.380(4)	C2S-H2S	0.95
C3S-C4S	1.375(4)	C3S-H3S	0.95
C4S-C5S	1.375(4)	C4S-H4S	0.95
C5S-C6S	1.379(4)	C5S-H5S	0.95
C6S-H6S	0.95	C7S-H7S1	0.98
C7S-H7S2	0.98	C7S-H7S3	0.98

C8S-C14S-H14A	109.5	C8S-C14S-H14B	109.5
H14A-C14S-H14B	109.5	C8S-C14S-H14C	109.5
H14A-C14S-H14C	109.5	H14B-C14S-H14C	109.5
C9S-C8S-C13S	120.0	C9S-C8S-C14S	118.4(11)
C13S-C8S-C14S	121.2(11)	C8S-C9S-C10S	120.0
C8S-C9S-H9S	120.0	C10S-C9S-H9S	120.0
C11S-C10S-C9S	120.0	C11S-C10S-H10S	120.0
C9S-C10S-H10S	120.0	C10S-C11S-C12S	120.0
C10S-C11S-H11S	120.0	C12S-C11S-H11S	120.0
C11S-C12S-C13S	120.0	C11S-C12S-H12S	120.0
C13S-C12S-H12S	120.0	C12S-C13S-C8S	120.0
C12S-C13S-H13S	120.0	C8S-C13S-H13S	120.0

C8X-C14X-H14X	109.5	C8X-C14X-H14Y	109.5
H14X-C14X-H14Y	109.5	C8X-C14X-H14Z	109.5
H14X-C14X-H14Z	109.5	H14Y-C14X-H14Z	109.5
C9X-C8X-C13X	120.0	C9X-C8X-C14X	122.0(12)
C13X-C8X-C14X	118.0(12)	C10X-C9X-C8X	120.0
C10X-C9X-H9X	120.0	C8X-C9X-H9X	120.0
C9X-C10X-C11X	120.0	C9X-C10X-H10X	120.0
C11X-C10X-H10X	120.0	C12X-C11X-C10X	120.0
C12X-C11X-H11X	120.0	C10X-C11X-H11X	120.0
C13X-C12X-C11X	120.0	C13X-C12X-H12X	120.0
C11X-C12X-H12X	120.0	C12X-C13X-C8X	120.0
C12X-C13X-H13X	120.0	C8X-C13X-H13X	120.0
N2-Ni1-N2	180.0	N2-Ni1-N1	89.00(6)
N2-Ni1-N1	91.00(6)	N2-Ni1-N1	91.00(6)
N2-Ni1-N1	89.00(6)	N1-Ni1-N1	180.0
C8-N1-C1	104.38(13)	C8-N1-Ni1	126.55(11)
C1-N1-Ni1	129.06(11)	C15-N2-C17	104.16(14)
C15-N2-Ni1	126.76(11)	C17-N2-Ni1	129.00(11)
C40-C1-N1	125.14(15)	C40-C1-C2	123.46(16)
N1-C1-C2	111.40(15)	O1-C2-C3	125.9(2)
O1-C2-C1	128.6(2)	C3-C2-C1	105.45(15)
C3-C2-H2	127.3	C1-C2-H2	127.3
C4-C3-C2	132.22(16)	C4-C3-C8	122.55(16)
C2-C3-C8	105.22(15)	C3-C4-C5	118.66(16)
C3-C4-C18	121.17(16)	C5-C4-C18	120.17(15)
C9-C5-C6	119.26(16)	C9-C5-C4	121.52(16)
C6-C5-C4	119.21(15)	C5-C6-C12	118.34(15)
C5-C6-C7	120.88(16)	C12-C6-C7	120.78(15)
C8-C7-C15	121.24(16)	C8-C7-C6	119.39(15)
C15-C7-C6	119.35(16)	N1-C8-C7	127.20(16)
N1-C8-C3	113.52(15)	C7-C8-C3	119.28(15)
C10-C9-C5	122.54(16)	C10-C9-H9	118.7
C5-C9-H9	118.7	C9-C10-C11	117.65(16)
C9-C10-C36	123.59(15)	C11-C10-C36	118.73(15)
C12-C11-C10	122.55(16)	C12-C11-H11	118.7
C10-C11-H11	118.7	C11-C12-C6	119.64(16)
C11-C12-C13	120.91(16)	C6-C12-C13	119.44(15)

C14-C13-C12	118.11(16)	C14-C13-C27	121.38(15)
C12-C13-C27	120.51(15)	C13-C14-C16	131.50(17)
C13-C14-C15	123.11(16)	C16-C14-C15	105.38(15)
N2-C15-C7	127.23(16)	N2-C15-C14	113.61(15)
C7-C15-C14	119.14(15)	O2-C16-C14	124.6(2)
O2-C16-C17	129.5(2)	C14-C16-C17	105.41(15)
C14-C16-H16	127.3	C17-C16-H16	127.3
C40-C17-N2	125.00(16)	C40-C17-C16	123.58(16)
N2-C17-C16	111.41(15)	C19-C18-C23	120.46(17)
C19-C18-C4	119.72(18)	C23-C18-C4	119.80(17)
C20-C19-C18	118.7(2)	C20-C19-C24	119.85(19)
C18-C19-C24	121.39(17)	C21-C20-C19	121.9(2)
C21-C20-H20	119.0	C19-C20-H20	119.0
C20-C21-C22	118.15(18)	C20-C21-C25	121.1(2)
C22-C21-C25	120.8(2)	C21-C22-C23	122.3(2)
C21-C22-H22	118.9	C23-C22-H22	118.9
C22-C23-C18	118.39(19)	C22-C23-C26	119.79(19)
C18-C23-C26	121.82(17)	C19-C24-H24A	109.5
C19-C24-H24B	109.5	H24A-C24-H24B	109.5
C19-C24-H24C	109.5	H24A-C24-H24C	109.5
H24B-C24-H24C	109.5	C21-C25-H25A	109.5
C21-C25-H25B	109.5	H25A-C25-H25B	109.5
C21-C25-H25C	109.5	H25A-C25-H25C	109.5
H25B-C25-H25C	109.5	C23-C26-H26A	109.5
C23-C26-H26B	109.5	H26A-C26-H26B	109.5
C23-C26-H26C	109.5	H26A-C26-H26C	109.5
H26B-C26-H26C	109.5	C32-C27-C28	120.42(17)
C32-C27-C13	119.88(17)	C28-C27-C13	119.68(17)
C29-C28-C27	118.43(18)	C29-C28-C35	119.77(18)
C27-C28-C35	121.79(17)	C30-C29-C28	122.15(19)
C30-C29-H29	118.9	C28-C29-H29	118.9
C31-C30-C29	118.22(18)	C31-C30-C34	121.0(2)
C29-C30-C34	120.8(2)	C30-C31-C32	121.9(2)
C30-C31-H31	119.1	C32-C31-H31	119.1
C31-C32-C27	118.87(18)	C31-C32-C33	119.27(18)
C27-C32-C33	121.85(17)	C32-C33-H33A	109.5
C32-C33-H33B	109.5	H33A-C33-H33B	109.5

C32-C33-H33C	109.5	H33A-C33-H33C	109.5
H33B-C33-H33C	109.5	C30-C34-H34A	109.5
C30-C34-H34B	109.5	H34A-C34-H34B	109.5
C30-C34-H34C	109.5	H34A-C34-H34C	109.5
H34B-C34-H34C	109.5	C28-C35-H35A	109.5
C28-C35-H35B	109.5	H35A-C35-H35B	109.5
C28-C35-H35C	109.5	H35A-C35-H35C	109.5
H35B-C35-H35C	109.5	C39-C36-C38	108.97(16)
C39-C36-C10	112.13(15)	C38-C36-C10	108.23(15)
C39-C36-C37	107.98(16)	C38-C36-C37	109.45(16)
C10-C36-C37	110.04(15)	C36-C37-H37A	109.5
C36-C37-H37B	109.5	H37A-C37-H37B	109.5
C36-C37-H37C	109.5	H37A-C37-H37C	109.5
H37B-C37-H37C	109.5	C36-C38-H38A	109.5
C36-C38-H38B	109.5	H38A-C38-H38B	109.5
C36-C38-H38C	109.5	H38A-C38-H38C	109.5
H38B-C38-H38C	109.5	C36-C39-H39A	109.5
C36-C39-H39B	109.5	H39A-C39-H39B	109.5
C36-C39-H39C	109.5	H39A-C39-H39C	109.5
H39B-C39-H39C	109.5	C1-C40-C17	122.67(16)
C1-C40-C41	119.66(15)	C17-C40-C41	117.61(15)
C46-C41-C42	119.98(16)	C46-C41-C40	121.26(16)
C42-C41-C40	118.62(16)	C43-C42-C41	120.88(17)
C43-C42-H42	119.6	C41-C42-H42	119.6
C42-C43-C44	117.68(17)	C42-C43-C47	121.73(17)
C44-C43-C47	120.55(16)	C45-C44-C43	122.86(17)
C45-C44-H44	118.6	C43-C44-H44	118.6
C44-C45-C46	117.54(17)	C44-C45-C51	122.61(16)
C46-C45-C51	119.79(17)	C41-C46-C45	120.99(17)
C41-C46-H46	119.5	C45-C46-H46	119.5
C48-C47-C50	108.28(19)	C48-C47-C49	108.2(2)
C50-C47-C49	108.92(19)	C48-C47-C43	112.38(16)
C50-C47-C43	111.19(17)	C49-C47-C43	107.81(17)
C47-C48-H48A	109.5	C47-C48-H48B	109.5
H48A-C48-H48B	109.5	C47-C48-H48C	109.5
H48A-C48-H48C	109.5	H48B-C48-H48C	109.5
C47-C49-H49A	109.5	C47-C49-H49B	109.5

H49A-C49-H49B	109.5	C47-C49-H49C	109.5
H49A-C49-H49C	109.5	H49B-C49-H49C	109.5
C47-C50-H50A	109.5	C47-C50-H50B	109.5
H50A-C50-H50B	109.5	C47-C50-H50C	109.5
H50A-C50-H50C	109.5	H50B-C50-H50C	109.5
C54-C51-C52	108.10(19)	C54-C51-C45	110.38(16)
C52-C51-C45	112.58(17)	C54-C51-C53	108.61(19)
C52-C51-C53	108.57(18)	C45-C51-C53	108.51(16)
C51-C52-H52A	109.5	C51-C52-H52B	109.5
H52A-C52-H52B	109.5	C51-C52-H52C	109.5
H52A-C52-H52C	109.5	H52B-C52-H52C	109.5
C51-C53-H53A	109.5	C51-C53-H53B	109.5
H53A-C53-H53B	109.5	C51-C53-H53C	109.5
H53A-C53-H53C	109.5	H53B-C53-H53C	109.5
C51-C54-H54A	109.5	C51-C54-H54B	109.5
H54A-C54-H54B	109.5	C51-C54-H54C	109.5
H54A-C54-H54C	109.5	H54B-C54-H54C	109.5
C2S-C1S-C6S	117.8(3)	C2S-C1S-C7S	121.6(2)
C6S-C1S-C7S	120.5(3)	C3S-C2S-C1S	121.1(2)
C3S-C2S-H2S	119.4	C1S-C2S-H2S	119.4
C4S-C3S-C2S	120.4(3)	C4S-C3S-H3S	119.8
C2S-C3S-H3S	119.8	C5S-C4S-C3S	119.2(3)
C5S-C4S-H4S	120.4	C3S-C4S-H4S	120.4
C4S-C5S-C6S	120.2(3)	C4S-C5S-H5S	119.9
C6S-C5S-H5S	119.9	C5S-C6S-C1S	121.3(3)
C5S-C6S-H6S	119.3	C1S-C6S-H6S	119.3
C1S-C7S-H7S1	109.5	C1S-C7S-H7S2	109.5
H7S1-C7S-H7S2	109.5	C1S-C7S-H7S3	109.5
H7S1-C7S-H7S3	109.5	H7S2-C7S-H7S3	109.5

Table S14. Anisotropic atomic displacement parameters ($Å^2$) for **11a**, The anisotropic atomic isplacement factor exponent takes the form: $-2\pi^2[h^2 a^{*2} U_{11} + ... + 2 h k a^* b^* U_{12}]$

	**U_{11}**	**U_{22}**	**U_{33}**	**U_{23}**	**U_{13}**	**U_{12}**
C14S	0.039(7)	0.044(8)	0.051(4)	0.009(5)	0.005(5)	0.016(5)
C8S	0.0518(12)	0.0401(12)	0.0415(11)	0.0146(10)	0.0057(9)	0.0188(10)
C9S	0.0518(12)	0.0401(12)	0.0415(11)	0.0146(10)	0.0057(9)	0.0188(10)
C10S	0.0518(12)	0.0401(12)	0.0415(11)	0.0146(10)	0.0057(9)	0.0188(10)

	U_{11}	U_{22}	U_{33}	U_{23}	U_{13}	U_{12}
C11S	0.095(3)	0.0405(14)	0.0439(15)	0.0141(11)	0.026(2)	0.0278(17)
C12S	0.095(3)	0.0405(14)	0.0439(15)	0.0141(11)	0.026(2)	0.0278(17)
C13S	0.095(3)	0.0405(14)	0.0439(15)	0.0141(11)	0.026(2)	0.0278(17)
C14X	0.039(7)	0.044(8)	0.051(4)	0.009(5)	0.005(5)	0.016(5)
C8X	0.0518(12)	0.0401(12)	0.0415(11)	0.0146(10)	0.0057(9)	0.0188(10)
C9X	0.0518(12)	0.0401(12)	0.0415(11)	0.0146(10)	0.0057(9)	0.0188(10)
C10X	0.0518(12)	0.0401(12)	0.0415(11)	0.0146(10)	0.0057(9)	0.0188(10)
C11X	0.095(3)	0.0405(14)	0.0439(15)	0.0141(11)	0.026(2)	0.0278(17)
C12X	0.095(3)	0.0405(14)	0.0439(15)	0.0141(11)	0.026(2)	0.0278(17)
C13X	0.095(3)	0.0405(14)	0.0439(15)	0.0141(11)	0.026(2)	0.0278(17)
Ni1	0.00897(16)	0.00958(16)	0.01021(16)	0.00069(12)	0.00613(13)	0.00002(12)
N1	0.0106(7)	0.0101(7)	0.0123(7)	0.0004(6)	0.0060(6)	0.0006(6)
N2	0.0090(7)	0.0135(7)	0.0133(7)	0.0028(6)	0.0072(6)	0.0006(6)
O1	0.0307(16)	0.0178(14)	0.0314(16)	-0.0001(12)	0.0221(14)	-0.0047(12)
O2	0.0233(15)	0.0280(16)	0.0254(16)	-0.0021(13)	0.0136(13)	-0.0032(13)
C1	0.0117(9)	0.0114(8)	0.0173(9)	0.0043(7)	0.0072(7)	0.0025(7)
C2	0.0168(9)	0.0130(9)	0.0185(9)	0.0032(7)	0.0100(8)	0.0031(7)
C3	0.0135(9)	0.0129(9)	0.0181(9)	0.0034(7)	0.0094(8)	0.0013(7)
C4	0.0162(9)	0.0126(9)	0.0186(9)	0.0040(7)	0.0110(8)	0.0034(7)
C5	0.0140(9)	0.0135(9)	0.0152(9)	0.0050(7)	0.0080(7)	0.0046(7)
C6	0.0125(9)	0.0127(8)	0.0136(9)	0.0045(7)	0.0070(7)	0.0044(7)
C7	0.0134(9)	0.0118(8)	0.0120(8)	0.0034(7)	0.0070(7)	0.0032(7)
C8	0.0120(8)	0.0137(8)	0.0133(9)	0.0044(7)	0.0070(7)	0.0051(7)
C9	0.0141(9)	0.0151(9)	0.0169(9)	0.0057(7)	0.0099(8)	0.0043(7)
C10	0.0158(9)	0.0144(9)	0.0134(9)	0.0036(7)	0.0082(7)	0.0057(7)
C11	0.0156(9)	0.0128(9)	0.0145(9)	0.0011(7)	0.0068(8)	0.0032(7)
C12	0.0138(9)	0.0137(9)	0.0137(9)	0.0029(7)	0.0073(7)	0.0036(7)
C13	0.0145(9)	0.0138(9)	0.0151(9)	0.0019(7)	0.0072(8)	0.0012(7)
C14	0.0129(9)	0.0129(9)	0.0164(9)	0.0020(7)	0.0080(8)	0.0009(7)
C15	0.0120(9)	0.0127(8)	0.0121(8)	0.0030(7)	0.0056(7)	0.0032(7)
C16	0.0150(9)	0.0156(9)	0.0179(9)	0.0033(7)	0.0102(8)	0.0025(8)
C17	0.0111(9)	0.0126(8)	0.0168(9)	0.0049(7)	0.0066(7)	0.0026(7)
C18	0.0205(10)	0.0104(8)	0.0205(10)	-0.0001(7)	0.0152(8)	0.0013(7)
C19	0.0296(11)	0.0118(9)	0.0259(10)	0.0034(8)	0.0198(9)	0.0053(8)
C20	0.0425(13)	0.0162(10)	0.0351(12)	0.0100(9)	0.0312(11)	0.0106(9)
C21	0.0363(13)	0.0136(10)	0.0465(14)	0.0053(9)	0.0347(12)	0.0046(9)

	U_{11}	U_{22}	U_{33}	U_{23}	U_{13}	U_{12}
C22	0.0207(11)	0.0206(10)	0.0439(13)	0.0047(9)	0.0200(10)	0.0039(9)
C23	0.0211(10)	0.0157(9)	0.0279(11)	0.0039(8)	0.0150(9)	0.0030(8)
C24	0.0365(13)	0.0313(12)	0.0373(13)	0.0199(10)	0.0194(11)	0.0151(10)
C25	0.0508(16)	0.0271(12)	0.0753(19)	0.0159(12)	0.0551(16)	0.0095(11)
C26	0.0210(11)	0.0348(13)	0.0369(13)	0.0128(10)	0.0107(10)	0.0059(10)
C27	0.0124(9)	0.0142(9)	0.0169(9)	0.0003(7)	0.0102(8)	0.0014(7)
C28	0.0138(9)	0.0201(9)	0.0214(10)	0.0048(8)	0.0123(8)	0.0058(8)
C29	0.0201(10)	0.0159(9)	0.0381(12)	0.0056(9)	0.0196(9)	0.0065(8)
C30	0.0154(10)	0.0177(10)	0.0328(12)	-0.0065(9)	0.0115(9)	0.0006(8)
C31	0.0179(10)	0.0272(11)	0.0196(10)	-0.0034(8)	0.0045(8)	0.0058(9)
C32	0.0179(10)	0.0209(10)	0.0183(10)	0.0020(8)	0.0098(8)	0.0062(8)
C33	0.0297(12)	0.0293(11)	0.0236(11)	0.0077(9)	0.0084(9)	0.0127(10)
C34	0.0308(13)	0.0231(12)	0.0493(16)	-0.0140(11)	0.0160(12)	-0.0042(10)
C35	0.0260(11)	0.0310(12)	0.0297(12)	0.0125(9)	0.0136(10)	0.0118(9)
C36	0.0174(9)	0.0162(9)	0.0158(9)	0.0023(7)	0.0113(8)	0.0042(8)
C37	0.0222(10)	0.0191(10)	0.0223(10)	0.0012(8)	0.0124(9)	0.0078(8)
C38	0.0292(11)	0.0316(11)	0.0160(10)	0.0060(8)	0.0121(9)	0.0133(9)
C39	0.0266(11)	0.0229(10)	0.0251(11)	0.0010(8)	0.0196(9)	0.0038(9)
C40	0.0098(8)	0.0117(8)	0.0151(9)	0.0039(7)	0.0061(7)	0.0023(7)
C41	0.0124(9)	0.0126(8)	0.0103(8)	0.0005(7)	0.0071(7)	0.0007(7)
C42	0.0165(9)	0.0114(8)	0.0167(9)	0.0035(7)	0.0091(8)	0.0030(7)
C43	0.0127(9)	0.0168(9)	0.0179(9)	0.0052(7)	0.0065(8)	0.0022(7)
C44	0.0136(9)	0.0130(9)	0.0194(10)	0.0027(7)	0.0049(8)	-0.0019(7)
C45	0.0197(10)	0.0125(9)	0.0161(9)	0.0029(7)	0.0082(8)	0.0037(7)
C46	0.0124(9)	0.0154(9)	0.0147(9)	0.0027(7)	0.0063(7)	0.0039(7)
C47	0.0123(9)	0.0226(10)	0.0265(11)	0.0080(8)	0.0052(8)	0.0050(8)
C48	0.0179(12)	0.0307(13)	0.079(2)	0.0203(13)	0.0054(12)	0.0105(10)
C49	0.0264(13)	0.092(2)	0.0394(15)	0.0248(15)	0.0177(12)	0.0347(14)
C50	0.0212(11)	0.0333(13)	0.0331(13)	0.0051(10)	-0.0016(10)	0.0087(10)
C51	0.0243(10)	0.0123(9)	0.0221(10)	0.0053(8)	0.0094(9)	0.0057(8)
C52	0.0339(13)	0.0129(10)	0.0650(18)	0.0079(11)	0.0192(13)	0.0025(10)
C53	0.0482(15)	0.0198(11)	0.0399(13)	0.0063(10)	0.0268(12)	0.0129(10)
C54	0.0589(17)	0.0250(12)	0.0299(12)	0.0104(10)	0.0129(12)	0.0209(12)
C1S	0.0541(16)	0.0394(14)	0.0499(16)	0.0262(12)	0.0271(14)	0.0309(13)
C2S	0.0614(18)	0.0251(12)	0.0364(14)	0.0116(10)	0.0192(13)	0.0135(12)
C3S	0.0450(16)	0.0267(13)	0.0436(15)	0.0037(11)	0.0065(13)	-0.0009(12)

	U_{11}	U_{22}	U_{33}	U_{23}	U_{13}	U_{12}
C4S	0.0556(18)	0.0241(12)	0.0599(18)	0.0077(12)	0.0311(15)	0.0100(12)
C5S	0.075(2)	0.0418(15)	0.0503(17)	0.0249(13)	0.0392(16)	0.0386(15)
C6S	0.0599(18)	0.0563(17)	0.0511(16)	0.0320(14)	0.0277(15)	0.0434(15)
C7S	0.065(2)	0.093(3)	0.080(2)	0.058(2)	0.0444(19)	0.050(2)

Table S15. Hydrogen atomic coordinates and isotropic atomic displacement parameters (Å^2) for **11a**

	x/a	y/b	z/c	U(eq)
H14A	0.8197	0.2390	1.0200	0.075
H14B	0.9287	0.2062	1.0568	0.075
H14C	0.8124	0.1414	0.9585	0.075
H9S	0.9916	0.1627	0.9122	0.058
H10S	1.1063	0.2517	0.8472	0.058
H11S	1.1099	0.4027	0.8461	0.073
H12S	0.9988	0.4646	0.9099	0.073
H13S	0.8841	0.3755	0.9750	0.073
H14X	0.7918	0.2058	1.0086	0.075
H14Y	0.9023	0.1737	1.0375	0.075
H14Z	0.7906	0.1197	0.9349	0.075
H9X	1.0200	0.1910	0.9296	0.058
H10X	1.1086	0.2983	0.8637	0.058
H11X	1.0470	0.4222	0.8428	0.073
H12X	0.8968	0.4389	0.8877	0.073
H13X	0.8083	0.3316	0.9535	0.073
H2	-0.3496	-0.1884	0.0707	0.02
H9	-0.2263	0.0976	0.3835	0.018
H11	0.0707	0.3362	0.4559	0.019
H16	0.3061	0.3342	0.2366	0.02
H20	-0.3904	-0.1740	0.3923	0.032
H22	-0.6248	-0.1286	0.1821	0.034
H24A	-0.1593	-0.1255	0.3319	0.048
H24B	-0.1887	-0.1311	0.4211	0.048
H24C	-0.1290	-0.0292	0.4105	0.048
H25A	-0.5950	-0.2322	0.3666	0.067
H25B	-0.6937	-0.2520	0.2629	0.067
H25C	-0.6471	-0.1538	0.3457	0.067
H26A	-0.4852	-0.0784	0.0579	0.049

	x/a	y/b	z/c	U(eq)
H26B	-0.4183	0.0285	0.1314	0.049
H26C	-0.5602	-0.0312	0.0982	0.049
H29	0.3029	0.6100	0.4075	0.028
H31	0.4390	0.4950	0.6014	0.03
H33A	0.2550	0.2918	0.5360	0.043
H33B	0.3946	0.3274	0.5547	0.043
H33C	0.2904	0.2565	0.4527	0.043
H34A	0.4436	0.6758	0.6403	0.065
H34B	0.4537	0.7147	0.5560	0.065
H34C	0.5490	0.6747	0.6086	0.065
H35A	0.1356	0.5184	0.2637	0.042
H35B	0.0527	0.4114	0.2506	0.042
H35C	0.1587	0.4301	0.2162	0.042
H37A	-0.1605	0.3620	0.4738	0.032
H37B	-0.0152	0.4087	0.5211	0.032
H37C	-0.0861	0.4009	0.5866	0.032
H38A	0.0211	0.3117	0.6684	0.037
H38B	0.0983	0.3224	0.6076	0.037
H38C	0.0246	0.2187	0.6083	0.037
H39A	-0.2734	0.2028	0.4762	0.038
H39B	-0.2031	0.2436	0.5893	0.038
H39C	-0.2024	0.1479	0.5316	0.038
H42	0.4834	0.2200	0.1319	0.018
H44	0.6657	0.5049	0.2033	0.022
H46	0.2998	0.3860	0.0750	0.018
H48A	0.7838	0.2542	0.2474	0.07
H48B	0.6512	0.2033	0.1612	0.07
H48C	0.6672	0.2474	0.2663	0.07
H49A	0.8391	0.3509	0.1494	0.072
H49B	0.7726	0.4192	0.1200	0.072
H49C	0.7019	0.3055	0.0702	0.072
H50A	0.7767	0.4320	0.3456	0.052
H50B	0.8286	0.4922	0.2870	0.052
H50C	0.8833	0.4192	0.3215	0.052
H52A	0.6551	0.6427	0.2566	0.061
H52B	0.6007	0.7034	0.1976	0.061

	x/a	y/b	z/c	U(eq)
H52C	0.6479	0.6312	0.1525	0.061
H53A	0.3360	0.5298	0.1880	0.052
H53B	0.4162	0.6424	0.2222	0.052
H53C	0.4651	0.5776	0.2782	0.052
H54A	0.4463	0.5532	0.0126	0.058
H54B	0.4045	0.6273	0.0617	0.058
H54C	0.3248	0.5146	0.0272	0.058
H2S	0.3417	0.0246	0.4694	0.051
H3S	0.5210	0.0794	0.4526	0.057
H4S	0.5190	0.1055	0.3129	0.057
H5S	0.3346	0.0713	0.1883	0.055
H6S	0.1547	0.0114	0.2034	0.056
H7S1	0.1294	-0.0272	0.4117	0.099
H7S2	0.0589	-0.0875	0.3010	0.099
H7S3	0.0725	0.0203	0.3373	0.099

8.3 Crystallographic data for compound **11b**

Singlet crystal of compound **11b** were obtained by slow diffusion of acetonitrile to the toluene solution.

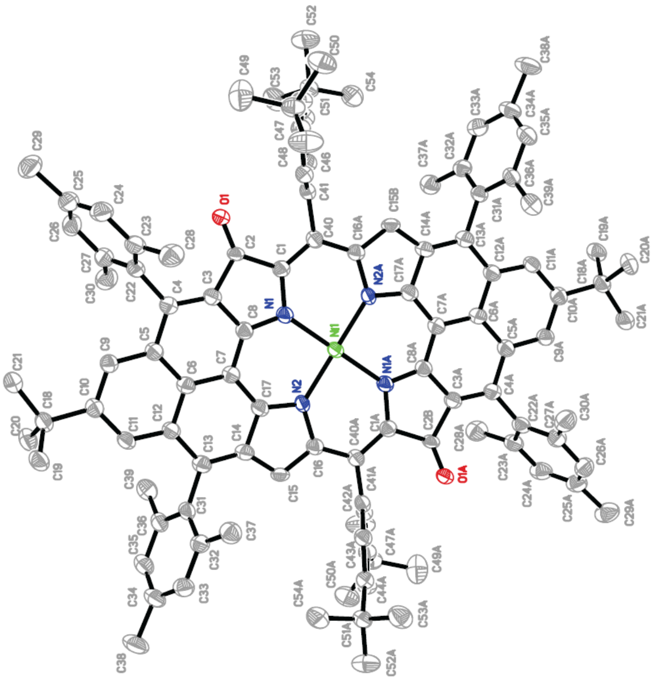

Fig. S64. X-ray crystallographic structure of **11b**. Solvent molecules are omitted for clarity; ellipsoids are set to 50% probability.

Table S16. Crystal data and structure refinement for **11b**

Chemical formula	$C_{136}H_{142}N_4NiO_2$	
Formula weight	1923.24	
Temperature	100(2) K	
Wavelength	1.54178 Å	
Crystal size	0.030 x 0.110 x 0.150 mm	
Crystal system	triclinic	
Space group	P -1	
Unit cell dimensions	a = 12.8500(12) Å	α = 100.951(5)°
	b = 15.5881(15) Å	β = 110.033(5)°
	c = 15.9283(15) Å	γ = 108.118(5)°
Volume	2687.2(5) $Å^3$	
Z	1	
Density (calculated)	1.188 g/cm^3	
Absorption coefficient	0.684 mm^{-1}	
F(000)	1030	
Theta range for data collection	3.13 to 66.58°	
Index ranges	-15<=h<=15, -18<=k<=16, -18<=l<=18	
Reflections collected	36244	
Independent reflections	9117 [R(int) = 0.0839]	
Max. and min. transmission	0.7528 and 0.4989	
Structure solution technique	direct methods	
Structure solution program	SHELXS-97 (Sheldrick 2008)	
Refinement method	Full-matrix least-squares on F2	
Refinement program	SHELXL-2013 (Sheldrick, 2013)	
Function minimized	Σ w(Fo2 - Fc2)2	
Data / restraints / parameters	9117 / 48 / 682	
Goodness-of-fit on F2	1.048	
Δ/σmax	0.001	
Final R indices	6330 data; I>2σ(I)	R1 = 0.0771, wR2 = 0.2016
	all data	R1 = 0.1098, wR2 = 0.2360
Weighting scheme	$w=1/[\sigma^2(F_o^2)+(0.1538P)^2+0.2272P]$ where $P=(F_o^2+2F_c^2)/3$	
Largest diff. peak and hole	0.985 and -0.635 e$Å^{-3}$	
R.M.S. deviation from mean	0.083 e$Å^{-3}$	

Table S17. Atomic coordinates and equivalent isotropic atomic displacement parameters ($Å^2$) for **11b**,

U(eq) is defined as one third of the trace of the orthogonalized U_{ij} tensor.

	**x/a**	**y/b**	**z/c**	**U(eq)**
Ni1	0.0	0.0	0.0	0.0284(2)
O1	0.6323(3)	0.7940(2)	0.0724(2)	0.0387(11)
O2	0.3352(7)	0.3502(5)	0.2544(6)	0.042(2)
N1	0.8807(2)	0.95654(18)	0.05160(19)	0.0294(6)
N2	0.0877(2)	0.12298(17)	0.10323(18)	0.0280(6)
C1	0.7799(3)	0.8681(2)	0.0164(2)	0.0291(7)
C2	0.7137(8)	0.8610(6)	0.0775(6)	0.0278(16)
C2A	0.732(2)	0.8676(13)	0.0824(15)	0.03
C3	0.7852(3)	0.9556(2)	0.1531(2)	0.0315(7)
C4	0.7682(3)	0.9930(2)	0.2297(2)	0.0316(7)
C5	0.8516(3)	0.0890(2)	0.2927(2)	0.0312(7)
C6	0.9485(3)	0.1400(2)	0.2728(2)	0.0310(7)
C7	0.9626(3)	0.0988(2)	0.1916(2)	0.0300(7)
C8	0.8807(3)	0.0066(2)	0.1312(2)	0.0290(7)
C9	0.8399(3)	0.1326(2)	0.3727(2)	0.0319(7)
C10	0.9203(3)	0.2240(2)	0.4344(2)	0.0324(7)
C11	0.0151(3)	0.2729(2)	0.4149(2)	0.0316(7)
C12	0.0323(3)	0.2342(2)	0.3367(2)	0.0307(7)
C13	0.1330(3)	0.2860(2)	0.3200(2)	0.0325(7)
C14	0.1450(3)	0.2440(2)	0.2415(2)	0.0312(7)
C15	0.2322(11)	0.2704(4)	0.2075(7)	0.0297(18)
C15A	0.236(2)	0.2854(12)	0.2061(17)	0.03
C16	0.1958(3)	0.2012(2)	0.1226(2)	0.0296(7)
C17	0.0599(3)	0.1507(2)	0.1755(2)	0.0306(7)
C18	0.9128(3)	0.2712(2)	0.5250(2)	0.0358(7)
C19	0.9131(3)	0.3699(2)	0.5276(3)	0.0398(8)
C20	0.0243(3)	0.2827(3)	0.6109(2)	0.0410(8)
C21	0.7980(3)	0.2122(3)	0.5317(3)	0.0434(8)
C22	0.6664(3)	0.9379(2)	0.2496(2)	0.0339(7)
C23	0.5477(3)	0.9267(2)	0.1964(3)	0.0388(8)
C24	0.4565(3)	0.8803(2)	0.2195(3)	0.0452(9)
C25	0.4784(4)	0.8441(2)	0.2942(3)	0.0469(9)
C26	0.5952(3)	0.8529(2)	0.3438(3)	0.0440(9)
C27	0.6899(3)	0.8992(2)	0.3232(3)	0.0383(8)
C28	0.5185(3)	0.9654(3)	0.1149(3)	0.0477(9)

	x/a	y/b	z/c	U(eq)
C29	0.3776(4)	0.7983(3)	0.3212(4)	0.0603(12)
C30	0.8151(3)	0.9040(3)	0.3769(3)	0.0493(9)
C31	0.2231(3)	0.3835(2)	0.3872(2)	0.0316(7)
C32	0.2228(3)	0.4638(2)	0.3606(2)	0.0343(7)
C33	0.3040(3)	0.5539(2)	0.4260(3)	0.0390(8)
C34	0.3839(3)	0.5666(2)	0.5170(3)	0.0420(9)
C35	0.3838(3)	0.4858(2)	0.5410(3)	0.0406(8)
C36	0.3062(3)	0.3940(2)	0.4777(2)	0.0353(7)
C37	0.1346(3)	0.4552(3)	0.2651(3)	0.0425(8)
C38	0.4657(3)	0.6653(3)	0.5885(3)	0.0574(11)
C39	0.3116(3)	0.3092(3)	0.5077(3)	0.0446(8)
C40	0.7414(3)	0.7948(2)	0.9344(2)	0.0293(7)
C41	0.6262(3)	0.7086(2)	0.9007(2)	0.0302(7)
C42	0.5156(3)	0.7176(2)	0.8642(2)	0.0322(7)
C43	0.4066(3)	0.6393(2)	0.8264(2)	0.0346(7)
C44	0.4088(3)	0.5503(2)	0.8219(2)	0.0364(8)
C45	0.5171(3)	0.5379(2)	0.8568(2)	0.0344(7)
C46	0.6255(3)	0.6194(2)	0.8970(2)	0.0322(7)
C47	0.2844(3)	0.6493(2)	0.7925(3)	0.0393(8)
C48	0.2957(3)	0.7437(3)	0.7768(4)	0.0607(12)
C50	0.1900(3)	0.5690(3)	0.7007(3)	0.0531(10)
C49	0.2408(4)	0.6442(4)	0.8706(3)	0.0630(12)
C51	0.5202(3)	0.4394(2)	0.8478(2)	0.0363(7)
C52	0.3933(4)	0.3582(3)	0.8039(3)	0.0561(11)
C53	0.5910(4)	0.4357(3)	0.9453(3)	0.0544(10)
C54	0.5844(4)	0.4209(3)	0.7857(3)	0.0523(10)
C1X	0.9155(5)	0.2559(3)	0.9493(3)	0.0696(13)
C2X	0.8939(7)	0.3358(5)	0.9403(4)	0.109(2)
C3X	0.9530(9)	0.3940(5)	0.9034(5)	0.131(3)
C4X	0.0349(8)	0.3758(5)	0.8740(5)	0.117(3)
C5X	0.0555(5)	0.2937(5)	0.8786(4)	0.0976(19)
C6X	0.9943(4)	0.2347(4)	0.9164(4)	0.0699(13)
C7X	0.8528(5)	0.1940(4)	0.9916(4)	0.0787(14)
C1Y	0.2292(4)	0.0124(3)	0.3379(3)	0.0577(11)
C2Y	0.3406(4)	0.0330(3)	0.4113(3)	0.0585(11)
C3Y	0.4467(4)	0.0675(3)	0.4018(3)	0.0642(12)

	x/a	y/b	z/c	U(eq)
C4Y	0.4456(4)	0.0827(3)	0.3197(4)	0.0632(12)
C5Y	0.3350(5)	0.0620(3)	0.2444(4)	0.0626(12)
C6Y	0.2297(4)	0.0267(3)	0.2555(3)	0.0621(11)
C7Y	0.1119(5)	0.9744(4)	0.3464(4)	0.0826(16)

Table S18. Band length **(Å)** and Bond angles (°) for **11b**

Ni1-N2	1.959(2)	Ni1-N2	1.959(2)
Ni1-N1	1.978(2)	Ni1-N1	1.978(2)
O1-C2	1.192(7)	O2-C15A	1.21(2)
N1-C8	1.356(4)	N1-C1	1.411(4)
N2-C17	1.353(4)	N2-C16	1.422(4)
C1-C40	1.376(4)	C1-C2A	1.389(10)
C1-C2	1.495(6)	C2-C3	1.481(6)
C2A-C3	1.385(10)	C2A-H2A	0.95
C3-C4	1.366(4)	C3-C8	1.433(4)
C4-C5	1.446(4)	C4-C22	1.496(4)
C5-C9	1.404(5)	C5-C6	1.426(4)
C6-C7	1.427(5)	C6-C12	1.432(4)
C7-C8	1.392(4)	C7-C17	1.396(4)
C9-C10	1.386(5)	C9-H9	0.97(3)
C10-C11	1.391(4)	C10-C18	1.539(5)
C11-C12	1.397(5)	C11-H11	0.95
C12-C13	1.437(4)	C13-C14	1.378(5)
C13-C31	1.495(4)	C14-C15	1.391(6)
C14-C17	1.435(4)	C14-C15A	1.489(10)
C15-C16	1.381(7)	C15-H15	0.95
C15A-C16	1.484(10)	C16-C40	1.406(4)
C18-C21	1.528(4)	C18-C19	1.531(5)
C18-C20	1.536(5)	C19-H19A	0.98
C19-H19B	0.98	C19-H19C	0.98
C20-H20A	0.98	C20-H20B	0.98
C20-H20C	0.98	C21-H21A	0.98
C21-H21B	0.98	C21-H21C	0.98
C22-C27	1.402(5)	C22-C23	1.403(5)
C23-C24	1.380(5)	C23-C28	1.514(5)

C24-C25	1.392(6)	C24-H24	0.95
C25-C26	1.384(5)	C25-C29	1.516(5)
C26-C27	1.388(5)	C26-H26	0.95
C27-C30	1.511(5)	C28-H28A	0.98
C28-H28B	0.98	C28-H28C	0.98
C29-H29A	0.98	C29-H29B	0.98
C29-H29C	0.98	C30-H30A	0.98
C30-H30B	0.98	C30-H30C	0.98
C31-C32	1.397(5)	C31-C36	1.411(5)
C32-C33	1.391(5)	C32-C37	1.504(5)
C33-C34	1.393(5)	C33-H33	0.95
C34-C35	1.384(5)	C34-C38	1.509(5)
C35-C36	1.391(5)	C35-H35	0.95
C36-C39	1.501(5)	C37-H37A	0.98
C37-H37B	0.98	C37-H37C	0.98
C38-H38A	0.98	C38-H38B	0.98
C38-H38C	0.98	C39-H39A	0.98
C39-H39B	0.98	C39-H39C	0.98
C40-C16	1.406(4)	C40-C41	1.493(4)
C41-C46	1.377(4)	C41-C42	1.402(4)
C42-C43	1.376(4)	C42-H42	0.95
C43-C44	1.386(5)	C43-C47	1.544(5)
C44-C45	1.400(5)	C44-H44	0.95
C45-C46	1.395(4)	C45-C51	1.529(5)
C46-H46	0.95	C47-C48	1.512(5)
C47-C50	1.524(5)	C47-C49	1.534(5)
C48-H48A	0.98	C48-H48B	0.98
C48-H48C	0.98	C50-H50A	0.98
C50-H50B	0.98	C50-H50C	0.98
C49-H49A	0.98	C49-H49B	0.98
C49-H49C	0.98	C51-C54	1.530(5)
C51-C53	1.531(5)	C51-C52	1.533(5)
C52-H52A	0.98	C52-H52B	0.98
C52-H52C	0.98	C53-H53A	0.98
C53-H53B	0.98	C53-H53C	0.98
C54-H54A	0.98	C54-H54B	0.98
C54-H54C	0.98	C1X-C2X	1.380(7)

C1X-C6X	1.384(7)	C1X-C7X	1.473(7)
C2X-C3X	1.351(10)	C2X-H2X	0.95
C3X-C4X	1.371(11)	C3X-H3X	0.95
C4X-C5X	1.394(10)	C4X-H4X	0.95
C5X-C6X	1.384(7)	C5X-H5X	0.95
C6X-H6X	0.95	C7X-H7X1	0.98
C7X-H7X2	0.98	C7X-H7X3	0.98
C1Y-C6Y	1.374(6)	C1Y-C2Y	1.393(6)
C1Y-C7Y	1.503(6)	C2Y-C3Y	1.373(6)
C2Y-H2Y	0.95	C3Y-C4Y	1.369(7)
C3Y-H3Y	0.95	C4Y-C5Y	1.401(7)
C4Y-H4Y	0.95	C5Y-C6Y	1.380(6)
C5Y-H5Y	0.95	C6Y-H6Y	0.95
C7Y-H7Y1	0.98	C7Y-H7Y2	0.98
C7Y-H7Y3	0.98		

N2-Ni1-N2	180.0	N2-Ni1-N1	89.05(10)
N2-Ni1-N1	90.95(10)	N2-Ni1-N1	90.95(10)
N2-Ni1-N1	89.05(10)	N1-Ni1-N1	180.0
C8-N1-C1	105.0(2)	C8-N1-Ni1	126.4(2)
C1-N1-Ni1	128.6(2)	C17-N2-C16	104.1(2)
C17-N2-Ni1	126.7(2)	C16-N2-Ni1	129.2(2)
C40-C1-C2A	127.0(6)	C40-C1-N1	125.9(3)
C2A-C1-N1	107.0(6)	C40-C1-C2	121.5(3)
N1-C1-C2	112.6(3)	O1-C2-C3	128.7(4)
O1-C2-C1	129.3(4)	C3-C2-C1	101.6(4)
C3-C2A-C1	112.5(11)	C3-C2A-H2A	123.8
C1-C2A-H2A	123.8	C4-C3-C2A	135.4(6)
C4-C3-C8	123.6(3)	C2A-C3-C8	100.8(6)
C4-C3-C2	129.7(3)	C8-C3-C2	106.7(3)
C3-C4-C5	118.4(3)	C3-C4-C22	122.1(3)
C5-C4-C22	119.6(3)	C9-C5-C6	119.6(3)
C9-C5-C4	121.8(3)	C6-C5-C4	118.6(3)
C5-C6-C7	121.4(3)	C5-C6-C12	118.3(3)
C7-C6-C12	120.4(3)	C8-C7-C17	121.3(3)
C8-C7-C6	119.2(3)	C17-C7-C6	119.4(3)
N1-C8-C7	127.2(3)	N1-C8-C3	114.1(3)

C7-C8-C3	118.8(3)	C10-C9-C5	122.4(3)
C10-C9-H9	123.(2)	C5-C9-H9	115.(2)
C9-C10-C11	117.7(3)	C9-C10-C18	123.2(3)
C11-C10-C18	119.1(3)	C10-C11-C12	123.1(3)
C10-C11-H11	118.5	C12-C11-H11	118.5
C11-C12-C6	119.0(3)	C11-C12-C13	121.5(3)
C6-C12-C13	119.5(3)	C14-C13-C12	118.8(3)
C14-C13-C31	120.9(3)	C12-C13-C31	120.3(3)
C13-C14-C15	133.9(4)	C13-C14-C17	122.3(3)
C15-C14-C17	103.8(3)	C13-C14-C15A	128.7(5)
C17-C14-C15A	108.9(5)	C16-C15-C14	109.0(6)
C16-C15-H15	125.5	C14-C15-H15	125.5
O2-C15A-C16	130.1(14)	O2-C15A-C14	125.1(13)
C16-C15A-C14	98.8(8)	C15-C16-C40	125.2(4)
C15-C16-N2	110.0(3)	C40-C16-N2	124.7(3)
C40-C16-C15A	120.1(5)	N2-C16-C15A	115.0(5)
N2-C17-C7	127.5(3)	N2-C17-C14	112.9(3)
C7-C17-C14	119.6(3)	C21-C18-C19	107.7(3)
C21-C18-C20	109.1(3)	C19-C18-C20	109.1(3)
C21-C18-C10	112.5(3)	C19-C18-C10	110.1(3)
C20-C18-C10	108.3(3)	C18-C19-H19A	109.5
C18-C19-H19B	109.5	H19A-C19-H19B	109.5
C18-C19-H19C	109.5	H19A-C19-H19C	109.5
H19B-C19-H19C	109.5	C18-C20-H20A	109.5
C18-C20-H20B	109.5	H20A-C20-H20B	109.5
C18-C20-H20C	109.5	H20A-C20-H20C	109.5
H20B-C20-H20C	109.5	C18-C21-H21A	109.5
C18-C21-H21B	109.5	H21A-C21-H21B	109.5
C18-C21-H21C	109.5	H21A-C21-H21C	109.5
H21B-C21-H21C	109.5	C27-C22-C23	119.7(3)
C27-C22-C4	120.0(3)	C23-C22-C4	120.3(3)
C24-C23-C22	119.2(3)	C24-C23-C28	119.7(3)
C22-C23-C28	121.2(3)	C23-C24-C25	122.1(4)
C23-C24-H24	119.0	C25-C24-H24	119.0
C26-C25-C24	117.9(3)	C26-C25-C29	121.3(4)
C24-C25-C29	120.7(4)	C25-C26-C27	121.9(4)
C25-C26-H26	119.0	C27-C26-H26	119.0

C26-C27-C22	119.1(3)	C26-C27-C30	120.1(3)
C22-C27-C30	120.8(3)	C23-C28-H28A	109.5
C23-C28-H28B	109.5	H28A-C28-H28B	109.5
C23-C28-H28C	109.5	H28A-C28-H28C	109.5
H28B-C28-H28C	109.5	C25-C29-H29A	109.5
C25-C29-H29B	109.5	H29A-C29-H29B	109.5
C25-C29-H29C	109.5	H29A-C29-H29C	109.5
H29B-C29-H29C	109.5	C27-C30-H30A	109.5
C27-C30-H30B	109.5	H30A-C30-H30B	109.5
C27-C30-H30C	109.5	H30A-C30-H30C	109.5
H30B-C30-H30C	109.5	C32-C31-C36	120.4(3)
C32-C31-C13	119.6(3)	C36-C31-C13	119.9(3)
C33-C32-C31	118.5(3)	C33-C32-C37	119.7(3)
C31-C32-C37	121.8(3)	C32-C33-C34	122.3(3)
C32-C33-H33	118.8	C34-C33-H33	118.8
C35-C34-C33	118.0(3)	C35-C34-C38	120.7(4)
C33-C34-C38	121.2(4)	C34-C35-C36	121.9(3)
C34-C35-H35	119.0	C36-C35-H35	119.0
C35-C36-C31	118.7(3)	C35-C36-C39	119.4(3)
C31-C36-C39	121.9(3)	C32-C37-H37A	109.5
C32-C37-H37B	109.5	H37A-C37-H37B	109.5
C32-C37-H37C	109.5	H37A-C37-H37C	109.5
H37B-C37-H37C	109.5	C34-C38-H38A	109.5
C34-C38-H38B	109.5	H38A-C38-H38B	109.5
C34-C38-H38C	109.5	H38A-C38-H38C	109.5
H38B-C38-H38C	109.5	C36-C39-H39A	109.5
C36-C39-H39B	109.5	H39A-C39-H39B	109.5
C36-C39-H39C	109.5	H39A-C39-H39C	109.5
H39B-C39-H39C	109.5	C1-C40-C16	122.4(3)
C1-C40-C41	120.2(3)	C16-C40-C41	117.3(3)
C46-C41-C42	119.3(3)	C46-C41-C40	121.7(3)
C42-C41-C40	118.8(3)	C43-C42-C41	121.2(3)
C43-C42-H42	119.4	C41-C42-H42	119.4
C42-C43-C44	118.1(3)	C42-C43-C47	121.7(3)
C44-C43-C47	120.1(3)	C43-C44-C45	122.6(3)
C43-C44-H44	118.7	C45-C44-H44	118.7
C46-C45-C44	117.5(3)	C46-C45-C51	119.7(3)

C44-C45-C51	122.8(3)	C41-C46-C45	121.3(3)
C41-C46-H46	119.3	C45-C46-H46	119.3
C48-C47-C50	108.3(3)	C48-C47-C49	107.8(3)
C50-C47-C49	109.5(3)	C48-C47-C43	112.6(3)
C50-C47-C43	111.2(3)	C49-C47-C43	107.3(3)
C47-C48-H48A	109.5	C47-C48-H48B	109.5
H48A-C48-H48B	109.5	C47-C48-H48C	109.5
H48A-C48-H48C	109.5	H48B-C48-H48C	109.5
C47-C50-H50A	109.5	C47-C50-H50B	109.5
H50A-C50-H50B	109.5	C47-C50-H50C	109.5
H50A-C50-H50C	109.5	H50B-C50-H50C	109.5
C47-C49-H49A	109.5	C47-C49-H49B	109.5
H49A-C49-H49B	109.5	C47-C49-H49C	109.5
H49A-C49-H49C	109.5	H49B-C49-H49C	109.5
C45-C51-C54	109.3(3)	C45-C51-C53	110.3(3)
C54-C51-C53	108.2(3)	C45-C51-C52	112.7(3)
C54-C51-C52	108.5(3)	C53-C51-C52	107.8(3)
C51-C52-H52A	109.5	C51-C52-H52B	109.5
H52A-C52-H52B	109.5	C51-C52-H52C	109.5
H52A-C52-H52C	109.5	H52B-C52-H52C	109.5
C51-C53-H53A	109.5	C51-C53-H53B	109.5
H53A-C53-H53B	109.5	C51-C53-H53C	109.5
H53A-C53-H53C	109.5	H53B-C53-H53C	109.5
C51-C54-H54A	109.5	C51-C54-H54B	109.5
H54A-C54-H54B	109.5	C51-C54-H54C	109.5
H54A-C54-H54C	109.5	H54B-C54-H54C	109.5
C2X-C1X-C6X	118.9(6)	C2X-C1X-C7X	119.8(6)
C6X-C1X-C7X	121.3(4)	C3X-C2X-C1X	120.1(8)
C3X-C2X-H2X	120.0	C1X-C2X-H2X	120.0
C2X-C3X-C4X	121.5(7)	C2X-C3X-H3X	119.2
C4X-C3X-H3X	119.2	C3X-C4X-C5X	120.0(6)
C3X-C4X-H4X	120.0	C5X-C4X-H4X	120.0
C6X-C5X-C4X	117.8(7)	C6X-C5X-H5X	121.1
C4X-C5X-H5X	121.1	C5X-C6X-C1X	121.6(6)
C5X-C6X-H6X	119.2	C1X-C6X-H6X	119.2
C1X-C7X-H7X1	109.5	C1X-C7X-H7X2	109.5
H7X1-C7X-H7X2	109.5	C1X-C7X-H7X3	109.5

H7X1-C7X-H7X3	109.5	H7X2-C7X-H7X3	109.5
C6Y-C1Y-C2Y	117.4(4)	C6Y-C1Y-C7Y	120.4(4)
C2Y-C1Y-C7Y	122.2(4)	C3Y-C2Y-C1Y	121.1(4)
C3Y-C2Y-H2Y	119.4	C1Y-C2Y-H2Y	119.4
C4Y-C3Y-C2Y	120.7(4)	C4Y-C3Y-H3Y	119.7
C2Y-C3Y-H3Y	119.7	C3Y-C4Y-C5Y	119.5(4)
C3Y-C4Y-H4Y	120.2	C5Y-C4Y-H4Y	120.2
C6Y-C5Y-C4Y	118.6(4)	C6Y-C5Y-H5Y	120.7
C4Y-C5Y-H5Y	120.7	C1Y-C6Y-C5Y	122.6(5)
C1Y-C6Y-H6Y	118.7	C5Y-C6Y-H6Y	118.7
C1Y-C7Y-H7Y1	109.5	C1Y-C7Y-H7Y2	109.5
H7Y1-C7Y-H7Y2	109.5	C1Y-C7Y-H7Y3	109.5
H7Y1-C7Y-H7Y3	109.5	H7Y2-C7Y-H7Y3	109.5

Table S19. Anisotropic atomic displacement parameters ($Å^2$) for **11b**, The anisotropic atomic displacement factor exponent takes the form: $-2\pi^2[h^2 a^{*2} U_{11} + ... + 2 h k a^* b^* U_{12}]$

	**U_{11}**	**U_{22}**	**U_{33}**	**U_{23}**	**U_{13}**	**U_{12}**
Ni1	0.0260(4)	0.0264(4)	0.0249(4)	0.0014(3)	0.0105(3)	0.0054(3)
O1	0.038(2)	0.0299(18)	0.034(2)	-0.0025(14)	0.0198(16)	-0.0012(15)
O2	0.033(4)	0.034(4)	0.034(5)	-0.009(3)	0.014(4)	-0.004(3)
N1	0.0257(13)	0.0290(13)	0.0289(15)	0.0031(11)	0.0132(11)	0.0077(10)
N2	0.0274(13)	0.0309(13)	0.0202(14)	0.0031(10)	0.0103(11)	0.0082(11)
C1	0.0264(15)	0.0284(16)	0.0304(18)	0.0072(13)	0.0130(13)	0.0093(12)
C2	0.019(4)	0.023(2)	0.030(3)	-0.0007(18)	0.010(3)	0.001(2)
C3	0.0294(16)	0.0285(16)	0.0314(19)	0.0048(13)	0.0132(14)	0.0083(13)
C4	0.0318(16)	0.0275(16)	0.0317(18)	0.0068(13)	0.0133(14)	0.0096(13)
C5	0.0305(16)	0.0301(16)	0.0305(18)	0.0067(13)	0.0126(14)	0.0118(13)
C6	0.0275(15)	0.0287(16)	0.0299(18)	0.0063(13)	0.0094(13)	0.0083(13)
C7	0.0299(16)	0.0278(16)	0.0294(18)	0.0072(13)	0.0117(13)	0.0107(13)
C8	0.0281(15)	0.0284(16)	0.0288(18)	0.0065(13)	0.0121(13)	0.0114(13)
C9	0.0304(16)	0.0301(16)	0.0333(19)	0.0064(14)	0.0155(14)	0.0106(13)
C10	0.0327(16)	0.0330(17)	0.0303(18)	0.0073(14)	0.0134(14)	0.0140(13)
C11	0.0308(16)	0.0287(16)	0.0256(17)	0.0026(13)	0.0082(13)	0.0082(13)
C12	0.0298(16)	0.0279(16)	0.0298(18)	0.0052(13)	0.0117(14)	0.0098(13)
C13	0.0287(16)	0.0300(16)	0.0318(19)	0.0058(13)	0.0099(14)	0.0093(13)
C14	0.0265(15)	0.0257(16)	0.0325(19)	0.0039(13)	0.0095(14)	0.0065(12)
C15	0.030(2)	0.017(3)	0.037(3)	0.004(3)	0.016(2)	0.004(3)

	U_{11}	U_{22}	U_{33}	U_{23}	U_{13}	U_{12}
C16	0.0260(15)	0.0290(16)	0.0289(17)	0.0061(13)	0.0111(13)	0.0080(12)
C17	0.0279(15)	0.0292(16)	0.0278(18)	0.0049(13)	0.0098(13)	0.0083(13)
C18	0.0362(17)	0.0351(17)	0.0303(19)	0.0033(14)	0.0145(15)	0.0118(14)
C19	0.0396(18)	0.0352(18)	0.041(2)	0.0013(15)	0.0197(16)	0.0147(15)
C20	0.0426(19)	0.048(2)	0.0276(19)	0.0059(15)	0.0132(15)	0.0181(16)
C21	0.045(2)	0.0411(19)	0.038(2)	0.0029(15)	0.0221(17)	0.0106(16)
C22	0.0362(17)	0.0258(16)	0.0358(19)	0.0018(13)	0.0182(15)	0.0098(13)
C23	0.0351(18)	0.0321(17)	0.040(2)	0.0020(14)	0.0162(15)	0.0084(14)
C24	0.0376(19)	0.0347(18)	0.059(3)	0.0052(17)	0.0254(18)	0.0101(15)
C25	0.055(2)	0.0306(18)	0.058(3)	0.0051(16)	0.038(2)	0.0114(16)
C26	0.061(2)	0.0347(18)	0.048(2)	0.0145(16)	0.035(2)	0.0201(17)
C27	0.0466(19)	0.0303(17)	0.040(2)	0.0089(14)	0.0238(16)	0.0149(15)
C28	0.0321(18)	0.053(2)	0.051(2)	0.0159(18)	0.0137(17)	0.0142(16)
C29	0.064(3)	0.044(2)	0.085(3)	0.015(2)	0.054(3)	0.0157(19)
C30	0.054(2)	0.047(2)	0.053(3)	0.0224(18)	0.026(2)	0.0200(18)
C31	0.0261(15)	0.0328(17)	0.0295(18)	0.0017(13)	0.0138(14)	0.0072(13)
C32	0.0292(16)	0.0323(17)	0.040(2)	0.0069(14)	0.0187(15)	0.0101(13)
C33	0.0363(18)	0.0313(17)	0.050(2)	0.0080(15)	0.0234(17)	0.0123(14)
C34	0.0304(17)	0.0346(18)	0.045(2)	-0.0064(15)	0.0155(16)	0.0063(14)
C35	0.0350(18)	0.042(2)	0.033(2)	0.0003(15)	0.0112(15)	0.0128(15)
C36	0.0315(17)	0.0392(18)	0.0286(19)	0.0031(14)	0.0125(14)	0.0113(14)
C37	0.0396(19)	0.0425(19)	0.042(2)	0.0109(16)	0.0161(16)	0.0161(16)
C38	0.044(2)	0.037(2)	0.061(3)	-0.0120(18)	0.017(2)	0.0010(17)
C39	0.044(2)	0.045(2)	0.036(2)	0.0078(16)	0.0115(16)	0.0165(16)
C40	0.0291(16)	0.0296(16)	0.0261(17)	0.0063(13)	0.0108(13)	0.0111(13)
C41	0.0307(16)	0.0301(16)	0.0253(17)	0.0030(12)	0.0135(13)	0.0090(13)
C42	0.0358(17)	0.0273(16)	0.0306(18)	0.0050(13)	0.0165(14)	0.0097(13)
C43	0.0300(16)	0.0351(17)	0.0337(19)	0.0078(14)	0.0134(14)	0.0093(13)
C44	0.0309(17)	0.0321(17)	0.037(2)	0.0062(14)	0.0123(14)	0.0064(13)
C45	0.0360(17)	0.0304(17)	0.0311(19)	0.0052(13)	0.0156(15)	0.0080(13)
C46	0.0288(16)	0.0331(17)	0.0299(18)	0.0056(13)	0.0125(14)	0.0095(13)
C47	0.0275(16)	0.0396(19)	0.040(2)	0.0100(15)	0.0082(15)	0.0095(14)
C48	0.035(2)	0.047(2)	0.082(3)	0.020(2)	0.007(2)	0.0147(17)
C50	0.038(2)	0.049(2)	0.052(3)	0.0050(18)	0.0056(18)	0.0138(17)
C49	0.043(2)	0.103(4)	0.052(3)	0.028(3)	0.022(2)	0.039(2)
C51	0.0402(18)	0.0293(17)	0.0312(19)	0.0055(13)	0.0123(15)	0.0101(14)

	U_{11}	U_{22}	U_{33}	U_{23}	U_{13}	U_{12}
C52	0.051(2)	0.031(2)	0.076(3)	0.0121(19)	0.024(2)	0.0105(17)
C53	0.071(3)	0.041(2)	0.045(2)	0.0143(17)	0.016(2)	0.0260(19)
C54	0.063(2)	0.039(2)	0.054(3)	0.0067(17)	0.030(2)	0.0191(18)
C1X	0.083(3)	0.062(3)	0.050(3)	0.014(2)	0.011(2)	0.034(2)
C2X	0.184(6)	0.095(4)	0.072(4)	0.036(3)	0.050(4)	0.087(4)
C3X	0.219(8)	0.083(4)	0.085(5)	0.030(3)	0.054(5)	0.063(5)
C4X	0.149(6)	0.084(4)	0.062(4)	0.032(3)	0.023(4)	-0.002(4)
C5X	0.084(4)	0.106(4)	0.060(3)	0.030(3)	0.011(3)	0.007(3)
C6X	0.057(3)	0.070(3)	0.065(3)	0.026(2)	0.010(2)	0.020(2)
C7X	0.078(3)	0.096(4)	0.069(4)	0.028(3)	0.025(3)	0.051(3)
C1Y	0.069(3)	0.054(2)	0.060(3)	0.026(2)	0.028(2)	0.034(2)
C2Y	0.075(3)	0.042(2)	0.044(3)	0.0124(18)	0.017(2)	0.017(2)
C3Y	0.060(3)	0.045(2)	0.057(3)	0.005(2)	0.011(2)	0.007(2)
C4Y	0.065(3)	0.044(2)	0.070(3)	0.008(2)	0.030(2)	0.015(2)
C5Y	0.087(3)	0.055(3)	0.064(3)	0.022(2)	0.040(3)	0.042(2)
C6Y	0.074(3)	0.076(3)	0.057(3)	0.030(2)	0.030(2)	0.049(3)
C7Y	0.085(4)	0.109(4)	0.084(4)	0.057(4)	0.044(3)	0.056(3)

Table S20. Hydrogen atomic coordinates and isotropic atomic displacement parameters (Å^2) for **11b**

	**x/a**	**y/b**	**z/c**	**U(eq)**
H2A	0.6697	0.8130	0.0795	0.03
H9	0.770(3)	1.095(3)	0.380(3)	0.038
H11	1.0707	1.3357	0.4566	0.038
H15	1.3050	1.3270	0.2376	0.036
H19A	0.8404	1.3627	0.4745	0.06
H19B	0.9855	1.4091	0.5227	0.06
H19C	0.9136	1.4011	0.5874	0.06
H20A	1.0210	1.3125	0.6693	0.061
H20B	1.0982	1.3233	0.6087	0.061
H20C	1.0249	1.2198	0.6093	0.061
H21A	0.7265	1.2044	0.4770	0.065
H21B	0.7971	1.2453	0.5901	0.065
H21C	0.7968	1.1492	0.5324	0.065
H24	0.3762	0.8728	0.1834	0.054
H26	0.6112	0.8264	0.3934	0.053

	x/a	y/b	z/c	U(eq)
H28A	0.5089	0.9201	0.0581	0.072
H28B	0.5846	1.0270	0.1312	0.072
H28C	0.4429	0.9740	0.1027	0.072
H29A	0.4052	0.7670	0.3670	0.09
H29B	0.3065	0.7507	0.2645	0.09
H29C	0.3560	0.8476	0.3497	0.09
H30A	0.8127	0.8683	0.4212	0.074
H30B	0.8719	0.9709	0.4123	0.074
H30C	0.8418	0.8759	0.3322	0.074
H33	1.3050	1.6087	0.4079	0.047
H35	1.4385	1.4932	0.6026	0.049
H37A	1.1560	1.4278	0.2161	0.064
H37B	1.1380	1.5187	0.2643	0.064
H37C	1.0524	1.4134	0.2527	0.064
H38A	1.4511	1.6709	0.6453	0.086
H38B	1.4486	1.7133	0.5611	0.086
H38C	1.5506	1.6757	0.6054	0.086
H39A	1.3928	1.3269	0.5570	0.067
H39B	1.2939	1.2566	0.4530	0.067
H39C	1.2514	1.2889	0.5326	0.067
H42	0.5159	0.7789	-0.1343	0.039
H44	0.3340	0.4956	-0.2060	0.044
H46	0.7004	0.6133	-0.0777	0.039
H48A	0.3281	0.7504	-0.2702	0.091
H48B	0.3506	0.7962	-0.1634	0.091
H48C	0.2156	0.7458	-0.2463	0.091
H50A	0.1156	0.5799	-0.3218	0.08
H50B	0.1721	0.5076	-0.2879	0.08
H50C	0.2215	0.5679	-0.3473	0.08
H49A	0.3008	0.6967	-0.0707	0.095
H49B	0.2310	0.5829	-0.1188	0.095
H49C	0.1629	0.6499	-0.1488	0.095
H52A	0.3521	0.3676	-0.1557	0.084
H52B	0.4003	0.2968	-0.2014	0.084
H52C	0.3463	0.3583	-0.2592	0.084
H53A	0.6746	0.4838	-0.0270	0.082

	x/a	y/b	z/c	U(eq)
H53B	0.5913	0.3720	-0.0613	0.082
H53C	0.5523	0.4488	-0.0133	0.082
H54A	0.5438	0.4274	-0.2757	0.078
H54B	0.5815	0.3561	-0.2240	0.078
H54C	0.6692	0.4675	-0.1830	0.078
H2X	0.8374	0.3499	0.9600	0.131
H3X	0.9375	0.4489	0.8978	0.157
H4X	1.0776	0.4191	0.8505	0.141
H5X	1.1097	0.2789	0.8564	0.117
H6X	1.0067	0.1782	0.9199	0.084
H7X1	0.9032	0.2148	1.0599	0.118
H7X2	0.8384	0.1276	0.9624	0.118
H7X3	0.7750	0.1983	0.9807	0.118
H2Y	0.3432	0.0231	0.4689	0.07
H3Y	0.5215	0.0810	0.4527	0.077
H4Y	0.5194	0.1071	0.3138	0.076
H5Y	0.3326	0.0720	0.1869	0.075
H6Y	0.1545	0.0118	0.2042	0.074
H7Y1	0.0662	0.0129	0.3281	0.124
H7Y2	0.1290	-0.0223	0.4121	0.124
H7Y3	0.0639	-0.0923	0.3047	0.124
